# Palindrome-mediated 16p13.3 triplications cause a recognizable neurodegenerative disorder with ataxia

**DOI:** 10.1016/j.ajhg.2025.11.011

**Published:** 2025-12-04

**Authors:** James Fasham, Julia Rankin, Rachel Schot, Susan M. White, Katrina M. Bell, Matthew N. Wakeling, Lucy J. Mallin, Alex Shah, Michelle G. de Silva, David I. Francis, Maie Walsh, Emily E. Jones, Kayal Vijayakumar, Katie Johnson, Francis H. Sansbury, Johann te Water Naudé, Paola Giunti, Marios Hadjivassiliou, Andrea H. Nemeth, George K. Tofaris, Carlo Rinaldi, Benito Banos-Pinero, Marianna Selikhva, Nishanka Ubeyratna, Anneke Kievit, Frank Sleutels, Joey van Giessen, Tahsin Stefan Barakat, Timothy S. Hall, Alan Whone, Eleanor Thomas, Joseph S. Leslie, Rosemary A. Bamford, Aaron R. Jeffries, Jenny Lord, Susan Walker, Tjakko J. van Ham, Sue L. Hill, Lucy McGavin, Andrew Parrish, Andrew H. Crosby, Emma L. Baple, Alistair T. Pagnamenta

**Affiliations:** 1 Department of Clinical and Biomedical Sciences, Faculty of Health and Life Sciences, University of Exeter, EX2 5DW Exeter, UK; 2 Department of Clinical Genetics, Royal Devon University Hospital, EX1 2ED Exeter, UK; 3 Department of Clinical Genetics, Erasmus MC University Medical Center, Rotterdam, the Netherlands; 4 Victorian Clinical Genetics Services, Murdoch Children’s Research Institute, Melbourne, Australia; 5 Department of Paediatrics, University of Melbourne, Parkville, VIC, Australia; 6 Murdoch Children’s Research Institute, Melbourne, VIC, Australia; 7 Exeter Genomics Laboratory, Royal Devon University Healthcare NHS Foundation Trust, EX2 5DW Exeter, UK; 8 University Hospitals Plymouth NHS Trust, Derriford Road, Crownhill, Plymouth, PL6 8DH Devon, UK; 9 Genomic Medicine, the Royal Melbourne Hospital, Parkville, VIC, Australia; 10 Bristol Genetics Laboratory, North Bristol NHS Trust, BS10 5NB Bristol, UK; 11 Department of Paediatric Neurology, University Hospitals Bristol NHS Foundation Trust, BS1 3NU Bristol, UK; 12 Nottingham Regional Genetics Service, Nottingham City Hospital Campus, The Gables, NG5 1PB Nottingham, UK; 13 All Wales Medical Genomics Service, NHS Wales Cardiff and Vale University Health Board, Wales Genomic Health Centre, Cardiff Edge Business Park, Longwood Drive, Whitchurch, CF14 7YU Cardiff, UK; 14 Division of Cancer & Genetics, School of Medicine, Cardiff University, CF14 4XN Cardiff, UK; 15 Department of Paediatric Neurology, University Hospital of Wales, CF14 4XW Cardiff, UK; 16 Queen Square Institute of Neurology, WC1N 3BG London, UK; 17 Academic Department of Neurosciences, Sheffield Teaching Hospitals NHS Trust and University of Sheffield, S10 2JF Sheffield, UK; 18 Nuffield Department of Clinical Neurosciences, University of Oxford, OX3 9DU Oxford, UK; 19 Oxford Centre for Genomic Medicine, Oxford University Hospitals NHS Foundation Trust, OX3 7LD Oxford, UK; 20 Department of Clinical Neurology, Oxford University Hospitals NHS Foundation Trust, OX3 9DU Oxford, UK; 21 Institute of Developmental and Regenerative Medicine, University of Oxford, OX3 7TY Oxford, UK; 22 Oxford Genetics Laboratories, Oxford University Hospitals NHS Foundation Trust, OX3 9DU Oxford, UK; 23 Neurology Department, Southmead Hospital, BS10 5NB Bristol, UK; 24 Southmead Hospital, BS10 5NB Bristol, UK; 25 Department of Child Health, Royal Devon University Hospital, EX1 2ED Exeter, UK; 26 Biosciences, University of Exeter, EX1 2LU Exeter, UK; 27 Sheffield Institute for Translational Neuroscience (SITraN), University of Sheffield, S10 2HQ Sheffield, UK; 28 Genomics England, EC1M 6BQ London, UK; 29 NHS England, London, UK; 30 University of Plymouth, PL4 8AA Plymouth, UK

**Keywords:** cerebellar hypoplasia, duplication-triplication, CNV, ataxia, structural variant, *ATP6V0C*, inversion, gene dosage

## Abstract

Complex neurodegenerative conditions have occasionally been associated with copy-number gains. Using microarray and genome sequencing on DNA samples from eleven individuals from nine unrelated families, we show that copy-number gains at 16p13.3 cause a severe, recognizable disorder characterized by early-onset progressive ataxia and cognitive decline (9–32 years). Most affected individuals also displayed peripheral neuropathy and scoliosis. Optic atrophy, nystagmus, and dystonia were more variable features. The neuroradiological phenotype comprises a distinctive combination of atrophy of the cerebellum and caudate nuclei. Co-segregation data showed that the structural variant (SV) had occurred *de novo* in 5 individuals and, for one other individual, had been inherited from a mosaic, unaffected parent. Triplicated segments of 16p13.3 were identified within the duplications. Although these varied in size (30–811 kb), the minimal region of overlap included a single gene (*ATP6V0C*) that is highly expressed in the cerebellum. RNA sequencing (RNA-seq) using whole-blood and fibroblast/lymphoblast cultures indicated increased expression of several genes within the SV, with *ATP6V0C* showing the most significant increase (up to 4-fold). In most cases, the central segment of the SV was proven to be inverted and lay immediately distal to a 144 kb palindrome. Across 500,000 individuals from the UK Biobank, we identified 19 duplications but no triplications at this locus. Further analysis of the consequences of *ATP6V0C* overexpression on the stoichiometry within the vacuolar H^+^-ATPase heteromer and on neurological function will provide valuable pathomechanistic insights. Together, our findings define palindrome-mediated triplication on 16p13.3 as the cause of a clinically distinct childhood-onset neurodegenerative disorder.

## Graphical abstract

**Figure:**
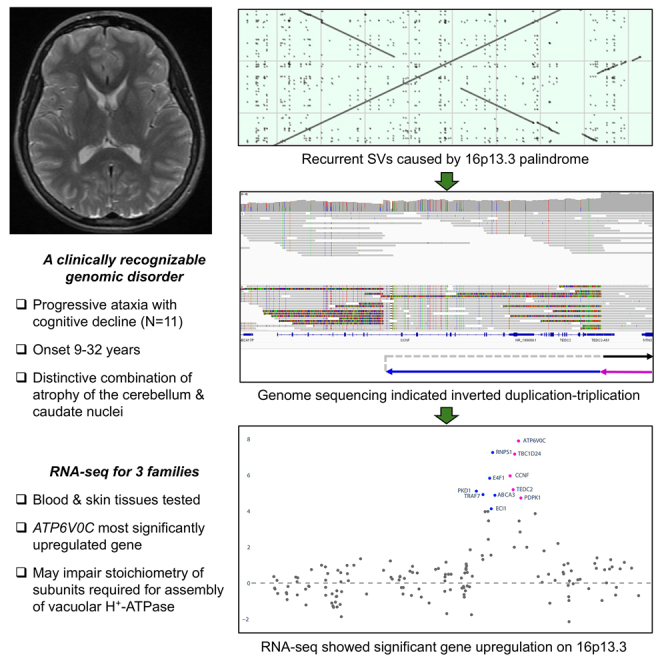

## Teaser

Fasham and colleagues report a palindrome-mediated genomic disorder causing a recognizable, severe phenotype marked by early-onset progressive ataxia, cognitive decline, and cerebellar atrophy. Microarray and short/long-read genome sequencing uncovered overlapping inverted duplication-triplications on 16p13.3. RNA-seq showed upregulation of multiple genes within the variant, with *ATP6V0C* showing the most significant increase.

## Main text

Childhood-onset neurodegenerative disorders comprise a heterogeneous group of rare conditions that present with progressive neurological and/or cognitive impairment.^1^ Features can include regression of motor or cognitive skills, seizures, behavioral changes, ataxia, spasticity, sensory deficits, and intellectual disability. Cerebellar atrophy is a frequent finding with a wide differential diagnosis, including mitochondrial disorders, such as Leigh syndrome (MIM: 256000 and 500017),^2^ ataxia telangiectasia (MIM: 208900),^3^ disorders of neurodegeneration with brain iron accumulation (NBIA),^4^ and others. These conditions are commonly autosomal-recessive or X-linked disorders, caused by loss of function genetic variants. Chromosomal gains involving triplosensitive genes are a rare cause of progressive childhood-onset neurological disorders. Notable examples include whole-gene duplication or triplication of *PLP1* (MIM: 300401) in Pelizaeus-Merzbacher disease (MIM: 312080)^5^ and duplication or, rarely, triplication of *PMP22* (MIM: 601097) in Charcot-Marie-Tooth 1A (MIM: 118220).^6^^,^^7^

Technologies such as microarray and short-read genome sequencing (GS) are well suited to detecting microdeletions and microduplications but often fail to identify complex structures. In some cases, this can lead to diagnostic structural variants (SVs) being overlooked. Advances in computational algorithms for copy-number variant (CNV) and SV detection from GS and long-read sequencing now enable improved characterization of more complex chromosomal rearrangements. One notable example of such a rearrangement is the duplication-triplication. These configurations typically have an inverted central segment and are often mediated by oppositely oriented segmental duplications (SDs).^8^ Complex SVs can also recur at palindromic DNA sequences, as these can promote double-strand breaks. Here, we used a combination of microarray, exome, GS, and RNA sequencing (RNA-seq) technologies to define palindrome-mediated duplication-triplications on 16p13.3 as the cause of a clinically and radiologically distinct early-onset neurodegenerative disorder with ataxia.

We initially identified individual 1, who presented at 10 years of age with childhood-onset, severe, and progressive ataxia, associated with axonal neuropathy and neuroradiological evidence of reduced cerebellar volume (Note S1). Array-based comparative genome hybridization (aCGH) analysis (8x60k constitutive v.3.0, Oxford Gene Technology) followed by parental segregation studies identified a rare *de novo* CN gain of 448 kb involving 16p13.3. The *de novo* nature of this variant prompted us to seek further individuals with similar chromosomal gains through international collaboration, using DECIPHER, GeneMatcher,^9^ and the Genomics England National Genomic Research Library (NGRL; ethical approval from Cambridge South/Central RECs, 14/EE/1112, 20/EE/0035, and 25/EE/0125). Our analysis used Manta and Canvas calls for 100,000 Genomes Project (100kGP) participants, which were analyzed using SVRare.^10^^,^^11^^,^^12^ For NHS Genomic Medicine Service (GMS) data, Dragen CNV calls were assessed with custom scripts. Genomic coordinates are reported using GRCh38.

**Figure 1:**
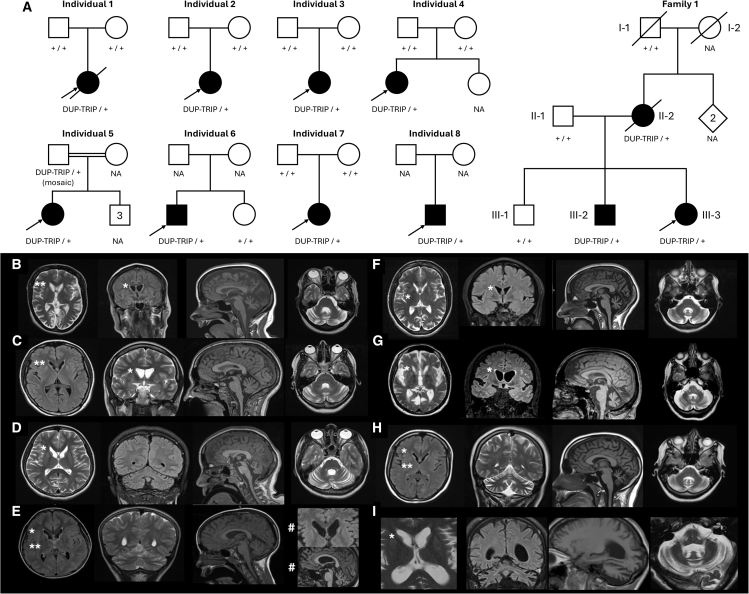
Family structures and MRI data for 11 individuals with overlapping gains on 16p13.3 (A) Pedigrees for 8 unrelated individuals and one multiplex family with overlapping SVs on 16p13.3. Individuals 1–3 were ascertained locally and via the DECIPHER database. Individuals 4–8 were identified from 100kGP and GMS data available in the National Genomic Research Library. Family 1 was ascertained via GeneMatcher, where *ATP6V0C* and *PDPK1* were entered as the candidate genes of interest. + represents the normal copy of 16p13.3. (B–I) MRI scans from individuals 1–8 acquired at ages 36, 18, 9, 10 and 20 (#), 31, 53, 20, and 29. Axial and coronal T2 or FLAIR images demonstrate caudate atrophy of varying degrees (^∗^) in all individuals and a high T2/FLAIR signal in the basal ganglia (^∗∗^) in individuals 1, 2, 4, and 7. Sagittal T1 and axial T2 images (columns 3 and 4) show the cerebellar hemisphere and vermian atrophy in all individuals. There is progressive caudate and cerebellar atrophy in individual 4 (row E). For further MRI images, see Figure S1.

In total, after resolving duplicate individuals identified across studies (Note S2), we ascertained 11 affected individuals from 9 distinct families (Figure 1A) with overlapping CN gains involving 16p13.3, progressive ataxia, and cerebellar atrophy. Phenotype information was obtained with informed consent by clinical care providers using a standardized proforma. Clinical findings for the 11 affected individuals are summarized in Table 1 and Note S1.

**Table 1:** Clinical and neuroradiological findings in individuals with overlapping duplication/triplications involving 16p13.3

**Individual ID (DECIPHER ID)**	**Individual 1 (381641)**	**Individual 2 (339803)**	**Individual 3 (359492)**	**Individual 4 (341728)**	**Individual 5**	**Individual 6**	**Individual 7**	**Individual 8**	**Family 1, II-2**	**Family 1, III-2**	**Family 1, III-3**
Age at last assessment	died at 41 y	27 y	17 y	20 y	40 y	65 y	36 y	40 y	died at 56 y	36 y	27 y
Ancestry^a^	British	Australian^b^	Sri Lankan	British	Pakistani	British	Irish	British	Dutch	Dutch	Dutch
Sex	female	female	female	female	female	male	female	male	female	male	female
SV detection method	array + ES	array + GS	array, ES, LR	array + GS	GS	GS	GS + LR	GS	array + GS	array	array + GS
Duplication size	448 kb	704 kb	640 kb	918 kb	230 kb	157 kb	187 kb	378 kb	514 kb	514 kb	514 kb
Triplication size	30 kb	179 kb	317 kb	811 kb	64 kb	85 kb	105 kb	296 kb	250 kb	250 kb	250 kb
Inheritance	*de novo*	*de novo*	*de novo*	*de novo*	father mosaic	NK	*de novo*	NK	NK^c^	maternal	maternal

**Clinical features**											

Cognitive impairment	yes (<10 y)	mild (5 y)	yes, special education	mild/Mod. with ADHD	yes	significant	moderate	yes, initially normal	yes	yes, special education	yes, special education
Regression (8/10)	yes (in 20s)	no	yes	no	NK	yes	yes	yes (16 y)	yes (9 y)	yes	yes
Progressive ataxia^d^	yes (10 y)	yes (13 y)	yes (9 y)	yes (15 y)	yes	yes (32 y)	yes	yes (23 y)	yes (9 y)	yes (12 y)	yes (12 y)
Dysarthria (10/11)	yes	yes	yes	no	yes	yes	yes	yes	yes	yes (15 y)	yes
Nystagmus (4/11)	no	no	no	yes (15 y)	no	yes	yes	probable	no	no	no
Axonal neuropathy (9/11)	sensory (17 y)	no	yes	no	sensory	sensory	yes	sensory	sensory (46 y)	sensory (15 y)	sensory (9 y)
*Pes cavus* (5/6)	yes	NK	yes	no	NK	yes	NK	yes	NK	NK	yes
Dystonia (2/10)	no	no	no	no	yes	no	yes	no	no	NK	no
Scoliosis/kyphosis (7/10)	no	scoliosis (10 y)	scoliosis	scoliosis (14 y)	scoliosis	no	NK	scoliosis	kyphosis	no	scoliosis
Optic atrophy (2)	NK	NK	NK	NK	NK	yes	NK	no	yes	NK	NK
Other features	seizures suspected	hearing loss	hypertonia	macrocephaly clinodactyly	cataract, CVI, dysphagia	seizures suspected	chorea	–	spasticity	–	hypertonia, strabismus, hearing loss

**Neuroradiology**											

Cerebellar and caudate atrophy (11/11)	yes (35 y, 36 y)	yes (18 y, 23 y)	yes (9 y)	progressive (10 y, 20 y)	yes (31 y)	yes (53 y)	yes (20 y)	yes (29 y)	yes	yes (15 y)	yes (9 y)
Other findings	–	–	–	syrinx T4–T9	–	global atrophy	–	cerebral volume loss	–	–	–

The age at which the phenotype was first recorded is shown in parentheses. CNV sizes are approximate, as in most cases, there remains some uncertainty about the precise coordinates, particularly at the proximal breakpoints. Full genomic coordinates are shown in Table S1. Abs, absent; ADHD, attention-deficit hyperactivity disorder; CVI, cortical visual impairment; ES, exome sequencing; GS, genome sequencing; LR, long-read genome sequencing; Mod., moderate; y, years; NK, not known.

a Ancestry is as per self-report.

b European-Australian.

c Presumed *de novo*; not paternally inherited and no DNA available from the deceased mother.

d Ataxia was diagnosed during childhood in most patients. In others, the diagnosis was later, although it is possible that childhood symptoms preceded any formal diagnosis.

Affected individuals exhibited a shared phenotype characterized by progressive ataxia and cognitive decline. Ataxia, sometimes manifesting with dysarthria (10/11) and nystagmus (4/11), was usually noted in the second decade (range: 9–32 years). Mobility was typically affected, with a walking aid or wheelchair required in the third or fourth decade. Cognitive impairment was also universally present, with the majority (8/10) demonstrating progressive decline. This cognitive deterioration was often accompanied by behavioral challenges and sometimes required supportive care. In individuals 4, 7, and 8, either cognitive delay or ataxia predominated initially, with the other symptoms emerging later. Peripheral neuropathy, typically axonal and sensory predominant, was also common (9/11), as were absent deep tendon reflexes (9/10), *pes cavus* (5/6), and scoliosis/kyphosis (7/10). Additional, more variable neurological features, such as dystonia (2/10) and spasticity (1/9), were also observed. There was no craniofacial dysmorphism and no confirmed clinical or electrophysiological seizures reported. MRI neuroimaging findings were striking and consistent across all affected individuals, with cerebellar volume loss, caudate atrophy, and a high T2/FLAIR signal in the basal ganglia (Figures 1B–1I and S1). Progressive volume loss was also observed where multiple images were available for review (Figure 1E).

The pathogenicity of the overlapping gains identified in these 9 families is supported by inheritance studies (Figure 1A), with 5 confirmed *de novo* (inheritance not determined for 2 families). Family 1 comprised 3 affected individuals. A gain on 16p13.3 was identified through GS in the proband (III-3), and co-segregation studies showed the presence of the SV in her similarly affected brother (III-2) but not in the eldest unaffected sibling (III-1). The SV had been inherited from the affected mother (II-2), but as DNA was unavailable for the maternal grandfather, the inheritance for the mother is unknown.

**Figure 2:**
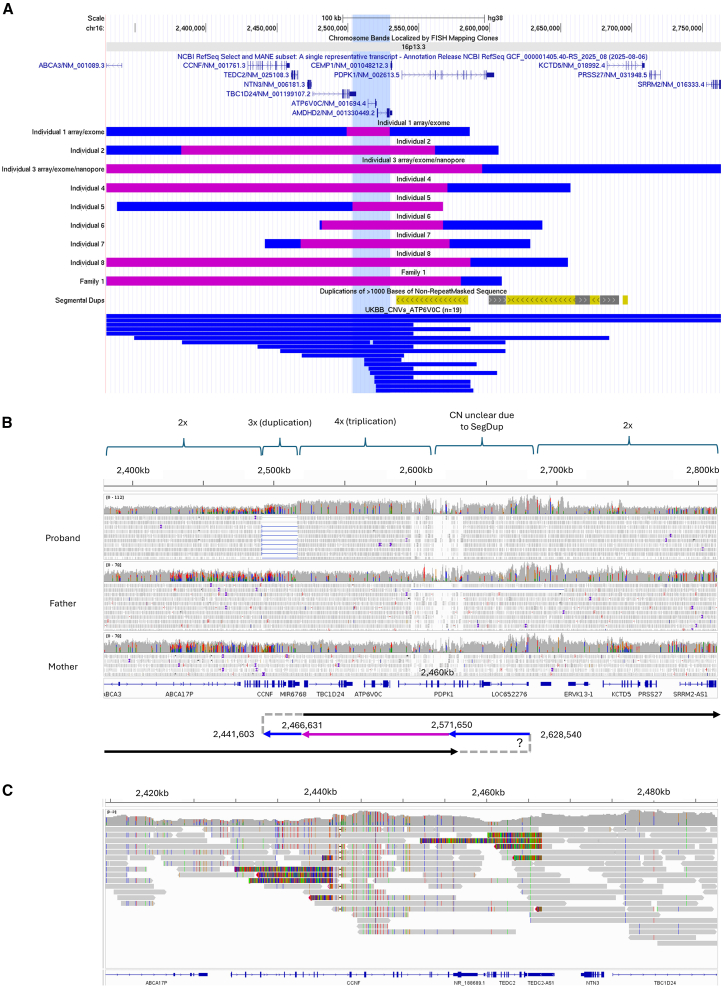
Genetic findings across 11 individuals with overlapping SVs on 16p13.3 (A) Custom UCSC browser session for the region chr16:2329790–2762907 showing relative positions of SVs across individuals 1–8 and family 1. Blue shading denotes duplication, and purple shading represents triplicated segments. For individuals 1 and 3, the array datasets available are of low resolution, so the presence of triplication is confirmed only by exome data and, hence, is approximate. The minimal region of triplication overlap is highlighted in light blue and overlaps a single protein-coding gene in its entirety, *ATP6V0C*. Segmental duplications are shown below the custom SV track and where yellow shading denotes similarity of 98%–99%. The bottom track shows 19 duplications identified in the UK Biobank that overlapped *ATP6V0C*. An interactive version of this image is available at https://genome.ucsc.edu/s/ExeterGenetics/16p13.3-TRIPv9. (B) Short-read alignments shown in IGV that support the presence of the SV in individual 7, chosen as a representative example. The *x* axis coordinates correspond to the same region shown in (A) (coordinates lifted over to hg19). Regions of 3× and 4× are labeled, and split read pairs (blue) are seen on the distal end of the SV, where both reads map to the negative strand and coincide with the stepped increases in coverage. The patchiness seen at the proximal end of the SV is due to reads with low mapping quality, shown in white. The purple/blue arrows in the subway plot denote that the central segment of the SV has been inverted. Other hypothetical conformations are shown in Figure 4. (C) PacBio HiFi data for the same individual confirm the distal ends of the SV. Instead of split read pairs, the junction is visible due to soft-clipped bases present in a subset of reads. Data are shown according to the genome build GRCh38.

Across the cohort, SV sizes ranged from 157 to 918 kb (Tables 1 and S1; Figure 2A). In all 7 families where short-read GS data were available (individuals 2 and 4–8 and family 1), the SV comprised a triplicated segment embedded within a duplication. As a representative example, IGV screenshots showing GS read alignments for individual 7 are shown in Figure 2B. Read alignments for other affected individuals are shown in Figures S2–S7. In all but one case (individual 5; Figure S4), split read pairs at the distal end (both mapping to the negative strand) suggest that the central portion of the rearrangement has been inverted. For individual 5, the duplication-triplication was also present in the unaffected father in a mosaic state (Figure S4), estimated to be heterozygous in 34%–37% of blood cells (Note S3).

Our systematic analysis of SVs in the 100kGP data strongly suggested that the presence of a 4-copy region is critical for the early-onset progressive ataxia phenotype (Note S4), and this was supported by the results for individuals 4–8 and family 1. This prompted closer scrutiny of the existing data for individuals 1–3. The datasets available for individuals 1 and 3 were limited to exome sequencing and aCGH. Lower-resolution aCGH data could not distinguish between duplicated and hypothetically triplicated regions, and data from a ∼60,000-resolution array for individual 3 are shown as a representative example in Figure S8. In contrast, retrospective coverage analysis of the previously uninformative exome data was consistent with segments of triplication embedded within the respective duplications (Figure S9). For individual 2, repeat analysis using a higher-resolution array with ∼1.8 M probes identified a nested region of triplication (Figure S10), undetected by the original SNP microarray. This was subsequently confirmed by short-read GS (Figure S2). In individual 7, multiplex ligation-dependent probe amplification testing prior to 100kGP recruitment yielded fold change (FC) results intermediate between CNs 3 and 4 (Figure S11). These data highlight the difficulties of using non-GS methodologies to distinguish between duplicated and triplicated segments of under 500 kb.

The triplicated segments within the 16p13.3 SVs varied in size from 30 to 811 kb across the 11 individuals reported here (Table 1). The minimal region of triplication overlap was shown to be 26.1 kb and defined by the distal breakpoint in individual 5 and the proximal breakpoint in individual 1 (g.2503226–2529370 [GenBank: NC_000016.10]; Figure 2B; Table S1). A single protein-coding gene, *ATP6V0C* (MIM: 108745), lies fully within this region. *ATP6V0C* is highly expressed in the cerebellum, with a median “transcripts per million” (TPM) of 643.3 (GTEx data, accessed April 24, 2025). Other genes partially within or nearby the shared triplicated region include *TBC1D24* (MIM: 613577), *AMDHD2* (MIM: 620864), and *CEMP1* (MIM: 611113), and the evidence supporting the relative strength of candidacy for each of these is shown in Table S2.

Due to the presence of the 144 kb palindrome-like repeat, precise proximal breakpoint locations remain uncertain in most cases. The exception to this is the multiplex kindred (family 1), where, despite low mapping quality, split read pairs mapping to the positive strand helped resolve the proximal breakpoint junction (Figure S12). We next undertook PacBio HiFi GS for individual 7 using the Revio instrument, seeking to resolve more precisely the structure of these complex palindrome-mediated SVs. Analysis used the HiFi-human-whole-genome sequencing (WGS)-Workflow Description Language (WDL) pipeline (web resources). We obtained a mean coverage of 36× and validated the distal breakpoints that had previously been identified using short reads (Figure 2C). Although SV calling with HiFiCNV (v.1.0.0) and pbsv (v.2.9.0) identified a 120 kb duplication (g.2460001–2580000 [GenBank: NC_000016.10], imprecise) and a 25 kb inversion (g.2441607–2466627 [GenBank: NC_000016.10]) at the distal end, the proximal breakpoints remained algorithmically unresolved. Assigning read alignments into phase groups with HiPhase^13^ and *de novo* assembly with hifiasm^14^ was also unable to resolve the proximal end. However, by analyzing the phase of two *cis*-morphisms that are found 4.2 kb apart in the middle of the highly homologous repeat (Figure S13), we inferred approximate proximal breakpoint positions (Table S1). Similarly, we undertook nanopore sequencing for individual 3 according to the SQK-LSK110 protocol (Note S5) but obtained a mean genome-wide coverage of only ∼5×. Although the distal breakpoint of the SV was identified (Figure S14), there were only four supporting reads, ranging from 849 bp to 35.9 kb in size. Due to higher per-base error rates with nanopore sequencing, *cis*-morphism analysis was not attempted, so the precise variant structure remains unclear.

**Figure 3:**
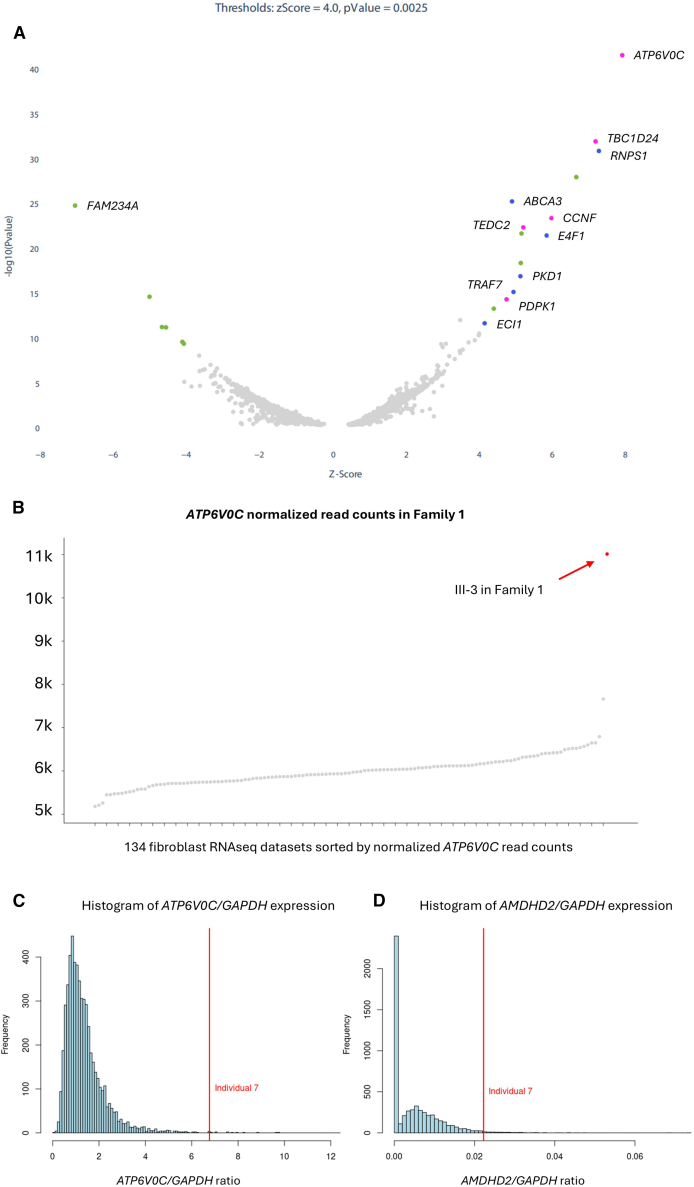
RNA-seq data for two individuals with 16p13.3 rearrangements confirm aberrant gene expression (A) Genome-wide volcano plot showing genes that had significantly aberrant expression (*Z* score < −4 or > 4, *p* < 0.0025) in individual III-3 from family 1. Significantly upregulated genes that lie inside or outside the 16p13.3 duplication/triplication are highlighted in blue/purple or green, respectively. The most significant result was *ATP6V0C*, which lies inside the triplicated segment and has a *Z* score of 7.90. Although *FAM234A* also lies on 16p13.3, it is not in the duplicated region, and the aberrant expression detected was more likely due to a rare intron 1 variant in the 5′ UTR (c.−140+2T>G [GenBank: NM_032039.4]). *Z* scores and *p* values were calculated by comparing results with 133 controls. (B) Normalized read counts for *ATP6V0C* for all 134 fibroblast datasets confirm individual III-3 from family 1 to be the most significant outlier, with a relative fold change of 1.83. Similar RNA-seq results for individual 2 are presented in Figures S18 and S19. (C and D) RNA-seq data from the 100kGP shows individual 7 to be an outlier for (C) *ATP6V0C* expression (4.95× above the mean) and (D) *AMDHD2* expression (4.48× above the mean), following normalization to *GAPDH*, when compared to 5,545 other RNA-seq datasets. Both genes lie within or partially within the triplicated segment that is shared across all 9 families. Data normalized to a second housekeeping gene are shown in Figure S20.

To determine the likely transcriptional effect of these SVs, RNA-seq was undertaken for two affected individuals (III-3 in family 1 and individual 2), with consistent findings across these and a third already available RNA-seq dataset (described below). Fibroblast-derived RNA was utilized for family 1, III-3. Data analysis employed an adapted version of the OUTRIDER method described previously^15^ and a transcript set based on RefSeq MANE v.1.3 (GRCh38). *Z* scores, FCs, and *p* values were calculated by comparing results with 133 control individuals, all unrelated patients for whom RNA-seq-based diagnostic testing had been requested at the Erasmus MC Clinical Genetics department. *ATP6V0C* was the most significantly dysregulated gene (Figures 3A and 3B), with a *Z* score of 7.90, an adjusted *p* value of 1.42 × 10^−13^, and a FC of 1.83. Of the 15 upregulated genes with *Z* scores > 4 and *p* < 0.0025, 11 were localized within the 16p13.3 duplication/triplication (Table S3; Figure S15). RNA-seq data also identified a fusion transcript involving *PKD1* (MIM: 601313) and *ABCA3* (MIM: 601615) (Figures S16 and S17). However, given the variable distal breakpoints of overlapping SVs in this cohort, the fusion transcript’s significance is unclear.

RNA-seq analysis for individual 2 used cultured lymphoblast-derived RNA. OUTRIDER analysis was performed across 114 samples sent in for RNA-seq-based diagnostics to identify genes with significant expression differences compared with the rest of the cohort. While the initial analysis used basic Gencode annotations and yielded non-significant results for *ATP6V0C* (FC = 2.10, *Z* score = 2.34), the results were confounded by Gencode annotations for fusion genes (Gencode: ENSG00000260272 and ENSG00000259784) that overlap the primary transcript for *ATP6V0C* (Gencode: ENST00000330398). Repeating this analysis using just the “MANE select” annotations, *ATP6V0C* became the most significantly upregulated gene (FC = 1.95, *Z* score = 6.86; Figures S18 and S19). Overall, 15/16 of the upregulated genes are from the inverted duplication-triplication region (Table S4). A split read pair with similar genomic coordinates to those seen in the GS data confirmed the distal breakpoint (Figure S2).

RNA-seq data were also available for individual 7 as part of the Genomics England transcriptomics study.^16^ In brief, blood-derived RNA for 5,546 probands was collected using archived PaxGene tubes taken at recruitment to the 100kGP. RNA samples were depleted for rRNA/globin and sequenced using 100 bp paired-end reads. Alignment and transcript quantification were performed using Illumina’s DRAGEN pipeline (v.3.8.4). Compared with the other 5,545 RNA-seq datasets and normalized to *ACTB* or *GAPDH*, the relative expression in this individual was 4.16–4.95× above the mean for *ATP6V0C* and 3.48–4.48× above the mean for *AMDHD2* (Figures 3C, 3D, and S20). Accurate measurement of *CEMP1* expression was not possible with short-read RNA-seq, as the gene is embedded within the 3′ UTR of *AMDHD2*.

To investigate the incidence of similar 16q13.3 rearrangements in the general population, we interrogated UK Biobank (UKB) data, which comprises ∼500,000 UK individuals between the ages of 40 and 69 years at recruitment. Of these, 490,640 participants underwent GS, as previously described.^17^ CNVs were called using the DRAGEN v.3.7.8 CNV module, and data were searched using a custom RStudio script and a 10 kb size threshold. Nineteen individuals were identified harboring duplications of between 25.4 kb and 2.94 Mb that overlap *ATP6V0C* (Figure 2A). No triplications (i.e., a CN of 4) were identified, and segment CN ratios were 1.27–1.56. All of these individuals were healthy enough to be recruited to the UKB as adults. By reviewing hospital episode statistics, general practitioner (GP) records, and other self-reported clinical information, only one individual was identified who could plausibly be consistent with a milder form of the 16p13.3-triplication-linked condition, presenting with idiopathic familial dystonia and mild cognitive disorder (ICD-10 codes G24.1 and F06.7).

The clinical and genomic data described here define palindrome-mediated 16p13.3 triplication as the cause of a recognizable neurodegenerative disorder characterized by progressive ataxia and intellectual disability, with additional neurological features in many individuals. Cognitive impairment from childhood was universal and progressive in 80% of affected individuals. Notably, individual 1 developed severe, progressive dementia, raising the possibility of similar trajectories in other adult patients. Ataxia typically emerged during childhood or adolescence and progressed gradually. Peripheral neuropathy, spinal anomalies, and cerebellar signs, such as dysarthria and nystagmus, were frequent, though variably present. While some individuals had dystonia or spasticity, seizures were notably absent. Importantly, all patients shared a strikingly consistent MRI pattern, with cerebellar and caudate atrophy and basal ganglia signal abnormalities, supporting a shared, progressive neurodegenerative trajectory. This constellation of clinical and radiological features should prompt consideration of this disorder in undiagnosed individuals.

This condition is distinct among neurodegenerative ataxic disorders. While several conditions combine progressive ataxia with predominantly sensory axonal peripheral neuropathy (e.g., Friedreich ataxia [MIM: 229300],^18^ ataxia with vitamin E deficiency [MIM: 277460], and CANVAS [MIM: 614575]^19^^,^^20^), none are typically accompanied by progressive cognitive decline. Progressive intellectual impairment does occur in multisystemic conditions such as mitochondrial diseases and Refsum disease (MIM: 266500), but these typically also manifest outside the nervous system (e.g., as retinitis pigmentosa or cardiac impairment).

Although recent advances have improved our understanding of the genetic basis of rare hereditary ataxias and neurodegenerative disorders, current diagnostic yields remain below 50%,^21^ suggesting that additional causative mechanisms remain to be identified. SVs, including copy gains, represent a class of genomic alterations that is technically challenging to detect and fully characterize and may account for a subset of undiagnosed individuals. Although CN gains have been found to cause complex neurodegenerative conditions, pathogenic triplications are relatively sparse in the literature. In many cases where triplication is a disease mechanism (*PLP1*, Pelizaeus-Merzbacher disease^22^; *SNCA*, Parkinson disease [MIM: 605543]; 17q22, retinitis pigmentosa [MIM: 600852]^23^; and *APP*, Alzheimer disease [MIM: 104300]^24^), the phenotype is more severe than the equivalent condition caused by duplication of the same locus. The neurodegenerative disorder described here contrasts with these, as there does not appear to be a consistent, milder ataxia/neurological phenotype in duplication carriers, supporting a higher dosage threshold for phenotypic expression. Based on this model, one would hypothesize that a relative CN of 4 due to a homozygous duplication would result in the same phenotype as a single triplicated allele. Alternatively, the mechanism may specifically depend on triplication.

**Figure 4:**
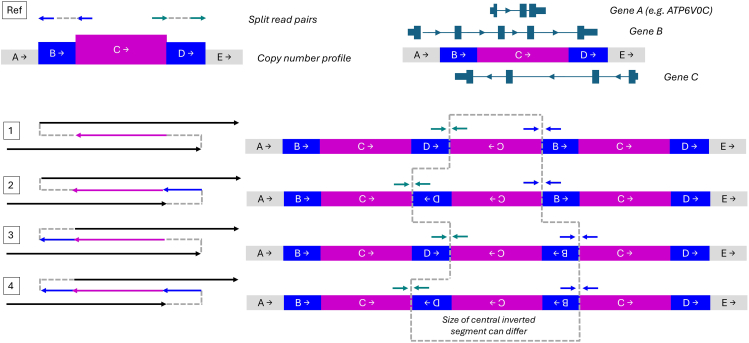
Schematic diagram showing the split-read pairs and copy number profile changes that are associated with inverted duplication-triplications Although these types of rearrangements appear complex and can be called as 2 inversions, 2 duplications, and a central triplication, they involve just two breakpoints. The subway diagram highlights that there are typically 4 possible configurations that can all explain the short-read data. The size of the central inverted segment depends on which of these structures is true, and this can impact the interpretation. For instance, in this hypothetical example, the rearranged chromosome contains 3 copies of gene A (e.g., *ATP6V0C* in the present series of structural variants), irrespective of which precise structure is involved. In contrast, gene B is disrupted in solutions 2 and 4, while gene C would be disrupted in solutions 3 and 4. Ultra-long-read sequencing data or optical genome-mapping technologies where reads/labeled molecules span both breakpoint junctions can help distinguish which of the 4 possible solutions is right.

Genomic palindromes can form hairpin or cruciform-type structures that promote double-strand breaks and lead to the formation of complex SVs. For instance, several inter/intra-chromosomal insertions into a 180 bp palindrome have been described ∼80 kb downstream of *SOX3* (MIM: 313430). Depending on which sequences have been inserted, these can result in a wide range of phenotypes.^25^^,^^26^^,^^27^^,^^28^ It is estimated that 718 palindromes > 200 bp are spread across the human genome.^29^ Future studies should investigate these genomic loci for complex SVs that may also represent novel genomic disorders. There is likely considerable background of benign structural variation in the general population at the 16p13.3 locus due to the palindrome, and we note that GRCh38 and the CHM13 v.2.0 reference genomes differ substantially (Figure S21). Furthermore, the overall structure of inverted duplication-triplications is often ambiguous unless much longer sequencing reads or optical mapping data are available that span the central inverted segment (Figure 4). Resolving inverted duplication-triplication structures may become a key use case for long-read sequencing technologies as effective read lengths increase, and this locus, with its 33 kb segments of 99.8% sequence identity (Figure S22), is particularly challenging.

Using 100kGP data, we previously identified 3 individuals with intellectual disability and genetic haploinsufficiency of serine/arginine repetitive matrix 2 (*SRRM2*; MIM: 606032) resulting from complex deletion-inversions, with distal breakpoints clustered within the same 16p13.3 palindrome.^30^ An additional *SRRM2* deletion-inversion identified from the GMS further supports the recurrent nature of complex SVs at this locus (Figure S23). Heterozygous loss-of-function variants in *SRRM2* (HGNC: 16639), a gene encoding a splicing factor, had previously been shown to result in autosomal-dominant intellectual developmental disorder type 72 (MIM: 620439).^31^^,^^32^ The findings described here support our hypothesis that the symmetry of this 144 kb palindromic repeat would make the formation of complex SVs equally likely in both directions. In contrast to the proximal deletion-inversion events, where the molecular mechanism is *SRRM2* haploinsufficiency, RNA-seq has demonstrated a robust increase in expression for several genes within these more distal inverted duplication-triplications. Interpretation is limited by the fact that RNA data were available for only 3 of 11 individuals, from three different laboratories, and using non-disease-relevant tissues. Future studies assessing RNA and protein levels in disease-relevant cell types derived from postmortem tissues or differentiated induced pluripotent stem cells (iPSCs) would be valuable. It remains to be seen whether further instances of 16p13.3 triplication could be uncovered in genetically unsolved individuals with ataxia using an RNA-seq-first approach.

The identification of critical dosage-sensitive genes within disease-associated CNVs is challenging. Although several mechanistic questions remain, the most compelling candidate gene within the 16p13.3 SV interval is *ATP6V0C.* Deleterious variants in this gene have previously been linked to neurodevelopmental abnormalities, with epilepsy reported in several cases.^33^^,^^34^ Microdeletions including this gene have also been implicated in neurodevelopmental disorders, with some sharing proximal breakpoints near the palindromic repeat.^35^ *ATP6V0C* encodes the C subunit of the membrane-bound integral domain of a vacuolar proton pump enzyme complex, which acidifies organelles and establishes a proton gradient critical for several cellular processes. Pathogenic variants found in individuals with neurodevelopmental abnormalities were shown to impair V-ATPase function.^36^ In family 1 and individual 2, RNA-seq indicated that the most significant gene expression changes occurred in *ATP6V0C*. We therefore speculate that the triplications described here may disturb the correct stoichiometry of subunits required for efficient assembly of the vacuolar H^+^-ATPase. While a peripheral domain is involved with ATP hydrolysis, the integral membrane V0 domain mediates proton pumping via a rotary mechanism. This V0 domain contains a c-ring structure that rotates and is made up of 9 subunits encoded by *ATP6V0C* and just one encoded by *ATP6V0B*.^36^ Future studies assessing how enzyme assembly is altered in cell lines from affected individuals and whether this leads to abnormal proton gradients being established would provide valuable pathomechanistic insights.

Genes encoding other subunits of this large enzyme complex have also been linked to neurological disorders.^36^ Notably, *de novo* variants in *ATP6V1A*, which encodes the A subunit, cause developmental encephalopathy with epilepsy.^37^ More recently, variants in *ATP6V0A1* (which encodes the brain-enriched isoform of the a subunit in the V0 domain) have been shown to cause progressive myoclonus epilepsy and developmental and epileptic encephalopathy. Of relevance to the present study, 8 of 13 patients with *ATP6V0A1* variants presented with ataxia, and 25% had cerebellar atrophy.^38^^,^^39^

Another gene that lies within the region of triplication in all but one case is *CEMP1,* a single-exon gene embedded within the final exon of *AMDHD2*. This gene regulates the deposition of cementum that, together with the periodontal ligament, helps anchor teeth to the jawbone^40^ and thus is not a strong functional candidate. Another candidate gene that lies proximal to *ATP6V0C*/*CEMP1* on 16p13.3 and is likely impacted by this SV is *PDPK1* (MIM: 605213). Based on an analysis of large rare CNVs in almost 1 M individuals,^41^ *PDPK1* is predicted to be triplosensitive (pTriplo = 0.99). This gene encodes 3-phosphoinositide-dependent protein kinase 1 and is crucial for mammalian brain development. Mice with conditional knockout of the orthologous gene exhibit decreased cerebellar size and ataxia-like behavior, suggesting that *pdpk1* may be critical for motor balance and coordination.^42^ GTEx data indicate that *PDPK1* has high relative expression in the cerebellum (a median TPM of 33.73). Although the gene does not fall within the minimal region of overlap, it must be noted that proximal breakpoints were imprecise due to the 144 kb palindromic repeat, and so the regions of triplication could potentially extend to include this gene in some cases. Even with the ∼20 kb PacBio reads available for individual 7, proximal breakpoints of the rearrangement were unclear, and we hypothesized that they are situated between two *cis*-morphisms. Imprecise SV characterization is also pertinent to individuals 1 and 3 due to the low resolution of aCGH data, the low coverage of nanopore data, and the inability to obtain fresh samples, and the presence of a triplication was inferred from exome sequencing data.

While a simple dosage threshold model is an attractive option, we cannot rule out the occurrence of aberrant topologically associating domains (TADs) leading to ectopic gene expression. For other genomic disorders linked to specific SVs, 3D chromatin conformation using Hi-C and related methods has demonstrated how the altered distribution of TADs can impact gene expression.^43^^,^^44^^,^^45^ One example is split-hand/foot malformation type 3 (MIM: 246560), where ∼0.5 Mb duplications on 10q24 show a high degree of clustering of both start and endpoints.^46^ This similarity is due to the precise enhancer repositioning, which mouse studies show is required to lead to the ectopic expression of *Lbx1* and *Btrc*.^43^ The heterogeneity of sizes for the 16p13.3 SVs described here argues against a similar mechanism. However, future experimental studies using Hi-C should be complemented with deep learning computational methods^47^ to confirm whether these complex genomic rearrangements affect chromatin structure and contribute to pathology.

We note that a complex rearrangement comprising 5 copies of *ATP6V0C* was recently described in a single individual presenting with ataxia.^48^ The age of onset in this patient appears to have been earlier than in the 11 cases described in the present study (Table 1), with frequent falls and poor motor skills already noted by the age of 4. Additional features included amblyopia, hyperopia, dysarthric speech, coughing with liquid intake, excessive drooling, a progressive decrease in IQ, and cerebellar atrophy, which was first noted at 11 years (K. Hamanaka, personal communication). Future studies showing a correlation between higher CN states and disease severity would be of benefit for conclusively elucidating the pathomechanistic basis and clinical spectrum of this neurodegenerative disorder.

Together, our studies define a childhood-onset complex neurological disorder caused by overlapping 16p13.3 duplication-triplications and characterized by neurodegenerative features, including progressive ataxia, cognitive decline, and neuropathy, and a distinctive neuroradiological phenotype. Proximal breakpoints clustered within the 16p13.3 palindrome, making this the second complex genomic disorder linked to this mutational hotspot. Refinement of the spectrum of complex SVs that can lead to this disorder, combined with analyses of the consequences of increased *ATP6V0C* dosage on vacuolar H^+^-ATPase formation and function, will be crucial to determining the underlying cause of disease and thus potential therapeutic interventions for this devastating disorder.

## Data and code availability

Data for individuals 1–4 are available via the DECIPHER database (www.deciphergenomics.org). For individuals 4–8, GS data (including structural VCFs and gene-level SV reports) and transcriptomic data (for individual 7 only) are available in the NGRL v.5.1, Genomics England. Information about the NGRL can be found at https://doi.org/10.6084/m9.figshare.4530893.v7, and details of how researchers can apply for access can be found at www.genomicsengland.co.uk/join-us. For family 1, genome and RNA-seq data are stored in a repository at the Clinical Genetics Department of the Erasmus MC. The long-read GS data for individuals 3 and 7 and the exome sequencing data for individuals 1 and 3 are stored by the Exeter Genomics Laboratory. The consent agreements for these datasets only permit sharing anonymized data for affected individuals, and therefore, we are unable to share BAM or VCF files. The code to query SVs in the NGRL is available in the Genomics England Research Environment User Guide, found at https://re-docs.genomicsengland.co.uk/structural_variant. The other code used in this study has already been published.

## Acknowledgments

First and foremost, we are grateful to the families in this study. We thank Jing Yu for sharing SVRare reports and Mark Nellist for useful discussions. This research was funded by the NHS Rare and Inherited Disease Genomic Network of Excellence and the 10.13039/501100000272National Institute for Health and Care Research (NIHR) Exeter Biomedical Research Centre (BRC). The views expressed are those of the authors and not necessarily those of the NIHR or the Department of Health and Social Care. This research was made possible through access to data in the NGRL, which is managed by Genomics England Limited (a wholly owned company of the Department of Health and Social Care). The NGRL is funded by the NIHR and NHS England. The Wellcome Trust, 10.13039/501100000289Cancer Research UK, and the 10.13039/501100000265Medical Research Council funded research infrastructure. This study makes use of data generated by the DECIPHER community. A full list of centers that contributed to the generation of the data is available from https://deciphergenomics.org/about/stats. DECIPHER is hosted by EMBL-EBI. DECIPHER project funding was provided by the Wellcome Trust (WT223718/Z/21/Z). This research was conducted using the UKB Resource under application number 103356 and used data provided by patients collected by the NHS as part of their care and support. UKB protocols were approved by the National Research Ethics Service Committee. The Barakat lab was supported by the 10.13039/501100003246Netherlands Organisation for Scientific Research (ZonMw Vidi, grant 09150172110002). The Rare Disease Flagship acknowledges financial support from the 10.13039/100014607Royal Children's Hospital Foundation.

## Author contributions

E.L.B., J.R., and A.H.C. conceived and supervised the study. L.M. analyzed the radiological images. E.L.B., A.H.C., R.S., T.S.B., S.M.W., and S.L.H. coordinated the collaborations. A.H.N., A.K., A.S., A.W., B.B.-P., C.R., E.L.B., E.T., F.H.S., G.K.T., J.F., J.R., J.t.W.N., K.J., K.V., M.G.d.S., M.H., M.S., M.W., P.G., and S.M.W. recruited the patients and/or assessed the clinical information. K.M.B., L.J.M., and T.S.H. provided bioinformatic support. D.I.F. and E.E.J. analyzed the array data. A.P. and A.R.J. supervised the GS analysis. R.A.B. and N.U. performed the genetic studies. A.T.P., F.S., J.F., J.L., J.S.L., J.v.G., M.N.W., R.S., S.W., and T.J.v.H. analyzed the genomic and/or transcriptomic datasets. A.T.P. and J.F. wrote the initial draft of the manuscript.

## Declaration of interests

S.W. is an employee at Genomics England.

## Declaration of generative AI and AI-assisted technologies in the writing process

During the preparation of this manuscript, the authors used ChatGPT (OpenAI) to assist with improving the grammar and clarity of some sentences. The authors subsequently reviewed and edited all content to ensure accuracy and take full responsibility for the final manuscript.

## Web resources

DECIPHER database, www.deciphergenomics.org
Gencode, https://www.gencodegenes.org
GeneMatcher, https://genematcher.org
GTEx, https://gtexportal.org
hifiasm, https://github.com/chhylp123/hifiasm
HiFi-human-WGS-WDL pipeline, https://github.com/PacificBiosciences/HiFi-human-WGS-WDL
HGNC, https://www.genenames.org
NCBI BLAST tool, https://blast.ncbi.nlm.nih.gov
OMIM, https://www.omim.org

## Supplemental information

Document S1. Figures S1–S24, Tables S1–S4, and Notes S1–S5

Document S2. Article plus supplemental information
