# Supplementary material for: Palindrome-mediated 16p13.3 triplications cause a recognizable neurodegenerative disorder with ataxia
Source: Am J Hum Genet. 2025 Dec 4;113(1):221–33. doi: 10.1016/j.ajhg.2025.11.011 (PMC12824621; doi:10.1016/j.ajhg.2025.11.011)
Supplement: Document S1. Figures S1–S24, Tables S1–S4, and Notes S1–S5 [file mmc1.pdf]

The American Journal of Human Genetics, Volume *113*

Supplemental information

Palindrome-mediated 16p13.3 triplications cause a recognizable neurodegenerative disorder with ataxia

James Fasham, Julia Rankin, Rachel Schot, Susan M. White, Katrina M. Bell, Matthew N. Wakeling, Lucy J. Mallin, Alex Shah, Michelle G. de Silva, David I. Francis, Maie Walsh, Emily E. Jones, Kayal Vijayakumar, Katie Johnson, Francis H. Sansbury, Johann te Water Naudé, Paola Giunti, Marios Hadjivassiliou, Andrea H. Nemeth, George K. Tofaris, Carlo Rinaldi, Benito Banos-Pinero, Marianna Selikhva, Nishanka Ubeyratna, Anneke Kievit, Frank Sleutels, Joey van Giessen, Tahsin Stefan Barakat, Timothy S. Hall, Alan Whone, Eleanor Thomas, Joseph S. Leslie, Rosemary A. Bamford, Aaron R. Jeffries, Jenny Lord, Susan Walker, Tjakko J. van Ham, Sue L. Hill, Lucy McGavin, Andrew Parrish, Andrew H. Crosby, Emma L. Baple, and Alistair T. Pagnamenta

Note S1: Clinical case reports for affected individuals.

Individual 1

Individual 1 was the first child of unrelated parents. She had two healthy younger siblings and there was no family history of note. She was born at 34 weeks gestation and there were no reported complications during pregnancy or in the neonatal period. Early development was unremarkable, but her parents described her as a clumsy child. She attended a mainstream school with additional educational support in some areas (and a statement of special educational needs) but could read well. Slurred speech and walking difficulty were noted at age 10 and these progressed slowly over a number of years. Aged 14 she was able to walk half a mile but was unsteady.

Assessment by a pediatric neurologist at age 14 revealed truncal and limb ataxia, dysarthria, bilateral *pes cavus* and absent lower limb reflexes with equivocal plantar responses. Joint position and vibration sense were normal. Nystagmus and scoliosis were absent and there was no muscle weakness. Height was on the 75th centile.

Spinal MRI aged 13 was normal but brain MRI aged 16 showed marked cerebellar atrophy. At age 14 nerve conduction studies showed evidence of an axonal sensory neuropathy and at age 15 she underwent ambulatory EEG to investigate two possible seizures - daytime EEG was normal and nocturnal EEG showed possible predisposition to seizures but no seizure activity. Sodium Valproate treatment was started at this time and continued into adulthood, but no similar episodes were reported. The ataxia progressed and she started using a wheelchair in her 20s. Progressive dementia developed from her late 20s – at age 28 she could still read a book and play card games but when assessed at age 37 she had very limited understanding, aggressive behavior and was doubly incontinent without awareness of this. Her carers reported frequent 'blank' episodes lasting 10-15 minutes during which she was awake but would not respond in her usual manner. These were not investigated further.

On examination aged 37 she had coarse facial hair on her chin (present for at least 9 years), she was not dysmorphic, she was non-ambulant and in a wheelchair. Dysarthria and echolalia were noted, she had a full range of eye movements with no nystagmus but upgaze could not be elicited. Reflexes were absent and she had bilateral *pes cavus* with clawed toes. There was no evidence of muscle weakness, but cooperation with the examination was limited. Her mobility became progressively more severely impaired; she developed swallowing difficulties and died aged 42 years. MRI brain aged 35 showed marked parietal and cerebellar volume loss with caudate nuclei and putamina that were small with high T2 signal. Genetic testing for Friedreich Ataxia, Ataxia Telangiectasia, vitamin E deficiency and abetaliproteinaemia, SCA1 (*ATXN1*), SCA2 (*ATXN2*), SCA3 (*ATXN3*), SCA6 (*CACNA1A*), SCA7 (*ATXN7*), SCA17 (*TBP*) and Huntington's (*HTT*) were unremarkable. Gene agnostic trio exome analysis (Agilent SureSelect Human All Exon Kit v6 and sequencing on Illumina NextSeq) revealed no likely genetic diagnosis (a *de novo POLR2A* variant of uncertain significance was noted but thought unlikely to be deleterious and unlikely to explain her phenotype). Chromosome analysis revealed a normal female karyotype and plasma amino acids were normal apart from mildly elevated alanine which was not thought to be significant. Array CGH revealed a *de novo* 448kb duplication of chromosome 16p13.3.

Individual 2

Individual 2 is a 28 year old woman. She was born after an uneventful pregnancy to parents of Australia-European genetic ancestry. She has three older healthy siblings. Early developmental

milestones were normal but she was noted to have learning difficulties around age five years and was diagnosed with a mild intellectual disability. She completed her education with learning support. She developed a thoracolumbar scoliosis and was noted to have coordination difficulties, which were progressive.

An MRI of the brain aged 18 years showed cerebellar atrophy in cerebellar hemispheres and superior vermis. Volume loss was noted in the caudate nuclei and putamen bilaterally with associated T2/FLAIR hyperintensity. These findings were stable on repeat imaging at age 23. Growth at 19 years of age showed height on the 29th centile and weight on the 28th centile. No dysmorphic features were noted. She had an ataxic gait, with normal tone and power. Reflexes were difficult to elicit.

On reassessment at age 25 years, her ataxia had progressed. She was unsteady on her feet and had difficulties with fine motor tasks such as writing. Cognitively she continued to gain new skills, and her parents reported improved working memory. She did have fatigue in the afternoons requiring additional rest. She had some difficulty swallowing liquids. She was working as a volunteer and attending art classes. On examination, she had dysarthria, dysmetria and dysdiadochokinesis. She had an action tremor.

Individual 3

The female proband was the second child of three born to non-consanguineous parents of Sri Lankan genetic ancestry. The pregnancy was unremarkable, and she was born at term in good condition by *caesarean* section due to breech presentation with a birth weight of 2.4 kg. There were no neonatal concerns other than mild jaundice and there was normal attainment of early developmental milestones.

She started mainstream school in the UK at the age of 5. It was noted that she had some mild difficulties with learning and memory at this stage. At the age of 9 years, she started to experience recurrent trips and falls in addition to problems with fine motor skills at school. After physiotherapy input in the *community,* she was referred for further assessment. MRI brain showed cerebellar atrophy and volume loss. Electrophysiology studies showed chronic partial denervation in the tibialis anterior muscles. There was evidence of cerebellar ataxia (dysarthria and intention tremor). She also had bilateral *cavovarus* foot deformity and progressive scoliosis and absent reflexes.

Over a period of six years there was gradual deterioration in mobility resulting in use of a wheelchair. At the age of 16 years there was a significant deterioration in cognition and regression with severe dysarthria, dysphagia to solid food, weight loss and incontinence.

Individual 4

The patient is the second child of unrelated, healthy parents of British genetic ancestry. The reported family history is notable for neurodevelopmental conditions: her older sister has a diagnosis of ADHD (attention deficit hyperactivity disorder); her maternal cousin (daughter of her mother's sister) has polymicrogyria and learning difficulties; her maternal cousin (son of the same sister) has ADHD; and three of her paternal aunt's four sons all have learning difficulties.

She was born at term following an uncomplicated pregnancy and delivery, with a birth weight of 4.42 kg. She walked at 18 months and had no speech until the age of 2, after which she received speech and language therapy. At age 6-7 years, parental concerns prompted a referral to community paediatrics. At age 10, she exhibited poor attention span, clumsiness, and significant coordination difficulties. She required prompting to dress and assistance with showering and had no awareness of danger. Despite these challenges, she interacted well with peers and formed friendships. She

received diagnoses of ADHD and learning difficulties, both considered mild-to-moderate in severity. At age 13, she transitioned from mainstream education to a special needs school following a formal SEN (special educational needs) statement.

Although parents reported clumsiness, clinical assessments at ages 11 and 14 found no evidence of ataxia or cerebellar signs. By age 15, however, neurological symptoms had emerged, including frequent falls, mild oropharyngeal dysphagia, positive Romberg's sign, absent reflexes, and gaze-evoked nystagmus. Speech remained fluent. Examination at age 14 showed left-sided scoliosis (31°). There was no clinical evidence of peripheral neuropathy. Additional findings included bilateral fifth finger clinodactyly, mild constipation, and gastro-oesophageal reflux disease. Examination at age 20 showed mild heel-toe ataxia, mild bilateral nystagmus at extremes of gaze, mild bilateral intention tremor and mild slurred speech.

Growth parameters showed height consistently between the 50th and 75th centiles, and weight around the 75th centile. Occipitofrontal circumference (OFC) was on the 96th and 99.8th centiles on consecutive measurements. MRI scan aged 20 showed significant progression of cerebellar atrophy compared to the scan aged 20, as well as cerebellar volume loss.

Array CGH identified a *de novo* apparent duplication of 16p13. Trio genome sequencing, performed as part of the 100,000 genomes project (100kGP), confirmed this to be a *de novo* duplication-triplication of 16p13.3. The 100kGP also identified compound heterozygote *DHCR7* variants (P and VUS; NM_001360.3:c.452G>A, p.Trp151Ter and c.349T>A, p.Phe117Ile), but the patient does not have the associated phenotype of Smith-Lemli-Opitz syndrome. 7-DHC (7-dehydrocholesterol) levels were normal.

Individual 5

This 40-year-old woman, of Pakistani heritage, is the daughter of consanguineous parents and has three clinically unaffected brothers. She presents with cognitive impairment and progressive ataxia accompanied by dysarthria, dysphagia, dysdiadochokinesis, apraxia, and impaired distal proprioception. Eye movement examination revealed slow and hypometric saccades. There was mild sensory neuropathy and progressive scoliosis. Additional features include dystonia, recurrent cystitis, cataracts, cortical visual impairment, thyroid cancer, lymphadenopathy, and iron deficiency anemia. Trio genome sequencing, undertaken as part of the 100kGP, identified a duplication-triplication involving chromosome 16p13.3. This rearrangement was inherited from her clinically unaffected father, who is mosaic for the same variant.

Individual 6

This 65-year-old man is the son of non-consanguineous parents of British genetic ancestry and has a clinically unaffected sister. He first presented at age 39 with ataxia. Clinical history suggested that gait ataxia had been present for at least 7 years prior to presentation. He had normal developmental milestones and was still working as a gardener. Neurological examination revealed dysarthria and gaze evoked horizontal nystagmus. He had pale optic discs but normal visual acuity. Tone and power were normal but he was areflexic. He had bilateral *pes cavus* and blunting of vibration sensation. Neurophysiology showed a pure sensory neuropathy. Imaging showed cerebellar atrophy. Over time he developed significant cognitive and behavioral problems and his vision deteriorated. At the most recent assessment he was unable to mobilize outside of bed and exhibited features of advanced dementia. Trio genome sequencing, conducted through the 100kGP, identified a duplication-triplication involving chromosome 16p13.3.

Individual 7

This 36 year old woman was reported to be very clumsy as a child, but came to medical attention at age 20 for deteriorating balance which deteriorated over the next few years. She was noted at age 23 to have ataxia (unable to walk heel toe), dystonia affecting her neck but also more generalized on the left, and behavioral problems. There was no fundal pallor at that time. She had symptoms of autonomic disturbance with 'fainting' episodes, sweating and constipation. She had a postural drop of 30mmHg. She had numerous investigations, including a muscle biopsy which was suggestive of denervation. By age 29 she was fully dependent on others for care, and could only walk a few steps with support. Numerous genetic tests were negative at that time. Currently she is wheelchair bound due to ataxia, continues to have severe dystonia and has worsening behavioral and cognitive problems. Trio genome sequencing, undertaken as part of the 100kGP, identified a *de novo* duplication-triplication involving chromosome 16p13.

Individual 8

This 40-year-old man is the son of non-consanguineous parents of British genetic ancestry, with no relevant family history. Early development and cognition were normal. He completed formal education with good results, obtaining nine GCSEs. Scoliosis was noted during childhood. At age 12, he developed progressive ataxia that led to recurrent falls. This was accompanied by dysarthria, abnormal saccadic eye movements, absent reflexes on neurological examination and EMG -findings of subclinical sensory axonal neuropathy. The disease progressed slowly over time. By age 36, he required full-time use of a wheelchair and was no longer able to walk unassisted, even with walking aids. Following school, he experienced cognitive decline, including loss of numeracy skills, memory impairment, and an inability to write. His current mode of communication is limited to slurred, hypophonic single-word responses. Additional features include increased somnolence, hypersalivation, reduced dexterity, hypokinesia, and bradykinesia.

Neurological examination revealed intentional tremor on finger–nose testing, head drop, ptosis, impaired postural reflexes, and dystonic finger posturing. Laterocollis, hypometric saccades, broken pursuits, areflexia, *pes cavus*, and mild sensory neuropathy were also present. A left plantar extensor response was noted. Muscle tone remained normal, and muscle strength was preserved. There was no clinical evidence of optic atrophy. A dopamine transporter (DAT) scan performed in 2016 was positive. A trial of Madopar led to worsening paranoia and hallucinations, and he has since been maintained on neuroleptics for chronic psychosis. Extensive investigations, including copper studies, mitochondrial cytopathy screening, respiratory chain enzyme analysis, and genetic testing of the genes *POLG*, *MELAS*, *MERRF* and for common forms of spastic ataxia and spinocerebellar ataxia (SCA1, 2, 3, 6, 7, and 17), were uninformative. No mitochondrial DNA rearrangements or *FXN* GAA expansions were detected. Trio genome sequencing with a virtual panel for adult-onset neurodegenerative disorders also identified no pathogenic variants. Muscle biopsy showed non-specific muscle fibre shrinkage. Reanalysis of trio genome sequencing data through the NHS Genomic Medicine Service subsequently identified a duplication-triplication involving chromosome 16p13.3.

Family 1

II-2

This woman is the mother of two affected children (III-3 and III-4). Her own mother was deceased at the time of diagnosis, and her father has a normal neurological examination and does not carry the 16p13.3 duplication. Her clinically unaffected sisters have not undergone genetic testing for the

16p13.3 copy number variant. Early developmental milestones (walking and speech) were reported to be normal. She attended mainstream school and completed lower vocational education without obtaining a diploma. Neurological symptoms began at age 9, with ataxia, dysarthria, saccadic eye movements, absent reflexes, and intention tremor. At diagnosis (age 46), additional findings included axonal sensory neuropathy, prominent kyphosis, and optic atrophy. Over time, she developed joint contractures, progressive visual loss due to optic atrophy, and eventually unable to leave her bed became unresponsive prior to her death at age 56. An EEG at age 14 showed diffuse encephalopathy. Extensive investigations, including an ataxia gene panel (NGS/WES), metabolic screening, muscle biopsy, mitochondrial analysis, and *FMR1* repeat testing, were all uninformative. SNP array identified a duplication of 16p13.3, later clarified as a duplication-triplication using genome sequencing. This rearrangement was not paternally inherited. No maternal sample was available.

III-3

This is the affected son of II-2 and full brother to III-4. His parents are non-consanguineous; his father has mild intellectual disability. At birth, he required one day in an incubator due to aspiration of amniotic fluid. No other antenatal or perinatal concerns were noted. Motor milestones were delayed, with walking achieved at 26 months. He began in mainstream education but transferred to special education at age 9. Neurological symptoms began around age 12, including ataxia with dysarthria, saccadic eye movements, absent reflexes, and axonal sensory neuropathy. These signs progressed slowly. As of age 36, he is a full-time wheelchair user. A maternally inherited 16p13.3 duplication was identified on SNP array, and subsequent testing in other family members confirmed this to be a duplication-triplication.

III-4

This is the daughter of II-2 and full sister to III-3. Her parents are non-consanguineous; her father has mild intellectual disability. There were no concerns during pregnancy or birth. Development was delayed, and she began education in a specialist setting from age 5. She was later diagnosed with ADHD. At age 11, her total IQ was 65; by age 19, it had declined to 55. Her age at first walking and speech acquisition is unknown. At age 8, she developed ataxia with dysarthria, saccadic eye movements, absent reflexes, and intention tremor. Axonal sensory neuropathy was noted by age 9. These symptoms have progressed slowly, and she now predominantly requires a wheelchair to mobilize at age 27. Her height is 180 cm. An EEG at age 7 was normal. Metabolic and mitochondrial investigations were uninformative. SNP array identified a maternally inherited 16p13.3 duplication, later shown to be a duplication-triplication by genome sequencing. A healthy full brother of III-3 and III-4 has a normal neurological examination and does not carry the 16p13.3 rearrangement.

Note S2: Identification of individuals present across multiple datasets.

Individual 6 was identified in both the 100kGP and NHS GMS datasets, whilst Individual 4 was in DECIPHER and in 100kGP. Although the detection of the same ultra-rare variant in a different genomic database associated with the same phenotype can help validate disease associations, care has to be taken to avoid double-counting individuals who may have been recruited at different ages and thus with slightly different sets of clinical terms. A recent example of this was two sisters with biallelic inactivation of *FILIP1* who were tested in the 100kGP[1] and also in an independent exome sequencing study.[2] As the clinical information was collected at different time points, it was not immediately apparent that these could be the same individuals. In the present scenario, the unusual nature of the duplication-triplication made the similarity highly suspicious, as an identical

duplication-triplication was considered unlikely to have occurred independently. Both sample overlaps were confirmed by the recruiting clinicians.

Note S3: Estimation of mosaicism using read count information.

For Individual 5, the SV appeared to be present in the unaffected father in a mosaic state due to the presence of far fewer split reads visible in IGV (**Figure S4**). The stepped increase in read coverage was also not as distinct. To help support the presence of genetic mosaicism and estimate the fraction of cells affected, read-count information was generated using samtools (v1.16.1) to estimate the relative copy number ratios and thus the precise level of mosaicism. We used the 166 kb distally duplicated and the central 64 kb triplicated segments and normalized read counts to a non-overlapping 10 Mb region on 16p12-13 (GRCh38, 16:10,000,000-20,000,000). Compared to 303 other genomes sequenced as part of the same delivery date batch, read counts in the duplicated/triplicated regions in the proband were increased by a factor of 1.56/1.93. In the father, the increase was lower at 1.21/1.32. These data supported the evidence from split-reads indicating the unaffected father to be mosaic and the SV was estimated to be present in 34-37% of nucleated blood cells.

Note S4: Systematic analysis of CN3 and CN4 gains in the 100k Genomes Project.

To follow up the initial genetic findings (i.e. overlapping *de novo* gains) from Individuals 1-3, we analyzed Genome Sequencing data from the 100kGP and performed a systematic analysis of SVs that intersected *ATP6V0C*. This was done using SVRare and 554,060,126 aggregated SVs that had been called by Manta and Canvas, across 71,408 participants. These individuals were from the rare-disease arm of the project. SV aggregation was using an 80% overlap threshold and rare variants were defined as those where the aggregated SV was present in 1% of individuals or less, as described previously.[3]

Overall, we identified 39 SVs, of which 13 were gains, 15 were deletions and the remaining 11 were inversions. The 13 gains were observed in a total of 23 100kGP participants. Of these 23 individuals, 18 harbored gains where the copy number had been estimated to be 3 (CN3). Only one of these individuals was reported to have ataxia and had been recruited to the 100kGP with a clinical diagnosis of Charcot-Marie-Tooth disease. Although the phenotype appeared less severe, this individual had several features reminiscent of the condition described here (ataxia, sensorimotor neuropathy, *pes cavus*). The individual had been recruited to the 100kGP in their late 60s as singleton and so the inheritance of the gain was uncertain. Viewing read alignments with IGV (v2.18.2) confirmed the gain to be a 356 kb tandem duplication (chr16:2,316,840-2,672,796; GRCh38, **Figure S24**) with no sign of any internal 4 copy (CN4) segment.

For the remaining 5/23 individuals, the Canvas algorithm had assigned an estimated copy number of 4. Review of read alignment data in IGV suggested that one of these CN4 calls, 87kb in size (chr16:2,476,315-2,563,519; GRCh38), was a false positive as it was in a sample with extremely wavey coverage (**Figure S24**) and a genome-wide total of 4,875 Canvas calls. Of the 337 rare disease genome datasets in the same sequencing batch, this was the highest number of Canvas calls and represents a 4.6x increase compared to the mean number of Canvas calls across all genome datasets in that batch. The remaining 4 individuals with CN4 calls all had a strikingly similar combination of features which included progressive ataxia, cerebellar hypoplasia and cognitive decline. Manual review of read alignments (**Figure 2B, Figures S3-5**) identified stepped increases in read coverage and split read-pairs that supported these four Canvas calls. The four 100kGP participants harboring these SVs correspond to Individuals 4-7 reported here (**Note S1** and **Table 1**).

Given the uneven distribution of ataxia-related phenotypes associated with CN3 genotypes (1/18, relatively mild) versus the CN4 genotypes (4/4, all severe), this enrichment is consistent with data from the UK Biobank where no individuals harbored a CN4 event and only 1/20 of the CN3 carriers have ataxia. Taken together, this suggests that the severe form of the condition is specific to the CN4 rearrangements. Given the lack of parental samples to test inheritance, further studies would be required to confirm whether the CN3 change predisposes to a milder form of the condition and whether copy number states higher than CN4 can result in an earlier onset condition.

Note S5: Oxford Nanopore sequencing methods for Individual 3.

Library preparation was performed according to the SQK-LSK110 Oxford Nanopore Technologies protocol. Briefly, one microgram of DNA underwent repair and dA tailing (Cat No. E7180, New England Biolabs) followed by AmpureXP bead cleanup (Cat No. A63881, Beckmann Coultier). A ligation reaction was then performed to attach the adaptor, AmpureXP bead cleanup performed followed by QC measurements on a genomic tape (Cat No. 5067-5365, Agilent) and Qubit fluorometry (Cat No. Q33230, Fisher Scientific). 50 fmols of library was then loaded onto a MinION R9.4.1 flowcell and ran for 72h, with basecalling using the high-accuracy model via Guppy 5.0.16. Mapping to hg38 was with minimap2 version 2.17.

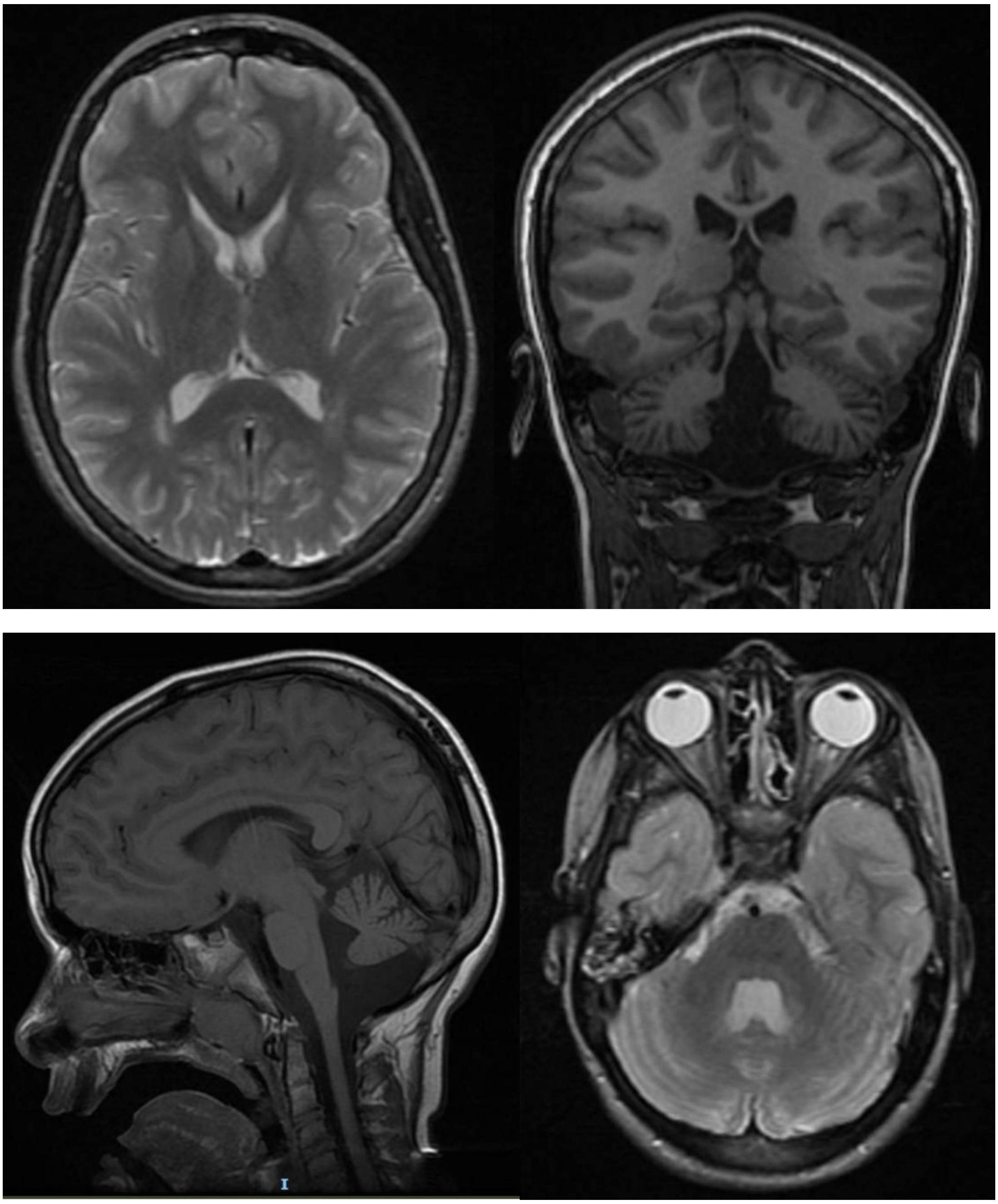

Figure S1: MRI scans from Family 1, Individual III-3.
Top row: Axial T2 weighted and Coronal 3D FSPGR (fast spoiled gradient echo) images demonstrate caudate atrophy and high T2 signal in the basal ganglia. Images acquired aged 12 years. Bottom row: Sagittal T1 and Axial T2 images show cerebellar hemisphere and vermian atrophy. Images acquired aged 9 years.

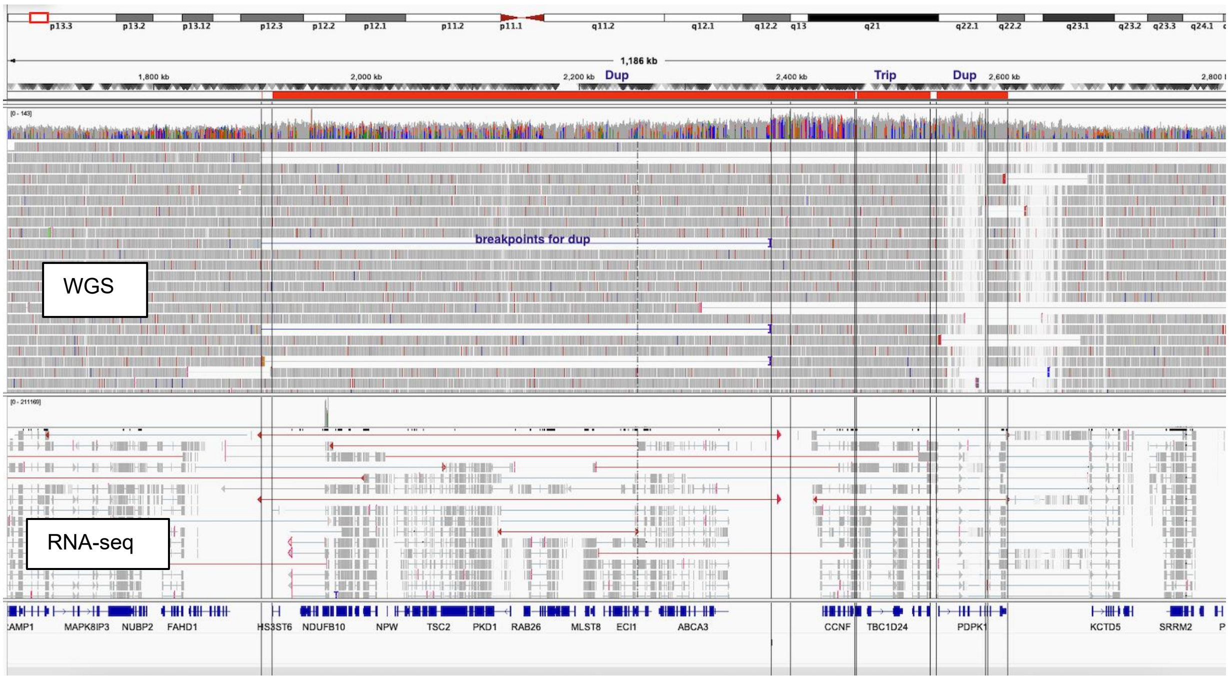

Figure S2: Read alignments from genome sequencing and RNA-seq data supporting a complex SV in Individual 2.

The distal end of the SV is shown by split read-pairs both mapping to the negative strand which are highlighted in blue. These breakpoints coincide with a stepped increase of read coverage from 2 to 3, then from 3 to 4 copies. Two similar read-pairs in the RNAseq data for this individual (lower track) also support the distal breakpoints. Red bars above the genome sequencing (GS) coverage track in IGV indicate the regions identified by microarray as dup/trip/dup. The GS data coverage therefore suggests that the distal duplicated segment is shorter and the triplicated segment larger than was documented by the microarray (**Figure S10**). Proximal breakpoints are likely to lie in the repeat regions where reads with low quality mapping are shown in white.

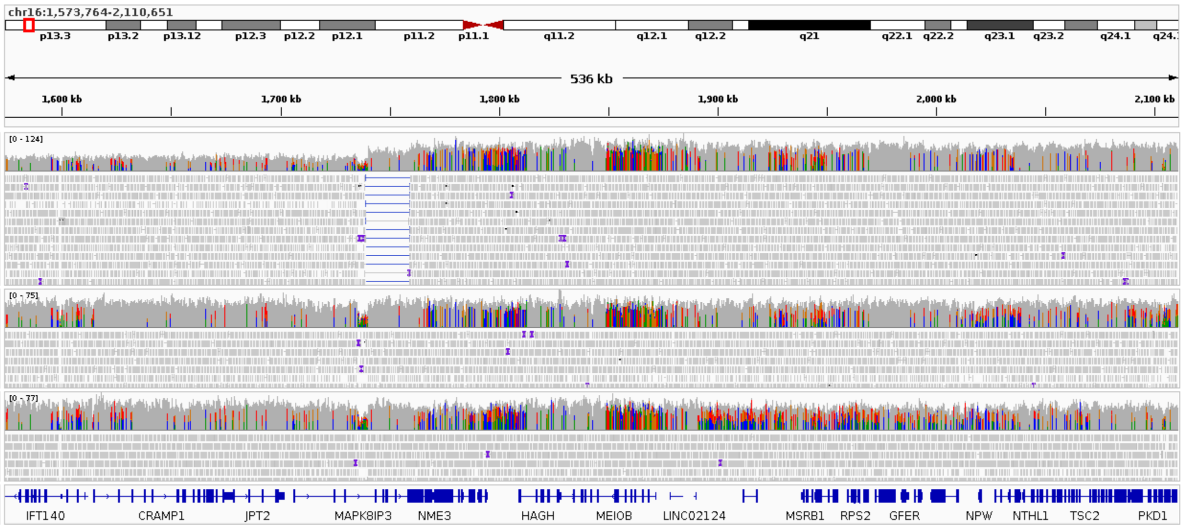

Figure S3: Read alignments from genome sequencing data supporting SV in Individual 4.

The distal end of the SV is shown for the proband (upper), where split read-pairs both mapping to the negative strand are highlighted in blue. These breakpoints coincide with a stepped increase of read coverage from 2 to 3, then from 3 to 4 copies. 150 bp reads are viewed in IGV using “collapsed” and

"view as pairs" settings. Parental data, shown in the bottom two tracks, indicate this SV to have arisen *de novo*.

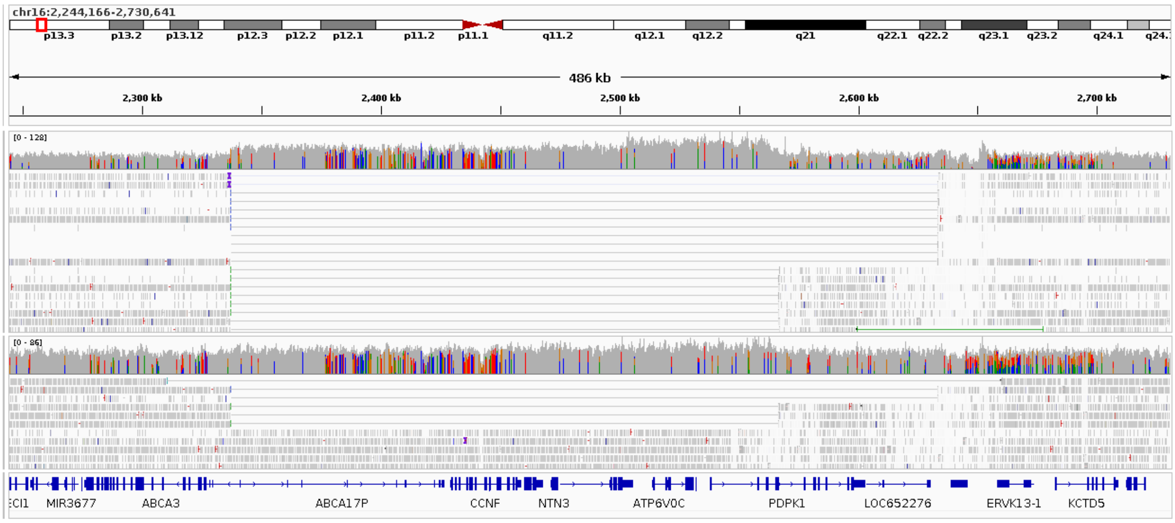

Figure S4: Read alignments from genome sequencing data supporting SV in Individual 5.

The full SV is shown for the proband (upper), where split read-pairs mapping from the start of the duplication to the proximal ends of the SV. The distal breakpoints coincide with a stepped increase of read coverage from 2 to 3, then decreased from 4 to 3 copies and from 3 to 2 copies. In contrast to the other SVs with genome sequencing data, the SV does not show clear negative to negative strand split read-pairs defining the distal end and so likely is not consistent with the Carvalho type structure. Nevertheless as the structure could not be resolved, the possibility of an inversion remains. 150 bp reads are viewed in IGV using "collapsed" and "view as pairs" settings. Paternal data (bottom track), shows a lower degree of increase in coverage and the similar split read-pairs are in a fewer percentage of reads, suggesting this individual to be mosaic for the variant.

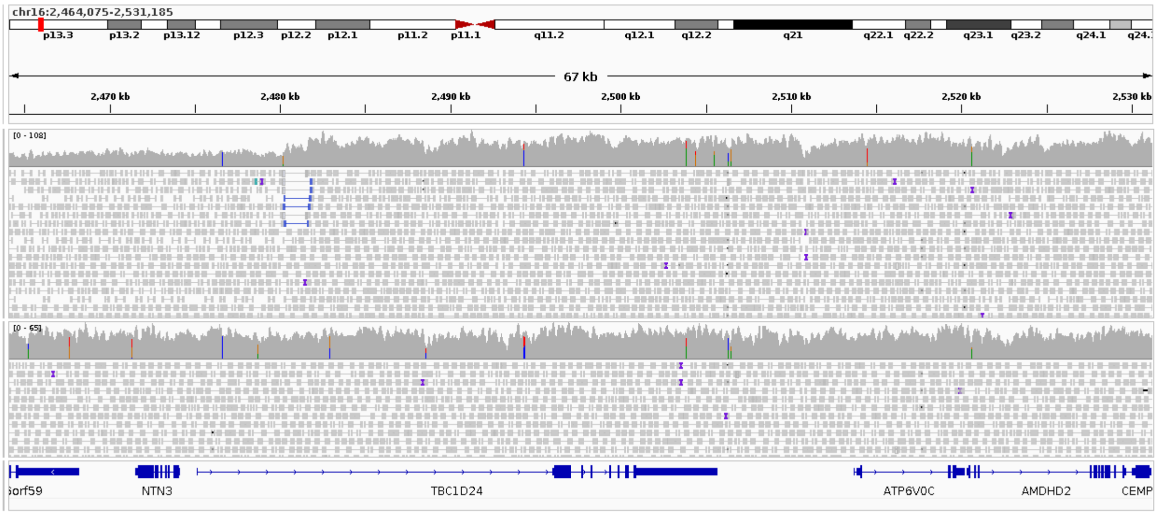

Figure S5: Read alignments supporting SV in Individual 6.

The distal end of the SV is shown for the proband (upper), where split read-pairs both mapping to the negative strand are highlighted in blue. These breakpoints coincide with a stepped increase of read coverage from 2 to 3, then from 3 to 4 copies and lie in the large first intron of *TBC1D24*. 150 bp

reads are viewed in IGV using “collapsed” and “view as pairs” settings. Genome sequencing data for the unaffected sister is shown in the bottom track indicate this individual has not inherited the variant. Although this individual was also sequenced as part of the NHS Genomic Medicine Service (GMS), which confirmed the SV, the data shown above is from the 100k Genomes Project.

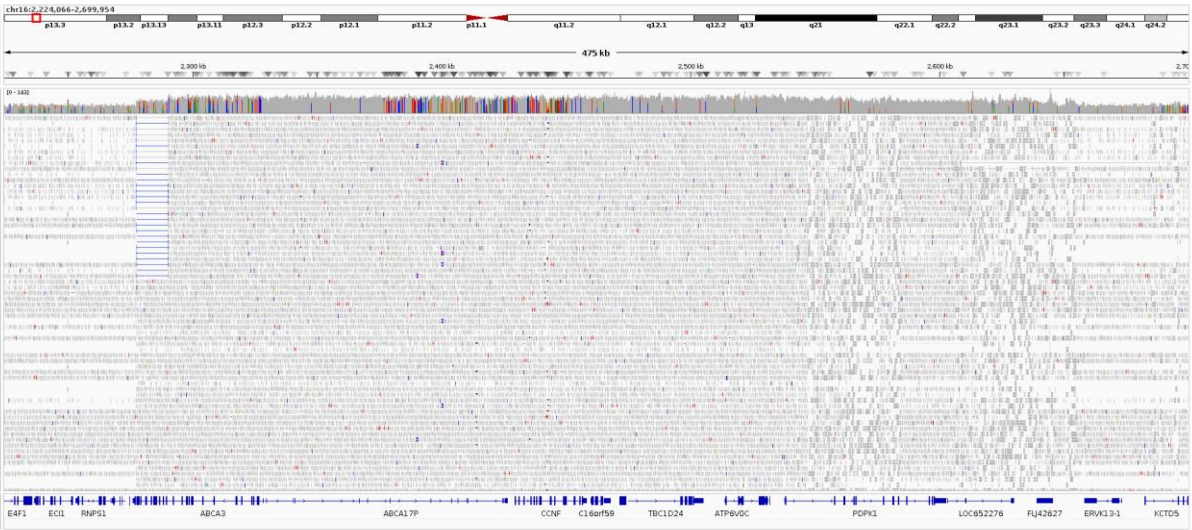

Figure S6: Read alignments from genome sequencing data supporting the SV in Individual 8.

In contrast to the other UK families which were sequenced as part of the 100kGP, this individual was sequenced as part of the NHS GMS. The distal ends of the SV are where split read-pairs both mapping to the negative strand are highlighted in blue. These breakpoints coincide with a stepped increase of read coverage from 2 to 3, then from 3 to 4 copies and lie in *ABCA3*. 150 bp reads are viewed in IGV using “collapsed” and “view as pairs” settings.

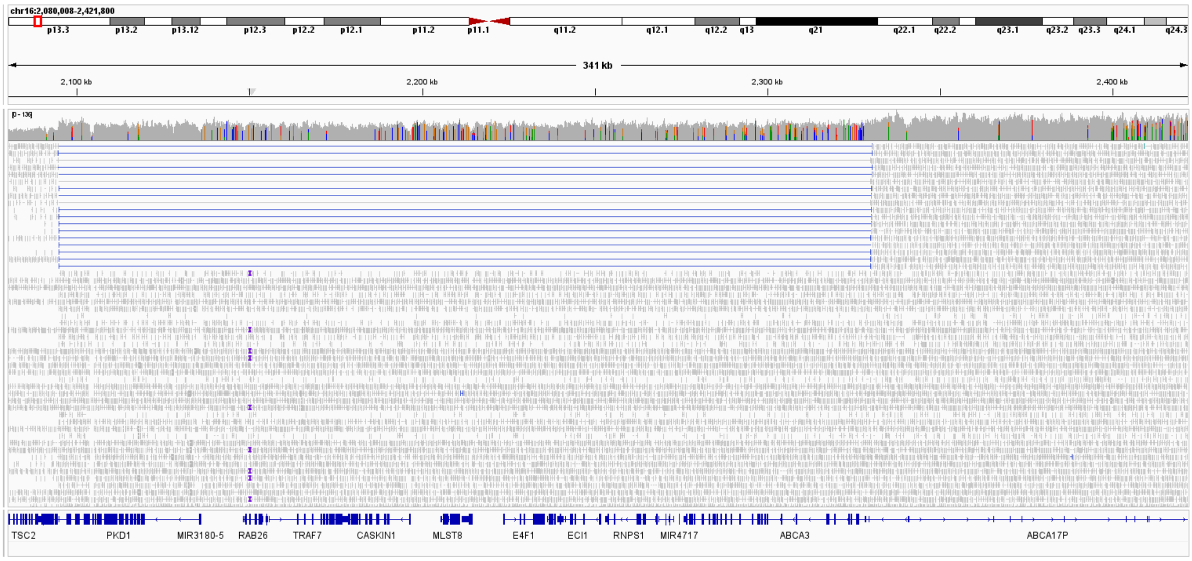

Figure S7: Read alignments from genome sequencing data supporting the SV in the proband (III-3) from Family 1.

The distal end of the SV is shown in IGV, where split read-pairs both mapping to the negative strand are highlighted in blue. These breakpoints coincide with a stepped increase of read coverage from 2 to 3, then from 3 to 4 copies. 150 bp reads are viewed using “collapsed” and “view as pairs” settings. This distal breakpoint was further supported by the presence of a fusion transcript detected between *PKD1* and *ABCA3* (**Figure S16-17**).

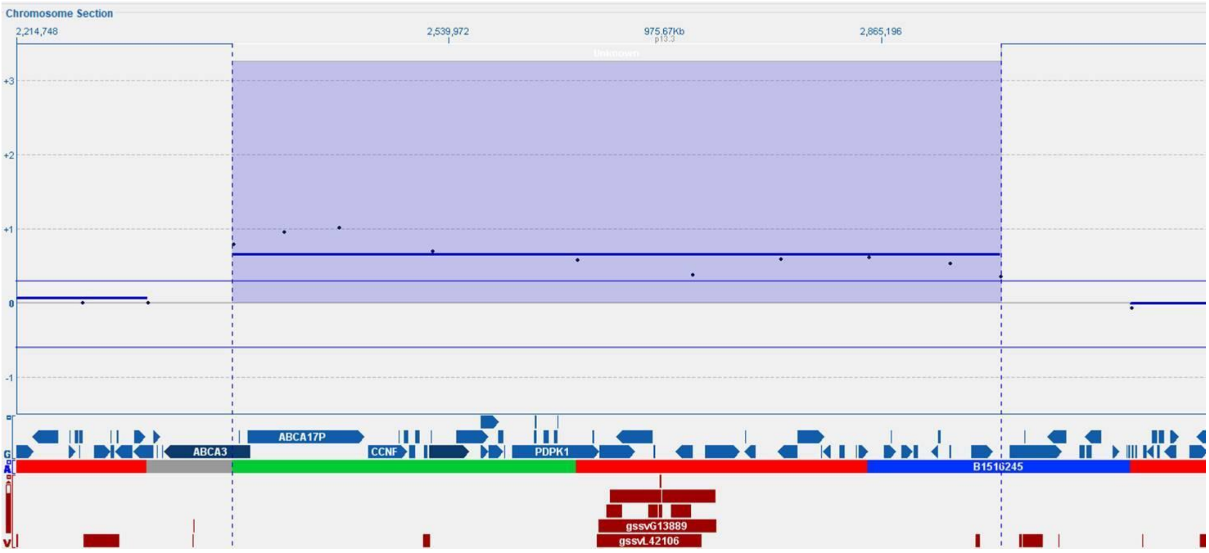

Figure S8: Low resolution array-based comparative genomic hybridization (aCGH) data for Individual 3.

Probe intensity data is plotted against GRCh37/hg19 chromosome position where results are consistent with a copy number increase for this locus on 16p13.3. There are 10 probes inside the SV and at this resolution it is hard to distinguish whether there is a triplicated segment within the duplication. The algorithmic duplication call is shaded in blue and represents the minimal region. Experiments were performed using the 8x60K constitutional v3.0 array. Hybridizations were carried out using pooled, sex matched DNA as a control and data analysis was with CytoSure Interpret v4.9 (Oxford Gene Technology).

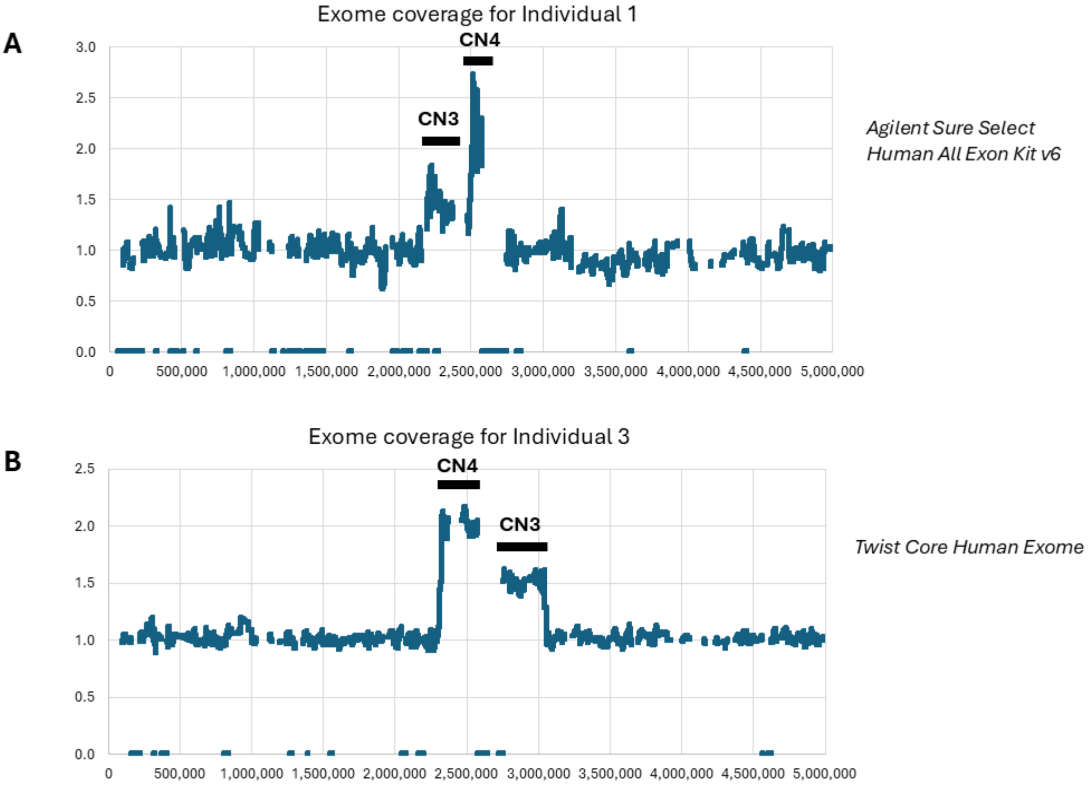

Figure S9: Read coverage analysis of exome data identifies triplicated segments.
Coverage in the proband is normalized to the mean coverage across both unaffected parents who were sequenced using the same methodology. Relative coverage was then plotted for overlapping windows corresponding to 3kb of the target region. Data shown is mapped to GRCh37/hg19. Data for Individuals 1 and 3 is shown in panels A and B, respectively. Although different capture kits were used, in both cases sequencing was performed on the Illumina NextSeq machine.

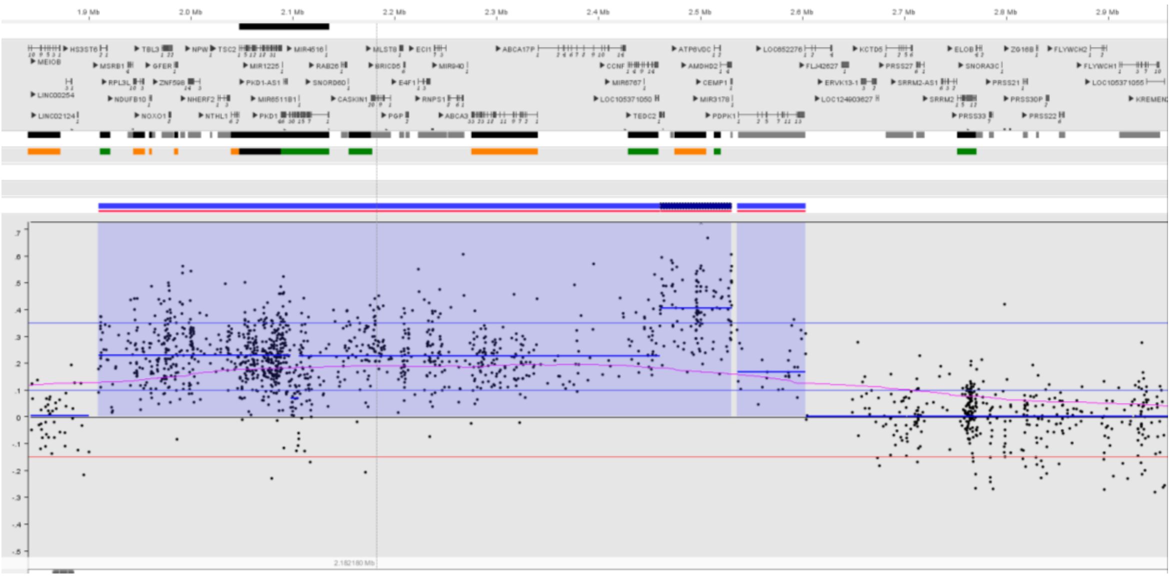

Figure S10: Infinium Global Diversity Array-Cyto data for Individual 2.

The ~1.8M probes on this array results in a much higher resolution than had been obtained with earlier array testing. Probe intensity data is plotted against chromosome position where results are consistent with a small triplication of 16p13.3 embedded within a duplication. Region shown is chr16:1,842,971-2,964,603 (GRCh38). Confident software calls are shown in blue and purple shading and OMIM morbid genes are highlighted above in green/orange. A pink line shows a moving average of the probe intensity data. The genes *ATP6V0C* and *AMDHD2* are localised within the triplicated segment.

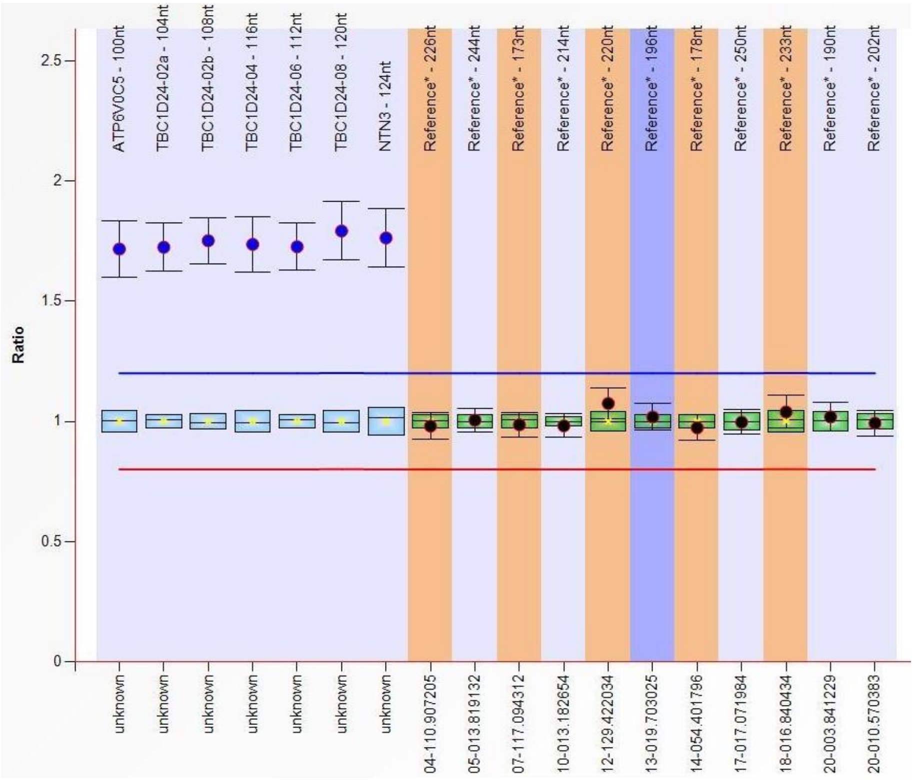

Figure S11: Data from MLPA testing for Individual 7 confirmed the variant to be *de novo*.

MLPA kit P200-B1 (MRC Holland), which contains reference probes and control fragments was customized to include 7 additional probes corresponding to *ATP6V0C*, *TBC1D24* and *NTN3*. Although initially reported as a heterozygous duplication, retrospective analysis shows all probes within the structural variant (SV) have an increased signal ratio of between 1.5 and 2 which equates to an estimated copy number of between 3 and 4. A retrospective comparison of MLPA probe sequences with genome sequencing defined SV coordinates indicated that all 7 probes lay within the triplicated region (https://genome.ucsc.edu/s/AlistairP/16p13.3_F7_MLPA). Previous testing by array-CGH using an Agilent ISCA 60K oligoarray had also failed to distinguish between duplication and triplicated segments.

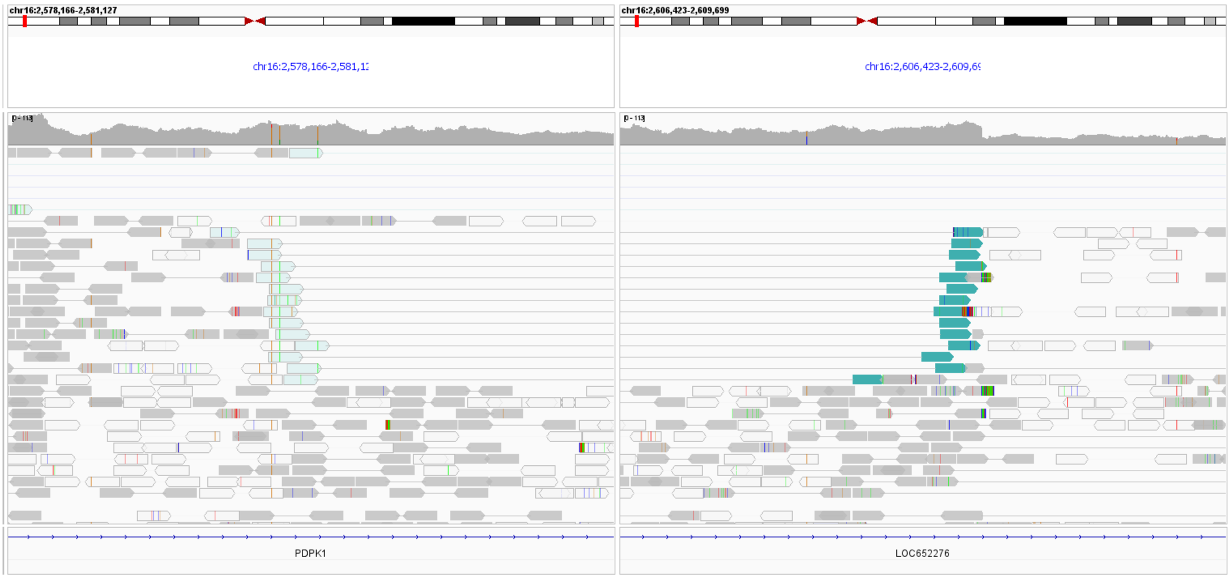

Figure S12: Read alignments from genome sequencing data supporting SV in the proband (III-3) from Family 1.

The proximal ends of the SV are shown in IGV using split view, where split read-pairs both mapping to the positive strand are highlighted in teal. The most proximal breakpoint coincides with a clear decrease in read coverage, corresponding to a drop from 3 to 2 copies. 150 bp reads are viewed using “view as pairs” settings. Due to the presence of the large palindromic repeat, many reads have a low mapping quality and these are shaded in white.

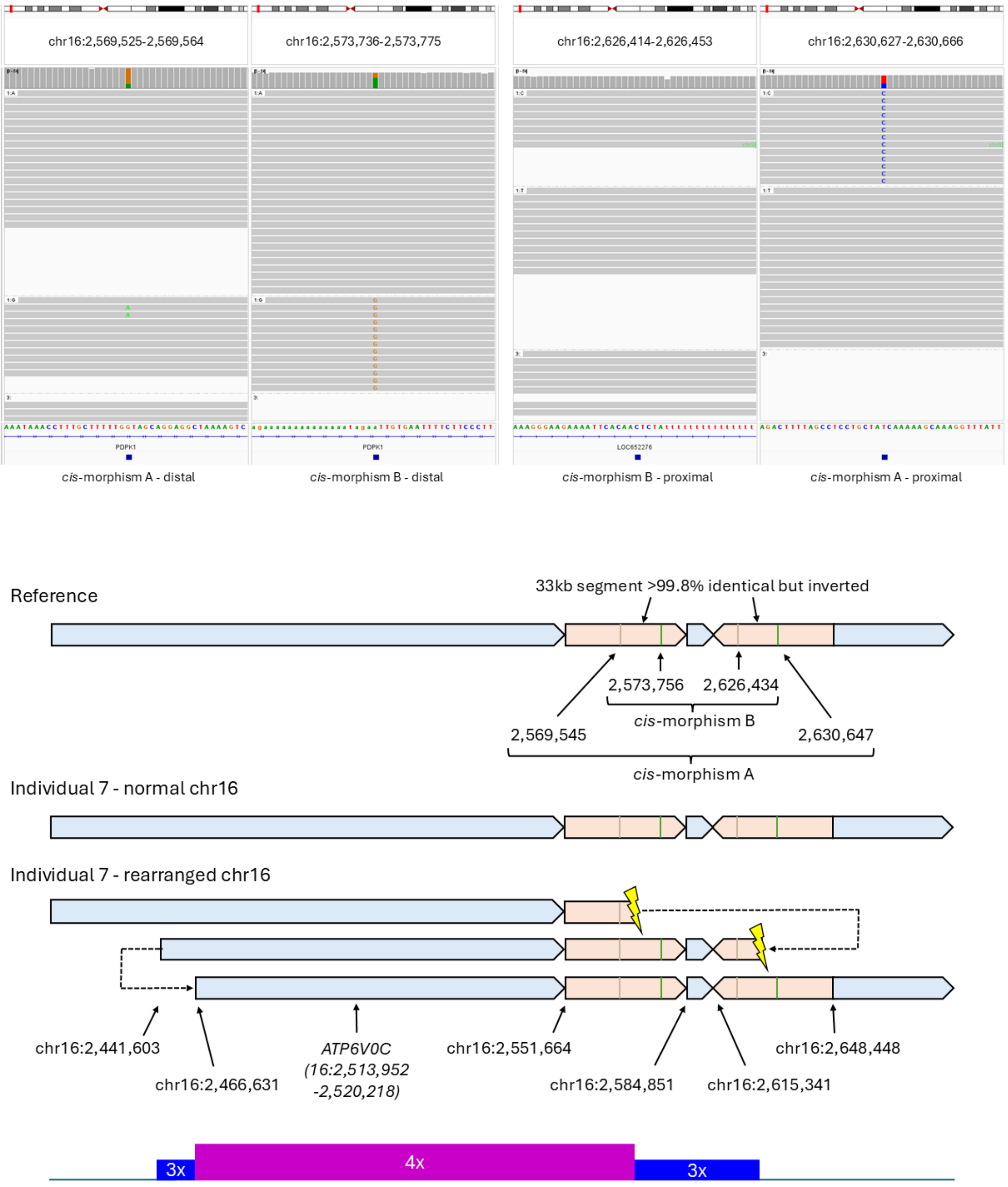

Figure S13: Split-screen IGV screenshot showing HiFi PacBio read alignments at *cis*-morphic sites in Individual 7.

The GRCh38 genomic coordinates of these 4 *cis*-morphic sites are: 16:2569545, 16:2573756, 16:2626434 and 16:2630647 and all are within the palindromic repeat, close to the suspected proximal end of the SV. Reads are grouped by base at position 16:2573756 and 16:2630647. Several reads span between *cis*-morphisms A and B. Several reads harbor one but not the other *cis*-morphism and this suggests that the proximal SV breakpoints may lie in the 4.2 kb interval between these positions. Positions of *cis*-morphic sites are available in an interactive UCSC session http://genome.ucsc.edu/s/AlistairP/16q13.3_cismorphismsV2. The schematic diagram includes a

subway plot that explains how the proximal breakpoint of the rearrangement could explain both the anomalous PacBio reads and the changes in copy number state.

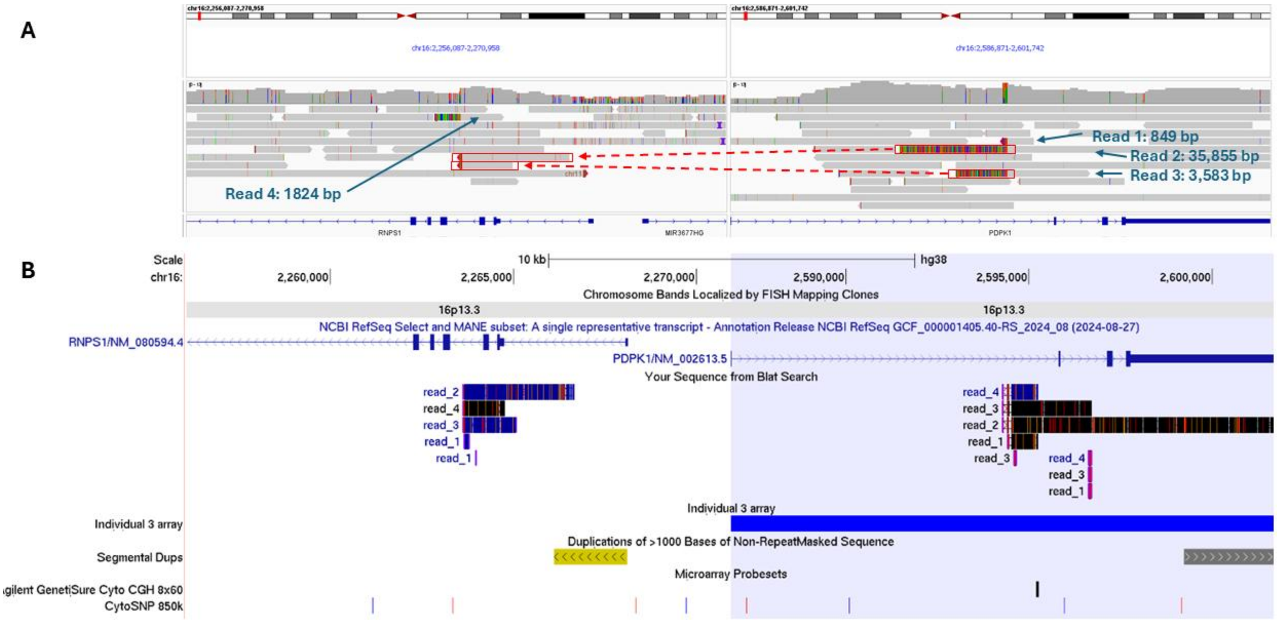

Figure S14: Low coverage Oxford Nanopore sequencing resolves breakpoint in Individual 3.

A) Read alignments shown in IGV. Regions shown are chr16:2,256,087-2,270,958 and chr16:2,586,871-2,601,742 (GRCh38). B) UCSC multi-region browser graphic showing BLAT search results for the 4 reads (as labelled in panel A) which span the distal breakpoint. This session is available interactively at https://genome.ucsc.edu/s/AlistairP/16p3.3_I3_nanopore. The duplication start point was underestimated by around 50 kb from the array testing. The structure for this SV is most similar to that seen in Individual 5 (**Figure S4**). The black and dark blue shading for the BLAT sequence track denotes whether the sequence maps to the positive or negative strand and so the results indicate some form of inversion.

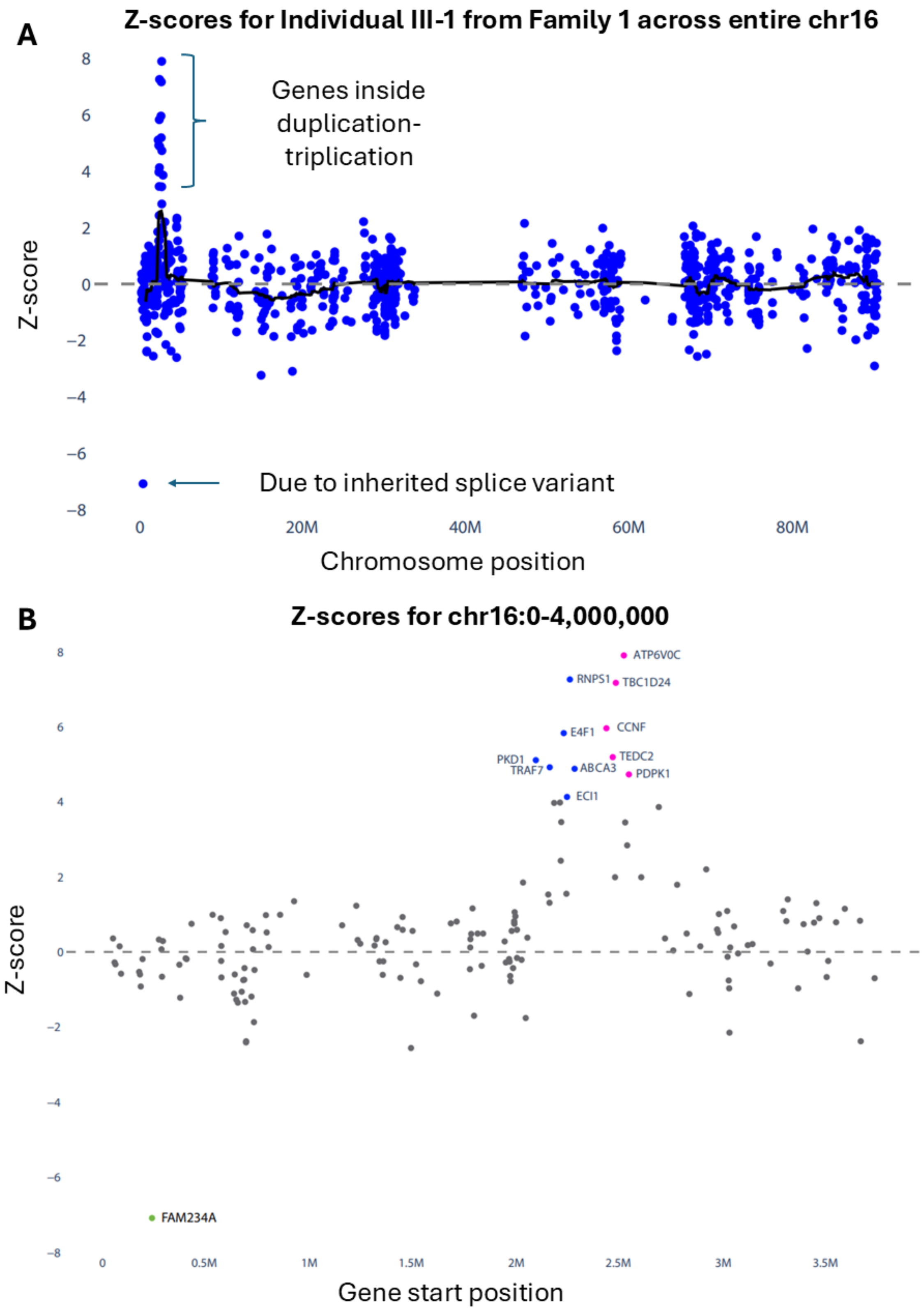

Figure S15: RNA-seq expression Z-scores for Individual III-3 from Family 1 plotted against chromosome position.

A) Whole chromosome view shows clear outlier at the end of the short arm (16p13.3) which coincides with the position of the duplication-triplication. Black line represents a moving-window average where the window size is dynamically set to 5% of the length of the chromosome. B) As

above, but zoomed in to chr16:0-4,000,000 and with gene symbols labelled. Although several genes within the 16p13.3 SV show higher than expected expression, the most significant result was for *ATP6V0C*, with a Z-score of 7.9. Reduced expression of *FAM234A* (Z-score of -7.1) was likely due to a heterozygous NM_032039.4:c.-140+2T>G variant. Significantly upregulated genes that lie within the 16p13.3 duplication or triplication segments are highlighted in blue and purple, respectively. Chromosome 16 positions are based on the GRCh38 reference.

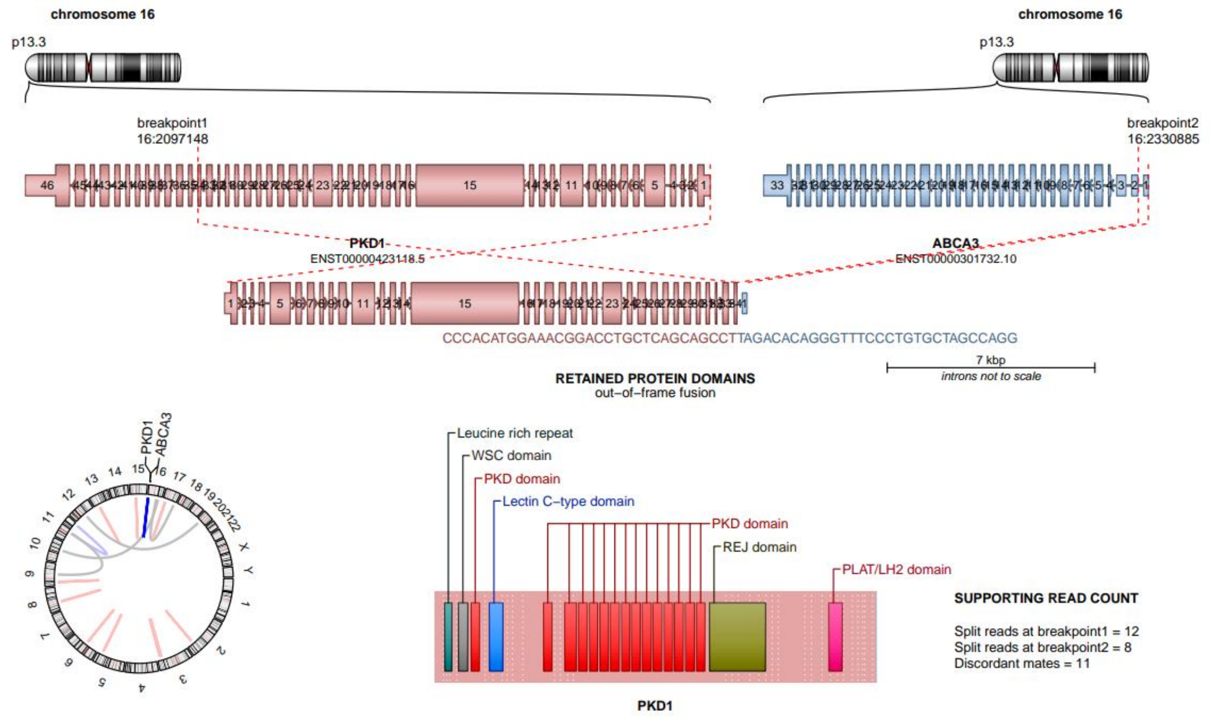

Figure S16: RNA-seq data identified **a** *PKD1-ABCA3* fusion transcript in Individual III-3 from Family 1.

The *PKD1-ABCA3* fusion transcript is highlighted in the circus plot in blue and the types and numbers of supporting reads are summarized. The fusion was ranked as the top hit by Arriba (https://github.com/oicr-gsi/arriba). The fusion junction is between the end of *PKD1* exon 34 (ENST00000423118.5) in the coding direction and the first intron of *ABCA3* (ENST00000301732.10) in the non-coding direction. The sequences corresponding to the breakpoint are shown in https://genome.ucsc.edu/s/AlistairP/16p13.3_F1_fusion in the BLAT search track. Exons 1-34 of *PKD1* are in an inverted orientation.

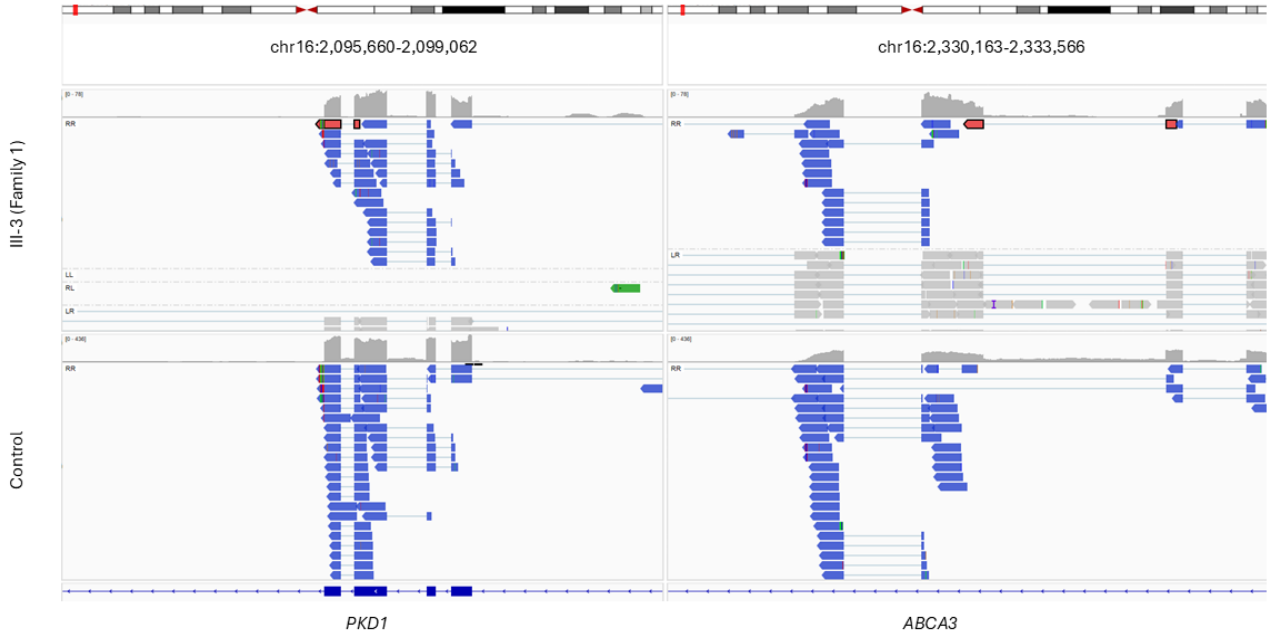

Figure S17: RNA-seq data for Individual III-3 of Family 1 supports presence of a fusion transcript.

Split-screen IGV screenshot showing RNA-seq read-alignments supporting the presence of a *PKD1-ABCA3* fusion transcript. The two reads highlighted in red are pairs that span the junction of the gene-fusion and correspond closely to the distal SV breakpoint. Both map to the negative strand of GRCh38. Data is for Individual III-3 from Family 1 (above) and a control RNA-seq sample (below).

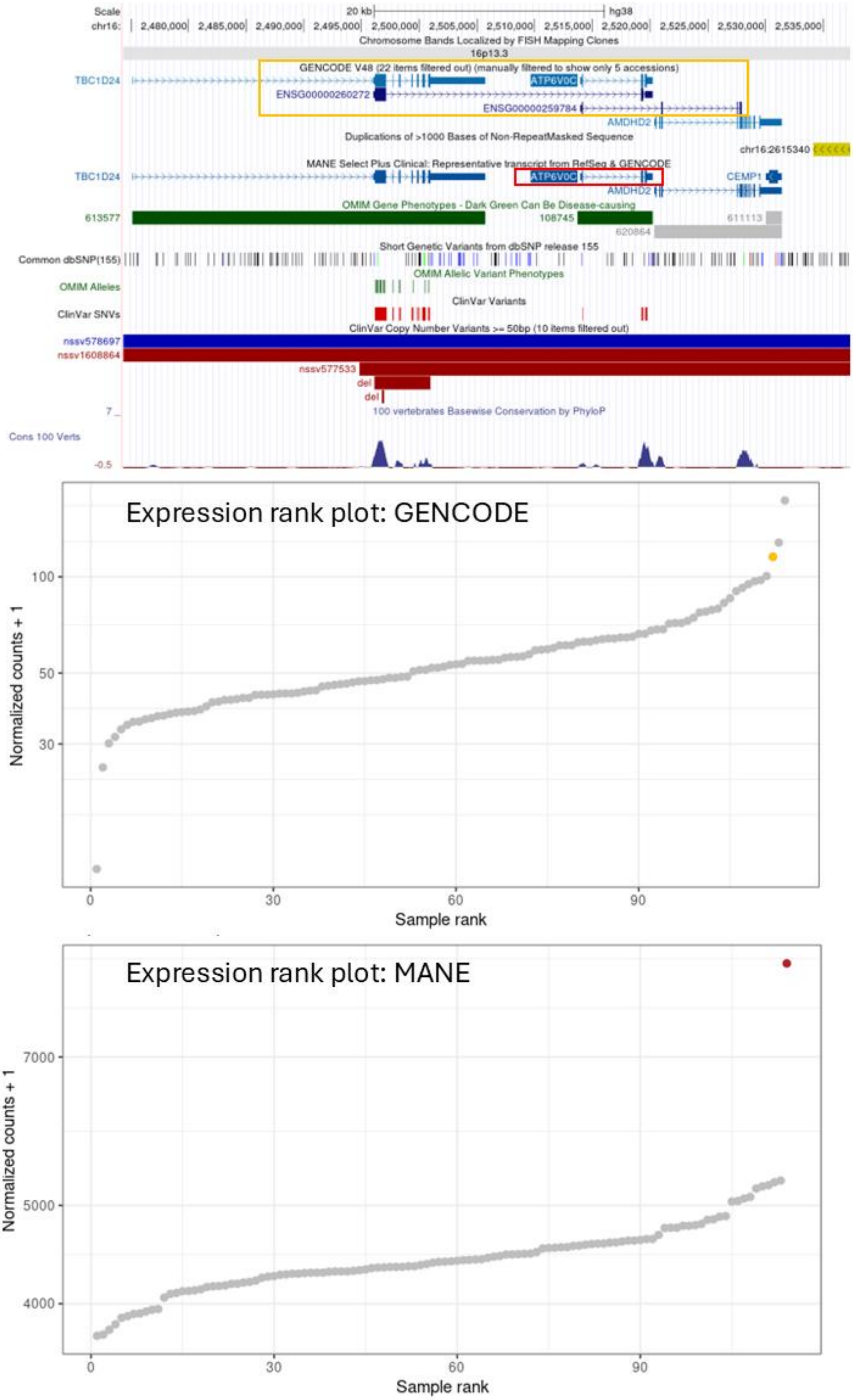

Scale
chr16:
20 kb
hg38
2,480,000
2,485,000
2,490,000
2,495,000
2,500,000
2,505,000
2,510,000
2,515,000
2,520,000
2,525,000
2,530,000
2,535,000
Chromosome Bands Localized by FISH Mapping Clones
16p13.3
GENCODE V48 (22 items filtered out) (manually filtered to show only 5 accessions)
TBC1D24
ATP6V0C
ENSG00000260272
ENSG00000259784
AMDHD2
Duplications of >1000 Bases of Non-RepeatMasked Sequence
chr16:2615340
MANE Select Plus Clinical: Representative transcript from RefSeq & GENCODE
TBC1D24
ATP6V0C
CEMP1
AMDHD2
OMIM Gene Phenotypes - Dark Green Can Be Disease-causing
613577
108745
611113
620864
Short Genetic Variants from dbSNP release 155
Common dbSNP(155)
OMIM Allelic Variant Phenotypes
OMIM Alleles
ClinVar Variants
ClinVar SNVs
ClinVar Copy Number Variants >= 50bp (10 items filtered out)
nssv578697
nssv1608864
nssv577533
del
del
7
100 vertebrates Basewise Conservation by PhyloP
Cons 100 Verts
-0.5
Expression rank plot: GENCODE
Normalized counts + 1
100
50
30
0
30
60
90
Sample rank
Expression rank plot: MANE
Normalized counts + 1
7000
5000
4000
0
30
60
90
Sample rank

Figure S18: RNA-seq data for Individual 2 highlights the importance of using appropriate transcript annotations.

UCSC genome browser session showing Genecode V48 (orange box) and MANE Select gene annotations (red box) for *ATP6V0C* (ENSG00000185883.12), which are identical. However, for the Genecode annotations, there are two additional fusion transcripts, for *TBCD1D24-ATP6V0C* (ENSG00000260272.1) and *ATP6V0C-AMDHD2* (ENSG00000259784.1). An interactive view is available at https://genome.ucsc.edu/s/AlistairP/ATP6V0C_RNAseq_artifact. Lower panels show RNA-seq results from lymphoblast-derived RNA for Individual 2 which was part of a cohort (N=114) analyzed with OUTRIDER. With the Genecode annotations, normalized read counts for *ATP6V0C* are typically low (30-100) and Individual 2 was ranked 3rd in terms of relative expression (Z-score = 2.34). Close scrutiny suggested that these results were being confounded by Gencode annotations for fusion genes that overlap the MANE *ATP6V0C* transcript. After repeating the OUTRIDER analysis using just the "MANE select" annotations, normalized *ATP6V0C* read counts across the cohort were much higher (4000-5000) and Individual 2 was a clear outlier, ranked as having the highest relative expression for this gene (FC=1.95, Z-score = 6.86).

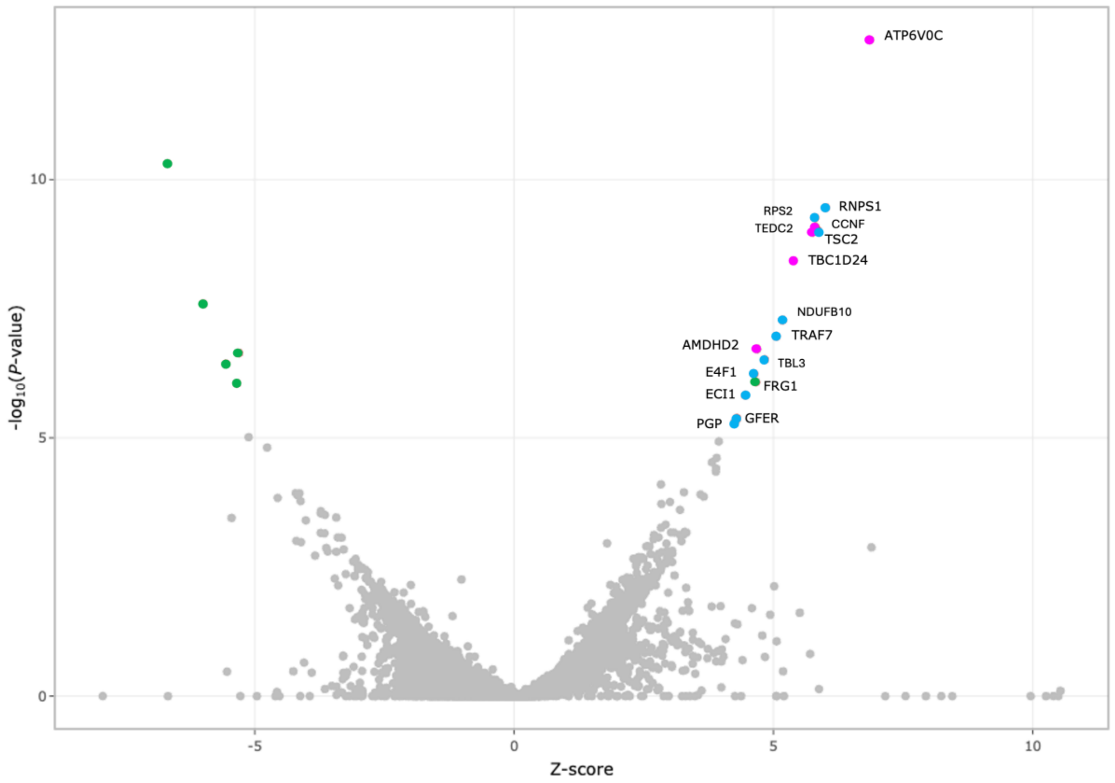

Figure S19: Volcano plot of RNA-seq data for Individual 2.

RNA-seq data for lymphoblast-derived material from an individual with rare rearrangement on 16p13.3 confirms aberrant gene expression. Genome-wide volcano plot showing genes that had significantly aberrant expression (*P*-value < $1x10^{-5}$) in Individual 2. Significantly upregulated genes that lie inside the 16p13.3 duplication/triplication are highlighted in blue/purple, respectively. Genes outside the SV are labelled in green. Similar to the results for the proband in Family 1 (**Figure 3A)**, the most significant result was *ATP6V0C,* which lies inside the triplicated segment and has a Z-

score of 6.86. Of the 16 most significantly upregulated genes, 15 lay within the 16p13.3 rearrangement (**Table S3**).

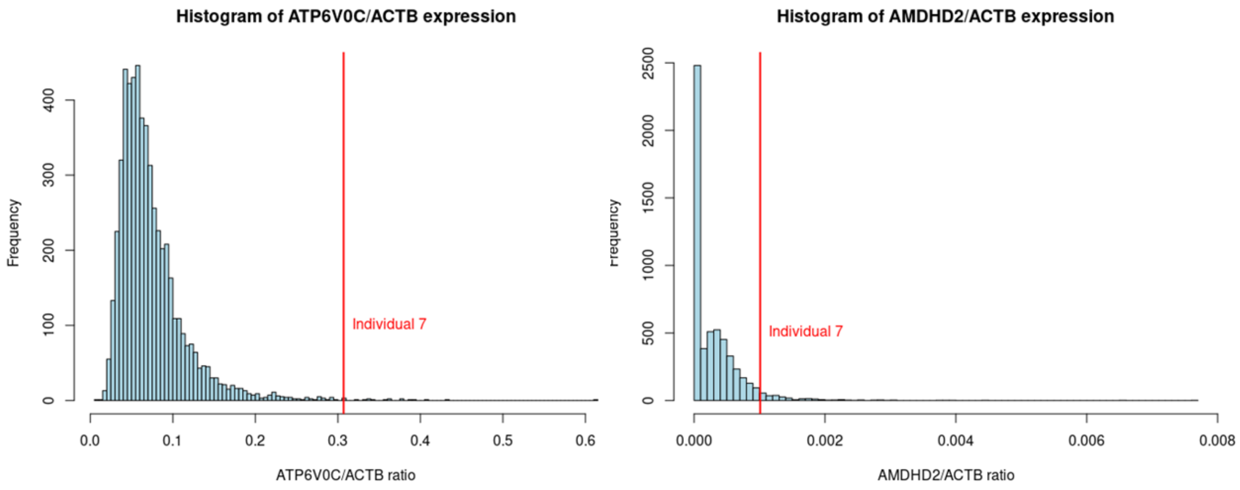

Figure S20: Relative RNA expression of *ATP6V0C* and *AMDHD2* is elevated in Individual 7.

When normalized to *ACTB*, a second housekeeping gene, expression of *ATP6V0C* was 4.16x above the mean and *AMDHD2* was 3.48x above the mean. Results for all 5,546 datasets are shown. Data normalized to *GAPDH* are shown in **Figure 3C-D**.

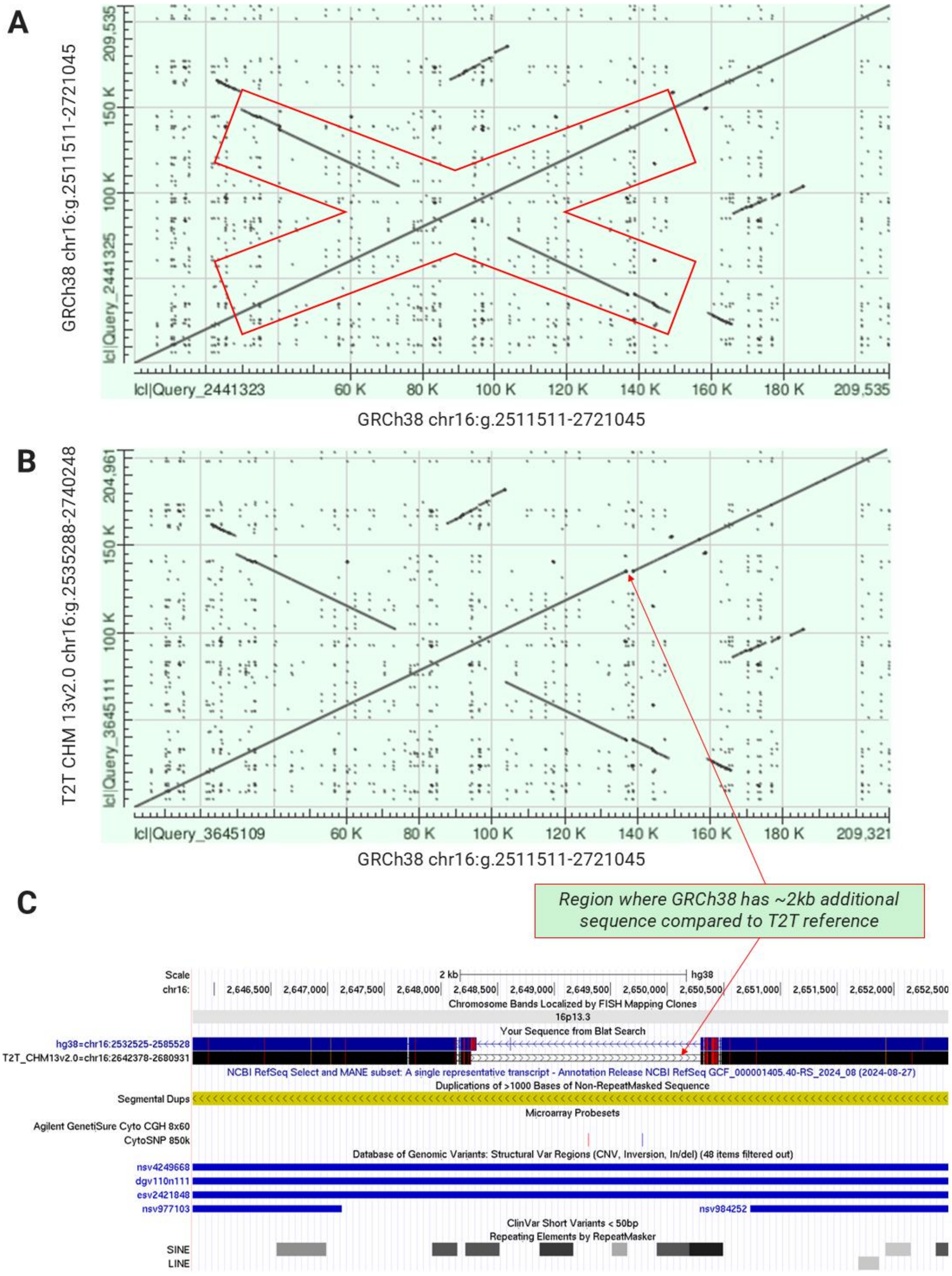

Figure S21: Comparison of the palindromic repeat sequence on 16p13.3 within and between different reference builds.

A) Dotplot creased using 209.5 kb of sequence from 16p13.3 showing the presence of a large palindrome-like repeat (red cross) present in GRCh38. B) A similar comparison of the same region between GRCh38 and the latest available reference from the Telomere-to-Telomere consortium

(CHM 13v2.0) highlights a site that differs between the two builds. Dotplots were created using the NCBI BLAST tool and default settings, with the “highly similar sequences (megablast)” option. C) Genome browser graphic for the discordant region showing that GRCh38 contains an additional ~2kb of sequence compared with both the CHD 13v2.0 genome and with the paralogous repeat nearby in GRCh38. An interactive UCSC session for this region (chr16:2,645,808-2,652,528) is available at https://genome.ucsc.edu/s/AlistairP/16p13.3_hg38vsT2T. The black and dark blue shading for the BLAT sequence track denotes whether the sequence maps to the positive or negative strand. In addition to the change highlighted above, we note that GRCh38 also contains an alternate contig (KQ090026v1) that corresponds to alternative haplotype for a nearby region.

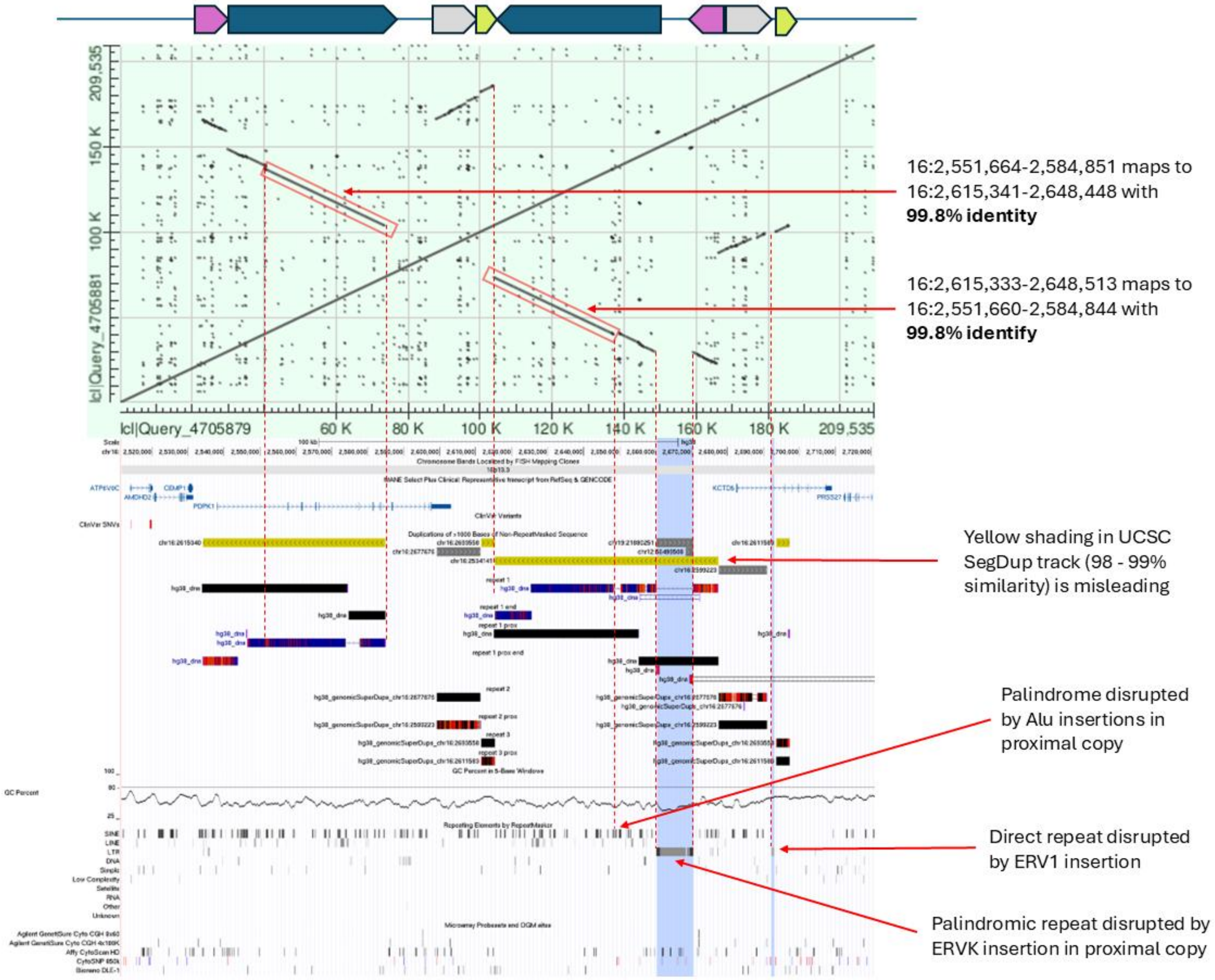

Figure S22: Further review of GRCh38 sequence for the palindromic repeat region (chr16:2,511,511-2,721,045).

The dotplot generated by the NCBI the Blat2seq tool highlights that within the annotated segmental duplication there is a segment of 33kb with a particularly high level of sequence identity at 99.8%. This repeat is present in a plus/minus orientation and thus represents a palindrome-like structure. Long terminal repeat (LTR) element insertions which disrupt the proximal repeat units are highlighted in light blue. Dotted red lines connect the ends of the repeat segments identified in the dotplot to the equivalent positions on the BLAT sequence tracks on the UCSC browser graphic to help uncover the genomic features that interrupt the repeat units. BLAT tracks can only show 40kb

of sequence so the main repeat is split into two BLAT tracks. The schematic diagram above the dotplot represents a simplified representation of the repeat structure. GC content across the region is unremarkable. An interactive version of UCSC graphic is available at https://genome.ucsc.edu/s/AlistairP/16p13_palindrome_v3.

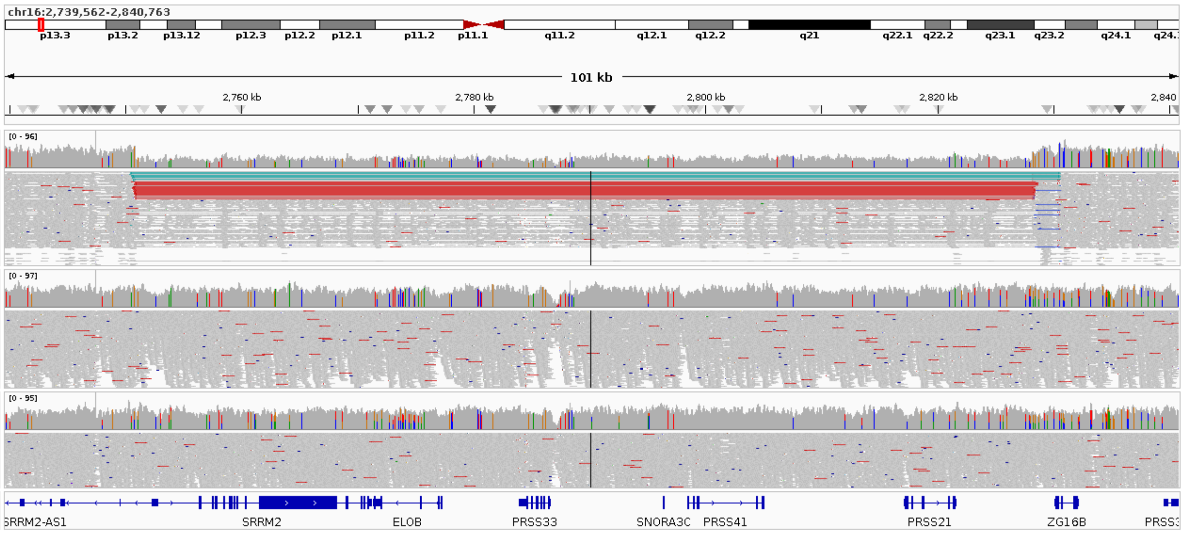

Figure S23: Read alignments supporting a complex deletion-inversion variant on 16p13.3 in an individual from the NHS Genomic Medicine Service.

The SV is shown in IGV, where split read-pairs both mapping to the positive strand are highlighted in teal and pairs mapping to the negative strand in blue. Read-pairs highlighted in red are those that span right across the 166 bp inserted/inverted segment. Although reported clinically as chr16:g.2750923_2828204del (GRCh38), this ignores the presence of the internal inversion. The 150 bp reads are viewed using the "squished" and "view as pairs" settings. Parental data, shown in the bottom two tracks, indicate this SV to have arisen *de novo*. As *SRRM2* lies in the middle of this heterozygous deletion, this finding is consistent with a diagnosis of autosomal dominant intellectual developmental disorder, type 72 (MIM: 620439). The individual's clinical features, focal-onset epilepsy, learning disability requiring specialist schooling, ADHD, and behavioral difficulties, are concordant with the phenotype previously described for this condition. In addition, primary hypothyroidism with constitutional short stature (0.4th centile) was noted, a feature not previously associated with *SRRM2*-related neurodevelopmental disorder, and potentially representing phenotypic expansion. This observation supports our prior report of complex SVs at this locus.[4]

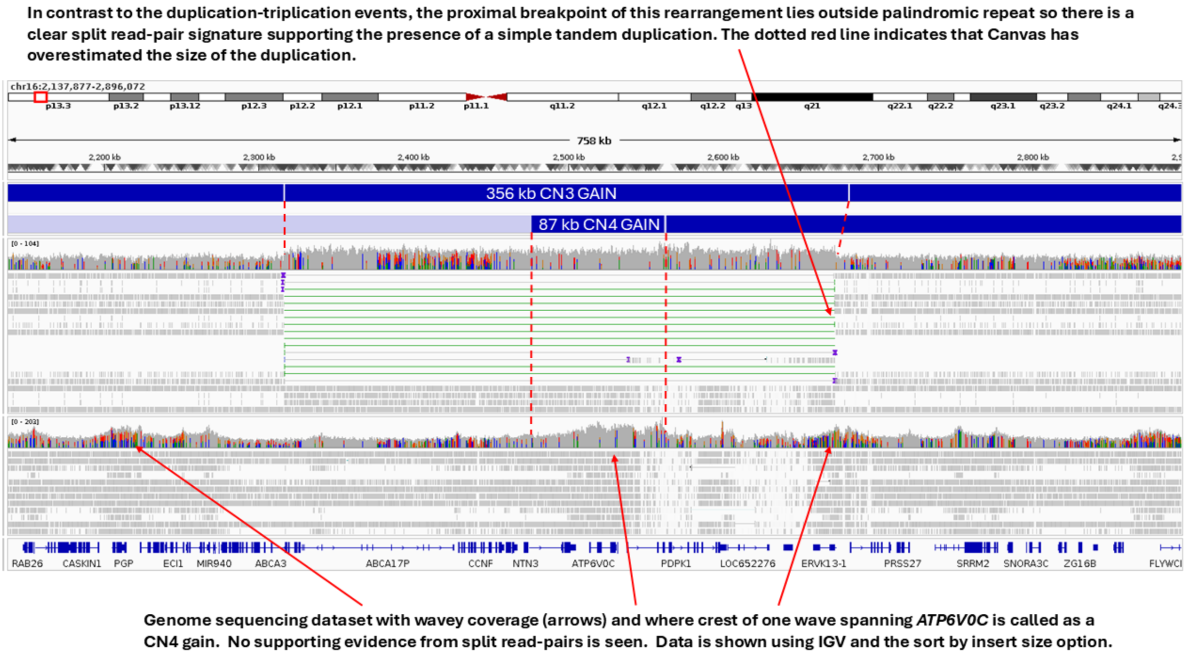

Figure S24: Read alignments showing a tandem-duplication and a false positive triplication call from the 100k Genomes Project.

IGV screenshot showing read alignments confirming the presence of a 356 kb tandem duplication (middle track), where 150 bp split read-pairs coincide with an increase in read depth. The proximal end of the duplication was overestimated by Canvas. In the bottom track, a wavey pattern is seen (see highlighted red arrows) that suggests poor sample quality. There are also no discrete steps in read coverage and no split read-pairs to support the 87 kb SV call.

	Individual 1	Individual 2	Individual 3	Individual 4	Individual 5	Individual 6	Individual 7	Individual 8	Family 1
**CNV detection by**	Array + ES	Array + GS	Array, ES, LR	Array + GS	GS	GS	GS + LR	GS	Array + GS
**Duplication coordinates** (chr16:GRCh38)<br><br>**Duplication size**	2,137,455-2,585,920†<br><br>448 kb	1,901,911-2,606,201‡<br><br>704 kb	2,263,630-2,903,967†§<br><br>640 kb	1,739,210-2,657,000<br><br>918 kb	2,337,167-2,566,956<br><br>230 kb	2,480,209-2,637,000<br><br>157 kb	2,441,603-2,628,540[1]<br><br>187 kb	2,277,344-2,655,000<br><br>378 kb	2,094,750-2,608,383<br><br>514 kb
**Triplication coordinates** (chr16:GRCh38)<br><br>**Triplication size**	2,499,243-2,529,370<br><br>30 kb	2,382,641-2,561,336<br><br>179 kb	2,277,490-2,594,500<br><br>317 kb	1,758,994-2,570,189<br><br>811 kb	2,503,226-2,566,956<br><br>64 kb	2,481,567-2,566,956<br><br>85 kb	2,466,631-2,571,650<br><br>105 kb	2,290,102-2,586,432<br><br>296 kb	2,330,064-2,579,700<br><br>250 kb
**Inheritance**	*de novo*	*de novo*	*de novo*	*de novo*	Father mosaic	NK	*de novo*	NK	Maternal/NK[2]

Table S1: Details of overlapping duplication/triplications involving 16p13.3 in the 8 individuals and one multiplex family in this study.

ES, Exome sequencing; GS, Genome Sequencing; LR, Long-read genome sequencing; NK, not known. Genomic coordinates are based on NC_000016.10 (GRCh38). There remains some uncertainty about the precise coordinates, particularly at the proximal end and for Individuals 1 and 3 where only low resolution (†) array and exome data was available. ‡, For Individual 2, although high resolution array data was available, coordinates are taken from genome sequencing data. §, Individual 3 where low resolution array and exome was initially performed but long-read sequencing was also undertaken. Although array/exome coordinates are reported, Oxford Nanopore data suggests a distal breakpoint at 16:2,263,633:2,594,508 (**Figure S14**). [1]For Individual 7, the proximal breakpoints are taken as the midpoint between the two *cis*-morphisms. [2]Presumed *de novo*; not paternally inherited, no DNA available from deceased mother.

Table S2: Information for genes on 16p13.3 within or near the shared region of triplication.

Missense constraint and pLI scores are from gnomAD 4.1.0. FC, fold-change. aa, amino-acids. AR, autosomal recessive. AD, autosomal dominant. †Also linked to Deafness, autosomal dominant 65 (MIM: 616044) and Deafness, autosomal recessive 86 (MIM: 614617). ‡, partially triplicated in 8/9 but significant uncertainty. Mouse data was from www.informatics.jax.org and www.mousephenotype.org.

Gene symbol	*TBC1D24*	*ATP6V0C*	*AMDHD2*	*CEMP1*	*PDPK1*
Full gene name	TBC1 domain family member 24	ATPase H+ transporting V0 subunit c	amidohydrolase domain containing 2	cementum protein 1	3-phosphoinositide dependent protein kinase 1
HGNC ID	29203	855	24262	32553	8816
MANE ID (Refseq)	NM_001199107.2	NM_001694.4	NM_001330449.2	NM_001048212.3	NM_002613.5
Exon number	8	3	11	1	14
Protein size (aa)	559	155	409	247	556
Genomic position (GRCh38)	16:2,475,051-2,509,560	16:2,513,952-2,520,218	16:2,520,357-2,531,422	16:2,530,035-2,531,417	16:2,537,979-2,603,188
MIM gene number	613577	108745	620864	611113	605213
MIM condition	Familial infantile myoclonic epilepsy (605021, AR)[5], developmental and epileptic encephalopathy 16 (615338, AR)[6], DOORS syndrome (220500, AR)[7], Epilepsy, rolandic, with paroxysmal exercise-induce dystonia and writer's cramp (608105, AR)[8]†	Epilepsy, early-onset, 3, with or without developmental delay (620465, AD)[9]	NA	NA	NA
PanelApp panels (assessed 29th September 2025)	Fetal anomalies (v6.86), DDG2P (v6.5), Monogenic hearing loss (v5.27), Early onset or syndromic epilepsy (v8.33), Intellectual disability (v9.99)	Severe microcephaly (v8.12), DDG2P (v6.5), Early onset or syndromic epilepsy (v8.33), Intellectual disability (v9.99)	NA	NA	NA
GTEx expression in cerebellum (median TPM)	28.27	643.3	24.48	NA	33.73

Number of families full gene is triplicated in current study	6/9	9/9	8/9	8/9	0/9‡
RNAseq data for Family 1 (FC and rank), Table S3	1.95 (3)	1.83 (1)	-	-	1.47 (15)
RNAseq data for Individual 2 (FC and rank), Table S4	1.59 (8)	1.95 (1)	1.83 (12)	-	-
Triplosensitivity score[10] (pTriplo, from Decipher)	0.23	0.48	0.56	0.09	0.99
pLI score	0	0.74	0	NA	1.00
Missense constraint (Z-score)	1.10	2.91	0.39	-1.11	2.13
Mouse data - from Mouse Genome Informatics (MGI) and International Mouse Phenotyping Consortium (IMPC)	Heterozygous knockout mice show normal growth, neurodevelopment, and hearing with normal macroscopic brain appearance. Their hippocampal and cortical neurons show abnormal growth.[11]<br><br>Homozygous knockout animals show perinatal or postnatal lethality (MGI).<br><br>Homozygous, but not heterozygous conditional knockout animals exhibited spontaneous tonic–clonic seizure and normal hearing with normal macroscopic brain appearance.[12,13]	Homozygous knockout animals show abnormal embryogenesis and embryonic lethality before implantation (MGI).	Heterozygous knockout mice show altered behavior and impaired glucose tolerance. Homozygous knockout animals show preweaning lethality (MGI / IMPC).	Nil	Mice with conditional knockout exhibit decreased cerebellar size and ataxia-like behavior, suggesting that pdpk1 may be critical for motor balance and coordination.[14]
Summary from RefSeq/GeneCards (www.genecards.org)	This gene encodes a protein with a conserved domain, referred to as the	This gene encodes a component of vacuolar ATPase (V-ATPase), a	Enables N-acetylglucosamine-6-phosphate deacetylase	Enables hydroxyapatite binding activity. Involved in several processes,	Enables 3-phosphoinositide-dependent protein kinase

	TBC domain, characteristic of proteins which interact with GTPases. TBC domain proteins may serve as GTPase-activating proteins for a particular group of GTPases, the Rab (Ras-related proteins in brain) small GTPases which are involved in the regulation of membrane trafficking. Mutations in this gene are associated with familial infantile myoclonic epilepsy. Alternative splicing results in multiple transcript variants. [provided by RefSeq, Feb 2011].	multisubunit enzyme that mediates acidification of eukaryotic intracellular organelles. V-ATPase dependent organelle acidification is necessary for such intracellular processes as protein sorting, zymogen activation, receptor-mediated endocytosis, and synaptic vesicle proton gradient generation. V-ATPase is composed of a cytosolic V1 domain and a transmembrane V0 domain. The V1 domain consists of three A and three B subunits, two G subunits plus the C, D, E, F, and H subunits. The V1 domain contains the ATP catalytic site. The V0 domain consists of five different subunits: a, c, c', c', and d. This gene encodes the V0 subunit c. Alternative splicing results in transcript variants. Pseudogenes have been identified on chromosomes 6 and 17. [provided by RefSeq, Nov 2010].	activity. Involved in negative regulation of UDP-N-acetylglucosamine biosynthetic process. Located in nucleus. [provided by Alliance of Genome Resources, Jun 2025]	including biomineral tissue development; cell population proliferation; and odontogenesis. Located in cytoplasm and nucleoplasm. [provided by Alliance of Genome Resources, Jun 2025]	activity; phospholipase activator activity; and phospholipase binding activity. Involved in several processes, including cell surface receptor signaling pathway; intracellular signaling cassette; and regulation of signal transduction. Acts upstream of or within intracellular signal transduction. Located in cell projection; cytosol; and plasma membrane. Implicated in prostate cancer. Biomarker of lung non-small cell carcinoma. [provided by Alliance of Genome Resources, Jun 2025]

Gene	GRCh38 position	Transcript	*P*-value	*P*-adjust	Z-score	FC	DUP/TRIP
ATP6V0C	chr16:2513951-2520218	NM_001694.4	8.67E-19	1.42E-13	7.90	1.827663	TRIP
RNPS1	chr16:2253119-2268126	NM_080594.4	3.74E-14	2.03E-09	7.26	1.453973	DUP
TBC1D24	chr16:2475126-2505730	NM_001199107.2	1.29E-14	1.06E-09	7.17	1.945310	TRIP
FAM234A	chr16:234820-266096	NM_032039.4	1.65E-11	4.49E-07	-7.09	0.558644	-
SNRNP48	chr6:7590197-7611967	NM_152551.4	6.91E-13	2.82E-08	6.64	1.958841	-
CCNF	chr16:2429446-2458854	NM_001761.3	6.70E-11	1.57E-06	5.96	1.802501	TRIP
E4F1	chr16:2223590-2235742	NM_004424.5	4.64E-10	7.58E-06	5.83	1.424050	DUP
TEDC2	chr16:2460108-2464963	NM_025108.3	1.89E-10	3.85E-06	5.19	2.042024	TRIP
ZNF709	chr19:12461183-12484816	NM_152601.4	3.71E-10	6.74E-06	5.14	2.770219	-
MZT2A	chr2:131483959-131492397	NM_001085365.2	9.93E-09	0.000148	5.12	2.027919	-
PKD1	chr16:2088707-2135898	NM_001009944.3	4.34E-08	0.000592	5.11	1.613284	DUP (exons 1-34)
RNASEH2A	chr19:12806583-12813640	NM_006397.3	4.25E-07	0.004958	-5.05	0.683020	-
TRAF7	chr16:2155781-2178129	NM_032271.3	2.51E-07	0.003160	4.92	1.283426	DUP
ABCA3	chr16:2275880-2340728	NM_001089.3	1.03E-11	3.38E-07	4.88	8.633826	TRIP (exon 1) and DUP (exons 2-33)
PDPK1	chr16:2538020-2603188	NM_002613.5	5.70E-07	0.006213	4.73	1.474269	TRIP (exons 1-7) and DUP (exons 8-14)
PTPRG	chr3:61561570-62297609	NM_002841.4	1.25E-05	0.106629	-4.71	0.558644	-
NAGLU	chr17:42536240-42544449	NM_000263.4	1.30E-05	0.106629	-4.60	0.586417	-
MED29	chr19:39391377-39400641	NM_017592.4	1.62E-06	0.016512	4.38	2.158456	-
PDIA6	chr2:10783390-10812785	NM_005742.4	6.55E-05	0.445690	-4.15	0.721965	-
ECI1	chr16:2239401-2251587	NM_001919.4	8.25E-06	0.074890	4.13	1.635804	DUP
ATP6V1C2	chr2:10721629-10785110	NM_001039362.2	8.04E-05	0.486908	-4.11	0.707107	-

Table S3: RNA-seq expression outliers for the proband (III-3) in Family 1 filtered for those with absolute Z-score > 4.0.

Of the 21 outliers shown, 15 genes were upregulated and of these 11 lay on chromosome 16p13.3 within the duplication/triplication. We note that *ATP6V0C2* and *ATP6V1C2* both encode subunits of the vacuolar H^+-ATPase and there exists the possibility of a regulatory feedback loop leading to low expression of *ATP6V1C2*.

Gene	GRCh38 coordinates	GeneID	*P*-value	*P*-adjust	Z-score	FC	DUP/TRIP
ATP6V0C	chr16:2513952-2520218	ENSG00000185883	1.97E-13	3.87E-08	6.86	1.945310	TRIP
NDUFB2	chr7:140696708-140706643	ENSG00000090266	4.89E-11	4.80E-06	-6.68	0.619854	
RNPS1	chr16:2253120-2268126	ENSG00000205937	3.50E-10	2.29E-05	6.01	1.366040	DUP
RPS2	chr16:1962058-1964826	ENSG00000140988	5.47E-10	2.68E-05	5.80	1.670176	DUP
TSC2	chr16:2047985-2089491	ENSG00000103197	9.93E-10	2.94E-05	5.86	1.366040	DUP
TEDC2	chr16:2460109-2464963	ENSG00000162062	1.05E-09	2.94E-05	5.76	1.681793	TRIP
CCNF	chr16:2429447-2458854	ENSG00000162063	8.20E-10	2.94E-05	5.81	1.558329	TRIP
TBC1D24	chr16:2475127-2505730	ENSG00000162065	3.74E-09	9.17E-05	5.39	1.591073	TRIP
SDHAF3	chr7:97117698-97181763	ENSG00000196636	2.54E-08	0.000555	-6.00	0.517632	
NDUFB10	chr16:1959538-1961975	ENSG00000140990	5.26E-08	0.001032	5.18	1.443929	DUP
TRAF7	chr16:2155782-2178129	ENSG00000131653	1.08E-07	0.001929	5.05	1.337928	DUP
AMDHD2	chr16:2520371-2531417	ENSG00000162066	1.90E-07	0.003108	4.68	1.827663	TRIP
RAD50	chr5:132556977-132646349	ENSG00000113522	2.28E-07	0.003438	-5.31	0.673617	
TBL3	chr16:1972053-1982929	ENSG00000183751	3.05E-07	0.004278	4.83	1.375542	DUP
SLFN13	chr17:35435096-35448766	ENSG00000154760	3.71E-07	0.004859	-5.55	0.417544	
E4F1	chr16:2223591-2235742	ENSG00000167967	5.73E-07	0.007024	4.63	1.394744	DUP
FRG1	chr4:189940872-189963192	ENSG00000109536	8.24E-07	0.009518	4.66	1.404445	
CPNE1	chr20:35626044-35664900	ENSG00000214078	8.80E-07	0.009594	-5.35	0.334482	
ECI1	chr16:2239402-2251587	ENSG00000167969	1.49E-06	0.015427	4.46	1.505247	DUP
GFER	chr16:1984193-1987749	ENSG00000127554	4.18E-06	0.041001	4.30	1.464086	DUP
PGP	chr16:2211593-2214840	ENSG00000184207	5.10E-06	0.047688	4.25	1.404445	DUP

Table S4: RNA-seq expression outliers for Individual 2 filtered for those with absolute Z-score > 4.0.

Results are those using the MANE select transcript annotations rather than the original Genecode annotations. Of the 21 dysregulated genes, 16 were upregulated and of these, 15 lay on chromosome 16p13.3 within the duplication-triplication.

References

1. Watts, L.M., Bunyan, D.J., Giacopuzzi, E., Walker, S., Gazdagh, G., Thomas, N.S., Straub, V., Childs, A.M., Forsyth, J., Vogt, J., et al. (2024). FILIP1-associated neuromuscular disorder and phenotypic blending due to paternal UPD6. Brain Commun *6*, fcae330. 10.1093/braincomms/fcae330.
2. Roos, A., van der Ven, P.F.M., Alrohaif, H., Kolbel, H., Heil, L., Della Marina, A., Weis, J., Assent, M., Beck-Wodl, S., Barresi, R., et al. (2023). Bi-allelic variants of FILIP1 cause congenital myopathy, dysmorphism and neurological defects. Brain *146*, 4200-4216. 10.1093/brain/awad152.
3. Yu, J., Szabo, A., Pagnamenta, A.T., Shalaby, A., Giacopuzzi, E., Taylor, J., Shears, D., Pontikos, N., Wright, G., Michaelides, M., et al. (2022). SVRare: discovering disease-causing structural variants in the 100K Genomes Project. medRxiv, 2021.2010.2015.21265069. 10.1101/2021.10.15.21265069.
4. Pagnamenta, A.T., Yu, J., Willis, T.A., Hashim, M., Seaby, E.G., Walker, S., Xian, J., Cheng, E.W.Y., Tavares, A.L.T., Forzano, F., et al. (2023). A Palindrome-Like Structure on 16p13.3 Is Associated with the Formation of Complex Structural Variations and SRRM2 Haploinsufficiency. Human Mutation *2023*, 6633248.
5. Falace, A., Filipello, F., La Padula, V., Vanni, N., Madia, F., De Pietri Tonelli, D., de Falco, F.A., Striano, P., Dagna Bricarelli, F., Minetti, C., et al. (2010). TBC1D24, an ARF6-interacting protein, is mutated in familial infantile myoclonic epilepsy. Am J Hum Genet *87*, 365-370. 10.1016/j.ajhg.2010.07.020.
6. Guven, A., and Tolun, A. (2013). TBC1D24 truncating mutation resulting in severe neurodegeneration. J Med Genet *50*, 199-202. 10.1136/jmedgenet-2012-101313.
7. Campeau, P.M., Kasperaviciute, D., Lu, J.T., Burrage, L.C., Kim, C., Hori, M., Powell, B.R., Stewart, F., Felix, T.M., van den Ende, J., et al. (2014). The genetic basis of DOORS syndrome: an exome-sequencing study. Lancet Neurol *13*, 44-58. 10.1016/S1474-4422(13)70265-5.
8. Luthy, K., Mei, D., Fischer, B., De Fusco, M., Swerts, J., Paesmans, J., Parrini, E., Lubarr, N., Meijer, I.A., Mackenzie, K.M., et al. (2019). TBC1D24-TLDc-related epilepsy exercise-induced dystonia: rescue by antioxidants in a disease model. Brain *142*, 2319-2335. 10.1093/brain/awz175.
9. Mattison, K.A., Tossing, G., Mulroe, F., Simmons, C., Butler, K.M., Schreiber, A., Alsadah, A., Neilson, D.E., Naess, K., Wedell, A., et al. (2023). ATP6V0C variants impair V-ATPase function causing a neurodevelopmental disorder often associated with epilepsy. Brain *146*, 1357-1372. 10.1093/brain/awac330.
10. Collins, R.L., Glessner, J.T., Porcu, E., Lepamets, M., Brandon, R., Lauricella, C., Han, L., Morley, T., Niestroj, L.-M., Ulirsch, J., et al. (2022). A cross-disorder dosage sensitivity map of the human genome. Cell *185*, 3041-3055.e3025. 10.1016/j.cell.2022.06.036.
11. Finelli, M.J., Aprile, D., Castroflorio, E., Jeans, A., Moschetta, M., Chessum, L., Degiacomi, M.T., Grasegger, J., Lupien-Meilleur, A., Bassett, A., et al. (2019). The epilepsy-associated protein TBC1D24 is required for normal development, survival and vesicle trafficking in mammalian neurons. Hum Mol Genet *28*, 584-597. 10.1093/hmg/ddy370.
12. Tona, R., Chen, W., Nakano, Y., Reyes, L.D., Petralia, R.S., Wang, Y.X., Starost, M.F., Wafa, T.T., Morell, R.J., Cravedi, K.D., et al. (2019). The phenotypic landscape of a Tbc1d24 mutant mouse includes convulsive seizures resembling human early infantile epileptic encephalopathy. Hum Mol Genet *28*, 1530-1547. 10.1093/hmg/ddy445.
13. Tona, R., Lopez, I.A., Fenollar-Ferrer, C., Faridi, R., Anselmi, C., Khan, A.A., Shahzad, M., Morell, R.J., Gu, S., Hoa, M., et al. (2020). Mouse Models of Human Pathogenic Variants of TBC1D24 Associated with Non-Syndromic Deafness DFNB86 and DFNA65 and Syndromes Involving Deafness. Genes (Basel) *11*. 10.3390/genes11101122.

14. Liu, R., Xu, M., Zhang, X.Y., Zhou, M.J., Zhou, B.Y., Qi, C., Song, B., Fan, Q., You, W.Y., Zhu, J.N., et al. (2020). PDK1 Regulates the Maintenance of Cell Body and the Development of Dendrites of Purkinje Cells by pS6 and PKCgamma. J Neurosci *40*, 5531-5548. 10.1523/JNEUROSCI.2496-19.2020.
