# Supplementary material for: Palindrome-mediated 16p13.3 triplications cause a recognizable neurodegenerative disorder with ataxia
Source: Am J Hum Genet. 2025 Dec 4;113(1):221–33. doi: 10.1016/j.ajhg.2025.11.011 (PMC12824621; doi:10.1016/j.ajhg.2025.11.011)
Supplement: Document S2. Article plus supplemental information [file mmc2.pdf]

REPORT

Palindrome-mediated 16p13.3 triplications cause a recognizable neurodegenerative disorder with ataxia

Graphical abstract

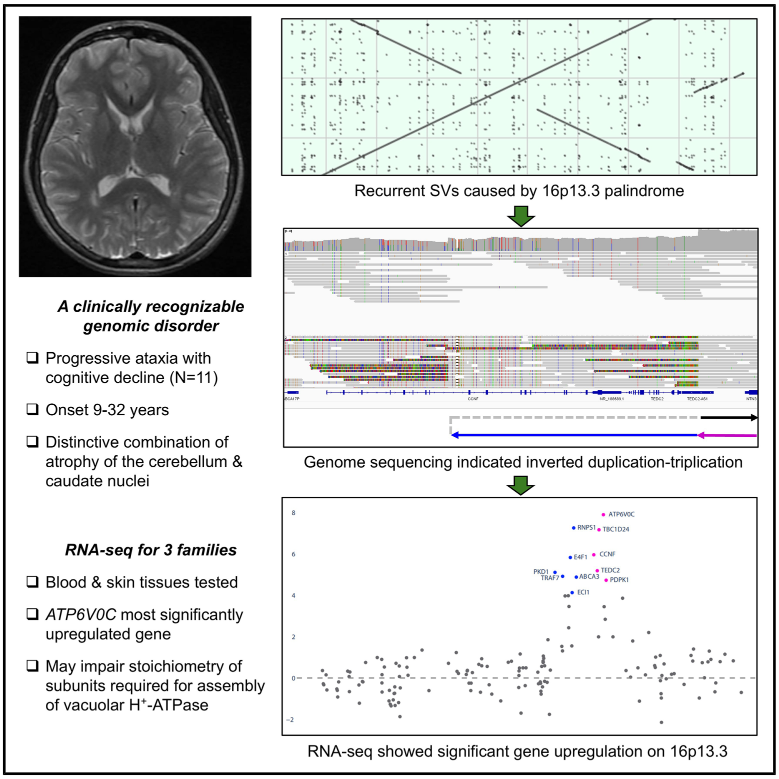

Authors

James Fasham, Julia Rankin, Rachel Schot, ..., Andrew H. Crosby, Emma L. Baple, Alistair T. Pagnamenta

Correspondence

e.baple@exeter.ac.uk (E.L.B.),
a.pagnamenta@exeter.ac.uk (A.T.P.)

Fasham and colleagues report a palindrome-mediated genomic disorder causing a recognizable, severe phenotype marked by early-onset progressive ataxia, cognitive decline, and cerebellar atrophy. Microarray and short/long-read genome sequencing uncovered overlapping inverted duplication-triplications on 16p13.3. RNA-seq showed upregulation of multiple genes within the variant, with *ATP6V0C* showing the most significant increase.

Fasham et al., 2026, The American Journal of Human Genetics *113*, 221–233

January 8, 2026 © 2025 The Authors. Published by Elsevier Inc. on behalf of American Society of Human Genetics.

https://doi.org/10.1016/j.ajhg.2025.11.011

Palindrome-mediated 16p13.3 triplications cause a recognizable neurodegenerative disorder with ataxia

James Fasham,[1,2,31] Julia Rankin,[2,31] Rachel Schot,[3] Susan M. White,[4,5] Katrina M. Bell,[4,6] Matthew N. Wakeling,[1] Lucy J. Mallin,[7] Alex Shah,[8] Michelle G. de Silva,[4,6] David I. Francis,[4] Maie Walsh,[9] Emily E. Jones,[10] Kayal Vijayakumar,[11] Katie Johnson,[12] Francis H. Sansbury,[13,14] Johann te Water Naudé,[15] Paola Giunti,[16] Marios Hadjivassiliou,[17] Andrea H. Nemeth,[18,19] George K. Tofaris,[18,20] Carlo Rinaldi,[20,21] Benito Banos-Pinero,[22] Marianna Selikhva,[23] Nishanka Ubeyratna,[1] Anneke Kievit,[3] Frank Sleutels,[3] Joey van Giessen,[3] Tahsin Stefan Barakat,[3] Timothy S. Hall,[1] Alan Whone,[24] Eleanor Thomas,[25] Joseph S. Leslie,[1] Rosemary A. Bamford,[1] Aaron R. Jeffries,[26] Jenny Lord,[27] Susan Walker,[28] Tjakko J. van Ham,[3] Sue L. Hill,[29] Lucy McGavin,[8,30] Andrew Parrish,[7] Andrew H. Crosby,[1] Emma L. Baple,[1,2,32,*] and Alistair T. Pagnamenta[1,32,*]

Summary

Complex neurodegenerative conditions have occasionally been associated with copy-number gains. Using microarray and genome sequencing on DNA samples from eleven individuals from nine unrelated families, we show that copy-number gains at 16p13.3 cause a severe, recognizable disorder characterized by early-onset progressive ataxia and cognitive decline (9–32 years). Most affected individuals also displayed peripheral neuropathy and scoliosis. Optic atrophy, nystagmus, and dystonia were more variable features. The neuroradiological phenotype comprises a distinctive combination of atrophy of the cerebellum and caudate nuclei. Co-segregation data showed that the structural variant (SV) had occurred *de novo* in 5 individuals and, for one other individual, had been inherited from a mosaic, unaffected parent. Triplicated segments of 16p13.3 were identified within the duplications. Although these varied in size (30–811 kb), the minimal region of overlap included a single gene (*ATP6V0C*) that is highly expressed in the cerebellum. RNA sequencing (RNA-seq) using whole-blood and fibroblast/lymphoblast cultures indicated increased expression of several genes within the SV, with *ATP6V0C* showing the most significant increase (up to 4-fold). In most cases, the central segment of the SV was proven to be inverted and lay immediately distal to a 144 kb palindrome. Across 500,000 individuals from the UK Biobank, we identified 19 duplications but no triplications at this locus. Further analysis of the consequences of *ATP6V0C* overexpression on the stoichiometry within the vacuolar H^+-ATPase heteromer and on neurological function will provide valuable pathomechanistic insights. Together, our findings define palindrome-mediated triplication on 16p13.3 as the cause of a clinically distinct childhood-onset neurodegenerative disorder.

Childhood-onset neurodegenerative disorders comprise a heterogeneous group of rare conditions that present with progressive neurological and/or cognitive impairment.[1] Features can include regression of motor or cognitive skills, seizures, behavioral changes, ataxia, spasticity, sensory deficits, and intellectual disability. Cerebellar atrophy is a frequent finding with a wide differential diagnosis, including mitochondrial disorders, such as Leigh

[1]Department of Clinical and Biomedical Sciences, Faculty of Health and Life Sciences, University of Exeter, EX2 5DW Exeter, UK; [2]Department of Clinical Genetics, Royal Devon University Hospital, EX1 2ED Exeter, UK; [3]Department of Clinical Genetics, Erasmus MC University Medical Center, Rotterdam, the Netherlands; [4]Victorian Clinical Genetics Services, Murdoch Children's Research Institute, Melbourne, Australia; [5]Department of Paediatrics, University of Melbourne, Parkville, VIC, Australia; [6]Murdoch Children's Research Institute, Melbourne, VIC, Australia; [7]Exeter Genomics Laboratory, Royal Devon University Healthcare NHS Foundation Trust, EX2 5DW Exeter, UK; [8]University Hospitals Plymouth NHS Trust, Derriford Road, Crownhill, Plymouth, PL6 8DH Devon, UK; [9]Genomic Medicine, the Royal Melbourne Hospital, Parkville, VIC, Australia; [10]Bristol Genetics Laboratory, North Bristol NHS Trust, BS10 5NB Bristol, UK; [11]Department of Paediatric Neurology, University Hospitals Bristol NHS Foundation Trust, BS1 3NU Bristol, UK; [12]Nottingham Regional Genetics Service, Nottingham City Hospital Campus, The Gables, NG5 1PB Nottingham, UK; [13]All Wales Medical Genomics Service, NHS Wales Cardiff and Vale University Health Board, Wales Genomic Health Centre, Cardiff Edge Business Park, Longwood Drive, Whitchurch, CF14 7YU Cardiff, UK; [14]Division of Cancer & Genetics, School of Medicine, Cardiff University, CF14 4XN Cardiff, UK; [15]Department of Paediatric Neurology, University Hospital of Wales, CF14 4XW Cardiff, UK; [16]Queen Square Institute of Neurology, WC1N 3BG London, UK; [17]Academic Department of Neurosciences, Sheffield Teaching Hospitals NHS Trust and University of Sheffield, S10 2JF Sheffield, UK; [18]Nuffield Department of Clinical Neurosciences, University of Oxford, OX3 9DU Oxford, UK; [19]Oxford Centre for Genomic Medicine, Oxford University Hospitals NHS Foundation Trust, OX3 7LD Oxford, UK; [20]Department of Clinical Neurology, Oxford University Hospitals NHS Foundation Trust, OX3 9DU Oxford, UK; [21]Institute of Developmental and Regenerative Medicine, University of Oxford, OX3 7TY Oxford, UK; [22]Oxford Genetics Laboratories, Oxford University Hospitals NHS Foundation Trust, OX3 9DU Oxford, UK; [23]Neurology Department, Southmead Hospital, BS10 5NB Bristol, UK; [24]Southmead Hospital, BS10 5NB Bristol, UK; [25]Department of Child Health, Royal Devon University Hospital, EX1 2ED Exeter, UK; [26]Biosciences, University of Exeter, EX1 2LU Exeter, UK; [27]Sheffield Institute for Translational Neuroscience (SITraN), University of Sheffield, S10 2HQ Sheffield, UK; [28]Genomics England, EC1M 6BQ London, UK; [29]NHS England, London, UK; [30]University of Plymouth, PL4 8AA Plymouth, UK
[31]These authors contributed equally
[32]These authors contributed equally
*Correspondence: e.baple@exeter.ac.uk (E.L.B.), a.pagnamenta@exeter.ac.uk (A.T.P.)
https://doi.org/10.1016/j.ajhg.2025.11.011.
© 2025 The Authors. Published by Elsevier Inc. on behalf of American Society of Human Genetics.
This is an open access article under the CC BY license (http://creativecommons.org/licenses/by/4.0/).

syndrome (MIM: 256000 and 500017),[2] ataxia telangiectasia (MIM: 208900),[3] disorders of neurodegeneration with brain iron accumulation (NBIA),[4] and others. These conditions are commonly autosomal-recessive or X-linked disorders, caused by loss of function genetic variants. Chromosomal gains involving triplosensitive genes are a rare cause of progressive childhood-onset neurological disorders. Notable examples include whole-gene duplication or triplication of *PLP1* (MIM: 300401) in Pelizaeus-Merzbacher disease (MIM: 312080)[5] and duplication or, rarely, triplication of *PMP22* (MIM: 601097) in Charcot-Marie-Tooth 1A (MIM: 118220).[6,7]

Technologies such as microarray and short-read genome sequencing (GS) are well suited to detecting microdeletions and microduplications but often fail to identify complex structures. In some cases, this can lead to diagnostic structural variants (SVs) being overlooked. Advances in computational algorithms for copy-number variant (CNV) and SV detection from GS and long-read sequencing now enable improved characterization of more complex chromosomal rearrangements. One notable example of such a rearrangement is the duplication-triplication. These configurations typically have an inverted central segment and are often mediated by oppositely oriented segmental duplications (SDs).[8] Complex SVs can also recur at palindromic DNA sequences, as these can promote double-strand breaks. Here, we used a combination of microarray, exome, GS, and RNA sequencing (RNA-seq) technologies to define palindrome-mediated duplication-triplications on 16p13.3 as the cause of a clinically and radiologically distinct early-onset neurodegenerative disorder with ataxia.

We initially identified individual 1, who presented at 10 years of age with childhood-onset, severe, and progressive ataxia, associated with axonal neuropathy and neuroradiological evidence of reduced cerebellar volume (Note S1). Array-based comparative genome hybridization (aCGH) analysis (8x60k constitutive v.3.0, Oxford Gene Technology) followed by parental segregation studies identified a rare *de novo* CN gain of 448 kb involving 16p13.3. The *de novo* nature of this variant prompted us to seek further individuals with similar chromosomal gains through international collaboration, using DECIPHER, GeneMatcher,[9] and the Genomics England National Genomic Research Library (NGRL; ethical approval from Cambridge South/Central RECs, 14/EE/1112, 20/EE/0035, and 25/EE/0125). Our analysis used Manta and Canvas calls for 100,000 Genomes Project (100kGP) participants, which were analyzed using SVRare.[10–12] For NHS Genomic Medicine Service (GMS) data, Dragen CNV calls were assessed with custom scripts. Genomic coordinates are reported using GRCh38.

In total, after resolving duplicate individuals identified across studies (Note S2), we ascertained 11 affected individuals from 9 distinct families (Figure 1A) with overlapping CN gains involving 16p13.3, progressive ataxia, and cerebellar atrophy. Phenotype information was obtained with informed consent by clinical care providers using a standardized proforma. Clinical findings for the 11 affected individuals are summarized in Table 1 and Note S1.

Affected individuals exhibited a shared phenotype characterized by progressive ataxia and cognitive decline. Ataxia, sometimes manifesting with dysarthria (10/11) and nystagmus (4/11), was usually noted in the second decade (range: 9–32 years). Mobility was typically affected, with a walking aid or wheelchair required in the third or fourth decade. Cognitive impairment was also universally present, with the majority (8/10) demonstrating progressive decline. This cognitive deterioration was often accompanied by behavioral challenges and sometimes required supportive care. In individuals 4, 7, and 8, either cognitive delay or ataxia predominated initially, with the other symptoms emerging later. Peripheral neuropathy, typically axonal and sensory predominant, was also common (9/11), as were absent deep tendon reflexes (9/10), *pes cavus* (5/6), and scoliosis/kyphosis (7/10). Additional, more variable neurological features, such as dystonia (2/10) and spasticity (1/9), were also observed. There was no craniofacial dysmorphism and no confirmed clinical or electrophysiological seizures reported. MRI neuroimaging findings were striking and consistent across all affected individuals, with cerebellar volume loss, caudate atrophy, and a high T2/FLAIR signal in the basal ganglia (Figures 1B–1I and S1). Progressive volume loss was also observed where multiple images were available for review (Figure 1E).

The pathogenicity of the overlapping gains identified in these 9 families is supported by inheritance studies (Figure 1A), with 5 confirmed *de novo* (inheritance not determined for 2 families). Family 1 comprised 3 affected individuals. A gain on 16p13.3 was identified through GS in the proband (III-3), and co-segregation studies showed the presence of the SV in her similarly affected brother (III-2) but not in the eldest unaffected sibling (III-1). The SV had been inherited from the affected mother (II-2), but as DNA was unavailable for the maternal grandfather, the inheritance for the mother is unknown.

Across the cohort, SV sizes ranged from 157 to 918 kb (Tables 1 and S1; Figure 2A). In all 7 families where short-read GS data were available (individuals 2 and 4–8 and family 1), the SV comprised a triplicated segment embedded within a duplication. As a representative example, IGV screenshots showing GS read alignments for individual 7 are shown in Figure 2B. Read alignments for other affected individuals are shown in Figures S2–S7. In all but one case (individual 5; Figure S4), split read pairs at the distal end (both mapping to the negative strand) suggest that the central portion of the rearrangement has been inverted. For individual 5, the duplication-triplication was also present in the unaffected father in a mosaic state (Figure S4), estimated to be heterozygous in 34%–37% of blood cells (Note S3).

Our systematic analysis of SVs in the 100kGP data strongly suggested that the presence of a 4-copy region is

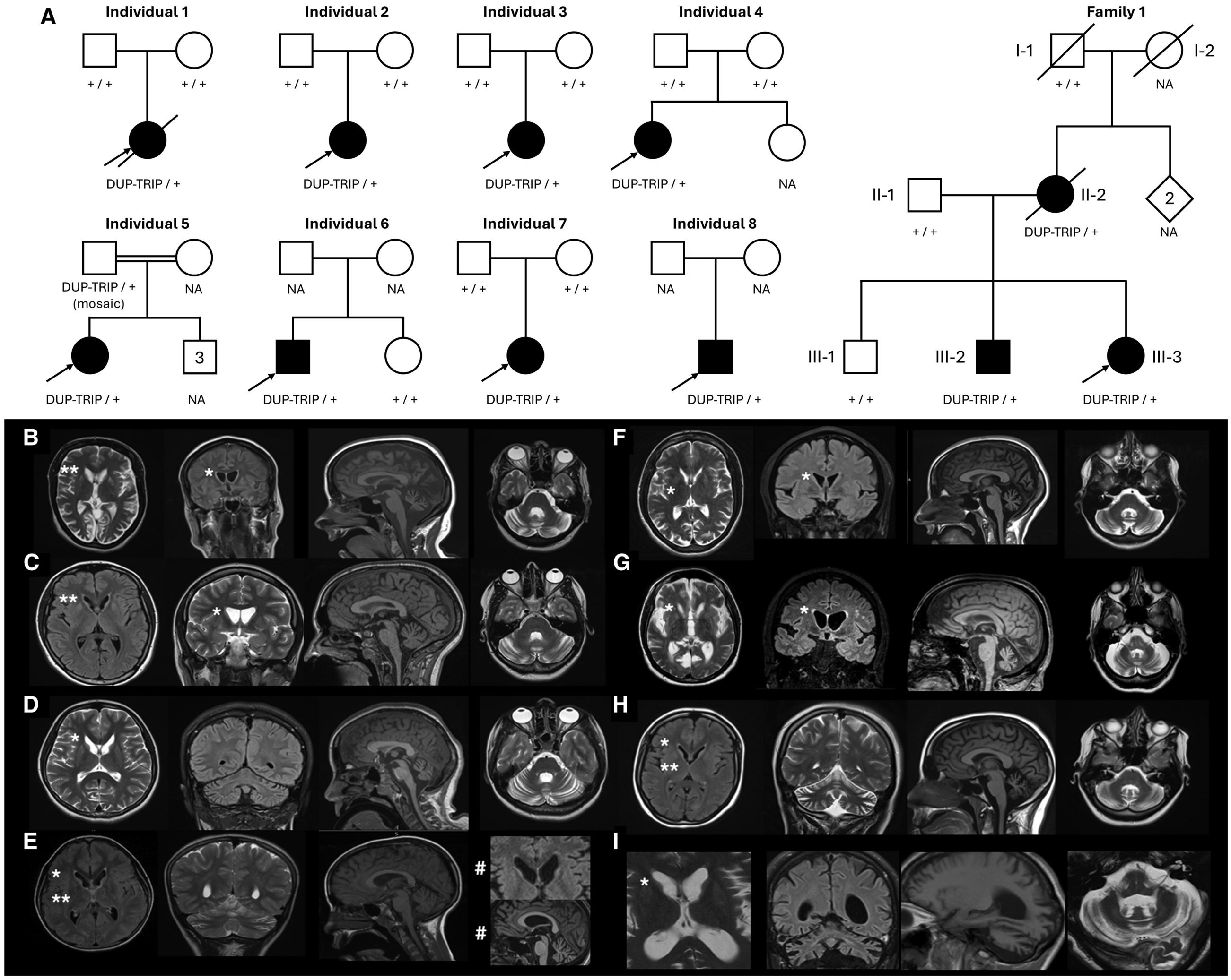

Figure 1. Family structures and MRI data for 11 individuals with overlapping gains on 16p13.3
(A) Pedigrees for 8 unrelated individuals and one multiplex family with overlapping SVs on 16p13.3. Individuals 1–3 were ascertained locally and via the DECIPHER database. Individuals 4–8 were identified from 100kGP and GMS data available in the National Genomic Research Library. Family 1 was ascertained via GeneMatcher, where *ATP6V0C* and *PDPK1* were entered as the candidate genes of interest. + represents the normal copy of 16p13.3.
(B–I) MRI scans from individuals 1–8 acquired at ages 36, 18, 9, 10 and 20 (#), 31, 53, 20, and 29. Axial and coronal T2 or FLAIR images demonstrate caudate atrophy of varying degrees (*) in all individuals and a high T2/FLAIR signal in the basal ganglia (**) in individuals 1, 2, 4, and 7. Sagittal T1 and axial T2 images (columns 3 and 4) show the cerebellar hemisphere and vermian atrophy in all individuals. There is progressive caudate and cerebellar atrophy in individual 4 (row E). For further MRI images, see Figure S1.

critical for the early-onset progressive ataxia phenotype (Note S4), and this was supported by the results for individuals 4–8 and family 1. This prompted closer scrutiny of the existing data for individuals 1–3. The datasets available for individuals 1 and 3 were limited to exome sequencing and aCGH. Lower-resolution aCGH data could not distinguish between duplicated and hypothetically triplicated regions, and data from a ~60,000-resolution array for individual 3 are shown as a representative example in Figure S8. In contrast, retrospective coverage analysis of the previously uninformative exome data was consistent with segments of triplication embedded within the respective duplications (Figure S9). For individual 2, repeat analysis using a higher-resolution array with ~1.8 M probes identified a nested region of triplication (Figure S10), undetected by the original SNP microarray. This was subsequently confirmed by short-read GS (Figure S2). In individual 7, multiplex ligation-dependent probe amplification testing prior to 100kGP recruitment yielded fold change (FC) results intermediate between CNs 3 and 4 (Figure S11). These data highlight the difficulties of using non-GS methodologies to distinguish between duplicated and triplicated segments of under 500 kb.

The triplicated segments within the 16p13.3 SVs varied in size from 30 to 811 kb across the 11 individuals reported here (Table 1). The minimal region of triplication overlap was shown to be 26.1 kb and defined by the distal breakpoint in individual 5 and the proximal breakpoint in individual 1 (g.2503226–2529370 [GenBank: NC_000016.10]; Figure 2B; Table S1). A single protein-coding gene, *ATP6V0C*

Table 1. Clinical and neuroradiological findings in individuals with overlapping duplication/triplications involving 16p13.3

Individual ID (DECIPHER ID)	Individual 1 (381641)	Individual 2 (339803)	Individual 3 (359492)	Individual 4 (341728)	Individual 5	Individual 6	Individual 7	Individual 8	Family 1, II-2	Family 1, III-2	Family 1, III-3
Age at last assessment	died at 41 y	27 y	17 y	20 y	40 y	65 y	36 y	40 y	died at 56 y	36 y	27 y
Ancestry[a]	British	Australian[b]	Sri Lankan	British	Pakistani	British	Irish	British	Dutch	Dutch	Dutch
Sex	female	female	female	female	female	male	female	male	female	male	female
SV detection method	array + ES	array + GS	array, ES, LR	array + GS	GS	GS	GS + LR	GS	array + GS	array	array + GS
Duplication size	448 kb	704 kb	640 kb	918 kb	230 kb	157 kb	187 kb	378 kb	514 kb	514 kb	514 kb
Triplication size	30 kb	179 kb	317 kb	811 kb	64 kb	85 kb	105 kb	296 kb	250 kb	250 kb	250 kb
Inheritance	*de novo*	*de novo*	*de novo*	*de novo*	father mosaic	NK	*de novo*	NK	NK[c]	maternal	maternal
**Clinical features**											
Cognitive impairment	yes (<10 y)	mild (5 y)	yes, special education	mild/Mod. with ADHD	yes	significant	moderate	yes, initially normal	yes	yes, special education	yes, special education
Regression (8/10)	yes (in 20s)	no	yes	no	NK	yes	yes	yes (16 y)	yes (9 y)	yes	yes
Progressive ataxia[d]	yes (10 y)	yes (13 y)	yes (9 y)	yes (15 y)	yes	yes (32 y)	yes	yes (23 y)	yes (9 y)	yes (12 y)	yes (12 y)
Dysarthria (10/11)	yes	yes	yes	no	yes	yes	yes	yes	yes	yes (15 y)	yes
Nystagmus (4/11)	no	no	no	yes (15 y)	no	yes	yes	probable	no	no	no
Axonal neuropathy (9/11)	sensory (17 y)	no	yes	no	sensory	sensory	yes	sensory	sensory (46 y)	sensory (15 y)	sensory (9 y)
Pes cavus (5/6)	yes	NK	yes	no	NK	yes	NK	yes	NK	NK	yes
Dystonia (2/10)	no	no	no	no	yes	no	yes	no	no	NK	no
Scoliosis/kyphosis (7/10)	no	scoliosis (10 y)	scoliosis	scoliosis (14 y)	scoliosis	no	NK	scoliosis	kyphosis	no	scoliosis
Optic atrophy (2)	NK	NK	NK	NK	NK	yes	NK	no	yes	NK	NK
Other features	seizures suspected	hearing loss	hypertonia	macrocephaly clinodactyly	cataract, CVI, dysphagia	seizures suspected	chorea	–	spasticity	–	hypertonia, strabismus, hearing loss
**Neuroradiology**											
Cerebellar and caudate atrophy (11/11)	yes (35 y, 36 y)	yes (18 y, 23 y)	yes (9 y)	progressive (10 y, 20 y)	yes (31 y)	yes (53 y)	yes (20 y)	yes (29 y)	yes	yes (15 y)	yes (9 y)
Other findings	–	–	–	syrinx T4–T9	–	global atrophy	–	cerebral volume loss	–	–	–

The age at which the phenotype was first recorded is shown in parentheses. CNV sizes are approximate, as in most cases, there remains some uncertainty about the precise coordinates, particularly at the proximal breakpoints. Full genomic coordinates are shown in Table S1. Abs, absent; ADHD, attention-deficit hyperactivity disorder; CVI, cortical visual impairment; ES, exome sequencing; GS, genome sequencing; LR, long-read genome sequencing; Mod., moderate; y, years; NK, not known.

[a]Ancestry is as per self-report.

[b]European-Australian.

[c]Presumed *de novo*; not paternally inherited and no DNA available from the deceased mother.

[d]Ataxia was diagnosed during childhood in most patients. In others, the diagnosis was later, although it is possible that childhood symptoms preceded any formal diagnosis.

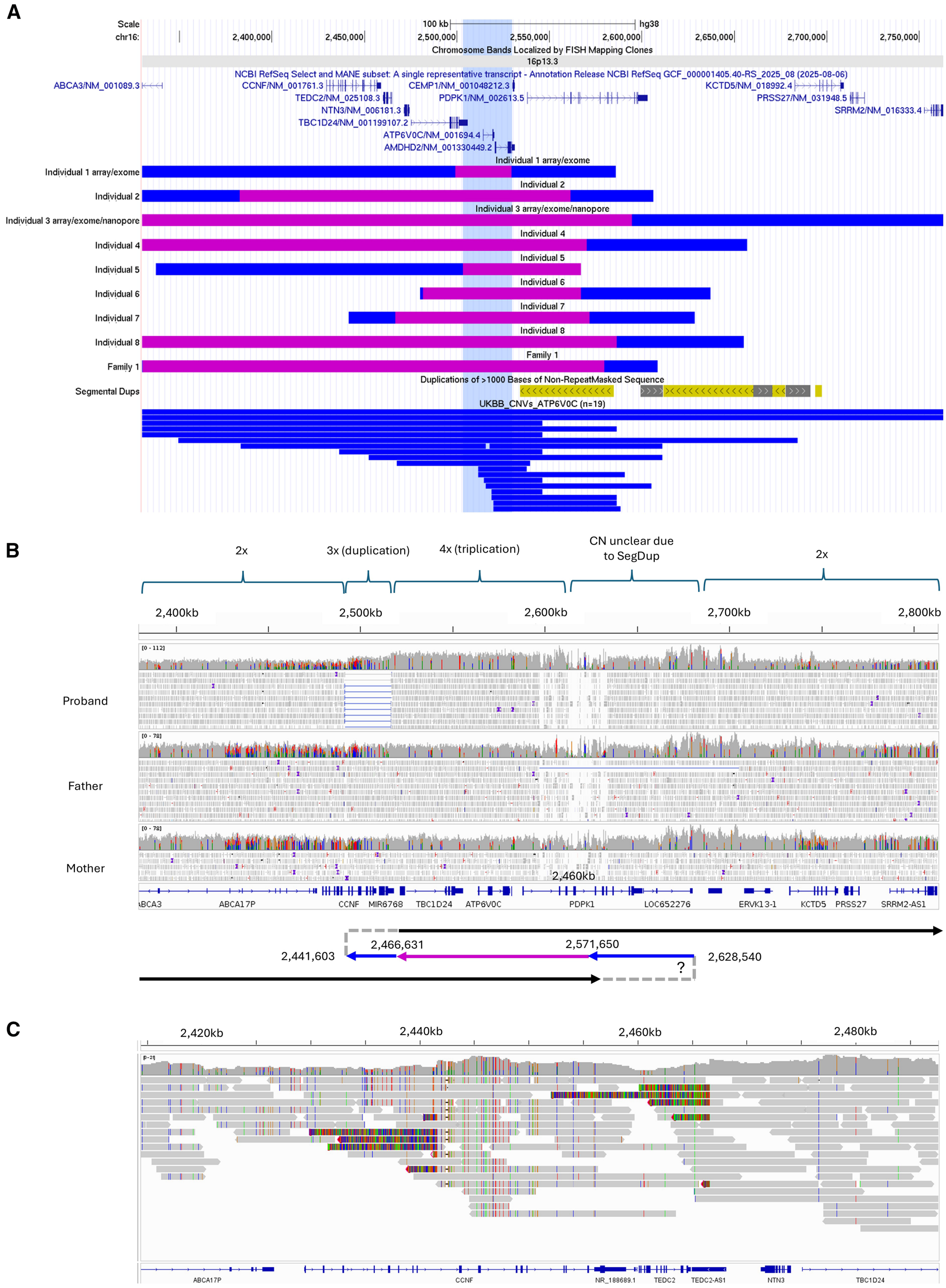

Figure 2. Genetic findings across 11 individuals with overlapping SVs on 16p13.3

(A) Custom UCSC browser session for the region chr16:2329790–2762907 showing relative positions of SVs across individuals 1–8 and family 1. Blue shading denotes duplication, and purple shading represents triplicated segments. For individuals 1 and 3, the array datasets available are of low resolution, so the presence of triplication is confirmed only by exome data and, hence, is approximate. The minimal region of triplication overlap is highlighted in light blue and overlaps a single protein-coding gene in its entirety, *ATP6V0C*. Segmental duplications are shown below the custom SV track and where yellow shading denotes similarity of 98%–99%. The bottom track shows 19 duplications identified in the UK Biobank that overlapped *ATP6V0C*. An interactive version of this image is available at https://genome.ucsc.edu/s/ExeterGenetics/16p13.3-TRIPv9.

(B) Short-read alignments shown in IGV that support the presence of the SV in individual 7, chosen as a representative example. The *x* axis coordinates correspond to the same region shown in (A) (coordinates lifted over to hg19). Regions of 3× and 4× are labeled, and

(legend continued on next page)

(MIM: 108745), lies fully within this region. *ATP6V0C* is highly expressed in the cerebellum, with a median "transcripts per million" (TPM) of 643.3 (GTEx data, accessed April 24, 2025). Other genes partially within or nearby the shared triplicated region include *TBC1D24* (MIM: 613577), *AMDHD2* (MIM: 620864), and *CEMP1* (MIM: 611113), and the evidence supporting the relative strength of candidacy for each of these is shown in Table S2.

Due to the presence of the 144 kb palindrome-like repeat, precise proximal breakpoint locations remain uncertain in most cases. The exception to this is the multiplex kindred (family 1), where, despite low mapping quality, split read pairs mapping to the positive strand helped resolve the proximal breakpoint junction (Figure S12). We next undertook PacBio HiFi GS for individual 7 using the Revio instrument, seeking to resolve more precisely the structure of these complex palindrome-mediated SVs. Analysis used the HiFi-human-whole-genome sequencing (WGS)-Workflow Description Language (WDL) pipeline (web resources). We obtained a mean coverage of 36× and validated the distal breakpoints that had previously been identified using short reads (Figure 2C). Although SV calling with HiFiCNV (v.1.0.0) and pbsv (v.2.9.0) identified a 120 kb duplication (g.2460001–2580000 [GenBank: NC_000016.10], imprecise) and a 25 kb inversion (g.2441607–2466627 [GenBank: NC_000016.10]) at the distal end, the proximal breakpoints remained algorithmically unresolved. Assigning read alignments into phase groups with HiPhase[13] and *de novo* assembly with hifiasm[14] was also unable to resolve the proximal end. However, by analyzing the phase of two *cis*-morphisms that are found 4.2 kb apart in the middle of the highly homologous repeat (Figure S13), we inferred approximate proximal breakpoint positions (Table S1). Similarly, we undertook nanopore sequencing for individual 3 according to the SQK-LSK110 protocol (Note S5) but obtained a mean genome-wide coverage of only ~5×. Although the distal breakpoint of the SV was identified (Figure S14), there were only four supporting reads, ranging from 849 bp to 35.9 kb in size. Due to higher per-base error rates with nanopore sequencing, *cis*-morphism analysis was not attempted, so the precise variant structure remains unclear.

To determine the likely transcriptional effect of these SVs, RNA-seq was undertaken for two affected individuals (III-3 in family 1 and individual 2), with consistent findings across these and a third already available RNA-seq dataset (described below). Fibroblast-derived RNA was utilized for family 1, III-3. Data analysis employed an adapted version of the OUTRIDER method described previously[15] and a transcript set based on RefSeq MANE v.1.3 (GRCh38). *Z* scores, FCs, and *p* values were calculated by comparing results with 133 control individuals, all unrelated patients for whom RNA-seq-based diagnostic testing had been requested at the Erasmus MC Clinical Genetics department. *ATP6V0C* was the most significantly dysregulated gene (Figures 3A and 3B), with a *Z* score of 7.90, an adjusted *p* value of 1.42×10^{-13}, and a FC of 1.83. Of the 15 upregulated genes with *Z* scores > 4 and $p < 0.0025$, 11 were localized within the 16p13.3 duplication/triplication (Table S3; Figure S15). RNA-seq data also identified a fusion transcript involving *PKD1* (MIM: 601313) and *ABCA3* (MIM: 601615) (Figures S16 and S17). However, given the variable distal breakpoints of overlapping SVs in this cohort, the fusion transcript's significance is unclear.

RNA-seq analysis for individual 2 used cultured lymphoblast-derived RNA. OUTRIDER analysis was performed across 114 samples sent in for RNA-seq-based diagnostics to identify genes with significant expression differences compared with the rest of the cohort. While the initial analysis used basic Gencode annotations and yielded non-significant results for *ATP6V0C* (FC = 2.10, *Z* score = 2.34), the results were confounded by Gencode annotations for fusion genes (Gencode: ENSG00000260272 and ENSG00000259784) that overlap the primary transcript for *ATP6V0C* (Gencode: ENST00000330398). Repeating this analysis using just the "MANE select" annotations, *ATP6V0C* became the most significantly upregulated gene (FC = 1.95, *Z* score = 6.86; Figures S18 and S19). Overall, 15/16 of the upregulated genes are from the inverted duplication-triplication region (Table S4). A split read pair with similar genomic coordinates to those seen in the GS data confirmed the distal breakpoint (Figure S2).

RNA-seq data were also available for individual 7 as part of the Genomics England transcriptomics study.[16] In brief, blood-derived RNA for 5,546 probands was collected using archived PaxGene tubes taken at recruitment to the 100kGP. RNA samples were depleted for rRNA/globin and sequenced using 100 bp paired-end reads. Alignment and transcript quantification were performed using Illumina's DRAGEN pipeline (v.3.8.4). Compared with the other 5,545 RNA-seq datasets and normalized to *ACTB* or *GAPDH*, the relative expression in this individual was 4.16–4.95× above the mean for *ATP6V0C* and 3.48–4.48× above the mean for *AMDHD2* (Figures 3C, 3D, and S20). Accurate measurement of *CEMP1* expression was not possible with short-read RNA-seq, as the gene is embedded within the 3′ UTR of *AMDHD2*.

To investigate the incidence of similar 16q13.3 rearrangements in the general population, we interrogated UK Biobank (UKB) data, which comprises ~500,000 UK

split read pairs (blue) are seen on the distal end of the SV, where both reads map to the negative strand and coincide with the stepped increases in coverage. The patchiness seen at the proximal end of the SV is due to reads with low mapping quality, shown in white. The purple/blue arrows in the subway plot denote that the central segment of the SV has been inverted. Other hypothetical conformations are shown in Figure 4.
(C) PacBio HiFi data for the same individual confirm the distal ends of the SV. Instead of split read pairs, the junction is visible due to soft-clipped bases present in a subset of reads. Data are shown according to the genome build GRCh38.

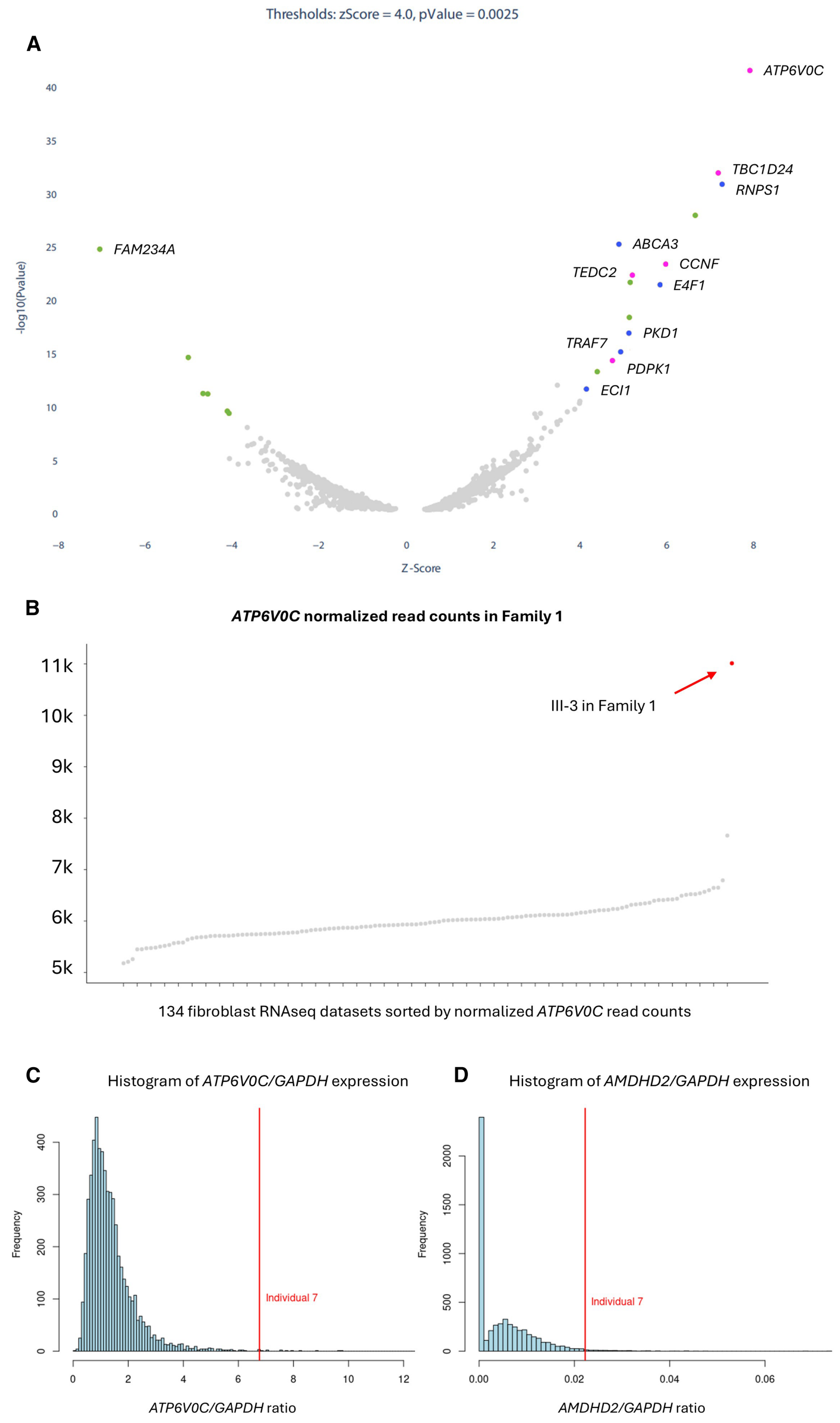

Figure 3. RNA-seq data for two individuals with 16p13.3 rearrangements confirm aberrant gene expression
(A) Genome-wide volcano plot showing genes that had significantly aberrant expression (Z score < -4 or > 4, $p < 0.0025$) in individual III-3 from family 1. Significantly upregulated genes that lie inside or outside the 16p13.3 duplication/triplication are highlighted in blue/purple or green, respectively. The most significant result was *ATP6V0C*, which lies inside the triplicated segment and has a Z score of 7.90. Although *FAM234A* also lies on 16p13.3, it is not in the duplicated region, and the aberrant expression detected was more

(legend continued on next page)

individuals between the ages of 40 and 69 years at recruitment. Of these, 490,640 participants underwent GS, as previously described.[17] CNVs were called using the DRAGEN v.3.7.8 CNV module, and data were searched using a custom RStudio script and a 10 kb size threshold. Nineteen individuals were identified harboring duplications of between 25.4 kb and 2.94 Mb that overlap *ATP6V0C* (Figure 2A). No triplications (i.e., a CN of 4) were identified, and segment CN ratios were 1.27–1.56. All of these individuals were healthy enough to be recruited to the UKB as adults. By reviewing hospital episode statistics, general practitioner (GP) records, and other self-reported clinical information, only one individual was identified who could plausibly be consistent with a milder form of the 16p13.3-triplication-linked condition, presenting with idiopathic familial dystonia and mild cognitive disorder (ICD-10 codes G24.1 and F06.7).

The clinical and genomic data described here define palindrome-mediated 16p13.3 triplication as the cause of a recognizable neurodegenerative disorder characterized by progressive ataxia and intellectual disability, with additional neurological features in many individuals. Cognitive impairment from childhood was universal and progressive in 80% of affected individuals. Notably, individual 1 developed severe, progressive dementia, raising the possibility of similar trajectories in other adult patients. Ataxia typically emerged during childhood or adolescence and progressed gradually. Peripheral neuropathy, spinal anomalies, and cerebellar signs, such as dysarthria and nystagmus, were frequent, though variably present. While some individuals had dystonia or spasticity, seizures were notably absent. Importantly, all patients shared a strikingly consistent MRI pattern, with cerebellar and caudate atrophy and basal ganglia signal abnormalities, supporting a shared, progressive neurodegenerative trajectory. This constellation of clinical and radiological features should prompt consideration of this disorder in undiagnosed individuals.

This condition is distinct among neurodegenerative ataxic disorders. While several conditions combine progressive ataxia with predominantly sensory axonal peripheral neuropathy (e.g., Friedreich ataxia [MIM: 229300],[18] ataxia with vitamin E deficiency [MIM: 277460], and CANVAS [MIM: 614575][19,20]), none are typically accompanied by progressive cognitive decline. Progressive intellectual impairment does occur in multisystemic conditions such as mitochondrial diseases and Refsum disease (MIM: 266500), but these typically also manifest outside the nervous system (e.g., as retinitis pigmentosa or cardiac impairment).

Although recent advances have improved our understanding of the genetic basis of rare hereditary ataxias and neurodegenerative disorders, current diagnostic yields remain below 50%,[21] suggesting that additional causative mechanisms remain to be identified. SVs, including copy gains, represent a class of genomic alterations that is technically challenging to detect and fully characterize and may account for a subset of undiagnosed individuals. Although CN gains have been found to cause complex neurodegenerative conditions, pathogenic triplications are relatively sparse in the literature. In many cases where triplication is a disease mechanism (*PLP1*, Pelizaeus-Merzbacher disease[22]; *SNCA*, Parkinson disease [MIM: 605543]; 17q22, retinitis pigmentosa [MIM: 600852][23]; and *APP*, Alzheimer disease [MIM: 104300][24]), the phenotype is more severe than the equivalent condition caused by duplication of the same locus. The neurodegenerative disorder described here contrasts with these, as there does not appear to be a consistent, milder ataxia/neurological phenotype in duplication carriers, supporting a higher dosage threshold for phenotypic expression. Based on this model, one would hypothesize that a relative CN of 4 due to a homozygous duplication would result in the same phenotype as a single triplicated allele. Alternatively, the mechanism may specifically depend on triplication.

Genomic palindromes can form hairpin or cruciform-type structures that promote double-strand breaks and lead to the formation of complex SVs. For instance, several inter/intra-chromosomal insertions into a 180 bp palindrome have been described ~80 kb downstream of *SOX3* (MIM: 313430). Depending on which sequences have been inserted, these can result in a wide range of phenotypes.[25–28] It is estimated that 718 palindromes > 200 bp are spread across the human genome.[29] Future studies should investigate these genomic loci for complex SVs that may also represent novel genomic disorders. There is likely considerable background of benign structural variation in the general population at the 16p13.3 locus due to the palindrome, and we note that GRCh38 and the CHM13 v.2.0 reference genomes differ substantially (Figure S21). Furthermore, the overall structure of inverted duplication-triplications is often ambiguous unless much longer sequencing reads or optical mapping data are available that span the central inverted segment (Figure 4). Resolving inverted duplication-triplication structures may become a key use case for long-read sequencing technologies as effective read lengths increase, and this locus, with its 33 kb segments of 99.8% sequence identity (Figure S22), is particularly challenging.

likely due to a rare intron 1 variant in the 5′ UTR (c.−140+2T>G [GenBank: NM_032039.4]). *Z* scores and *p* values were calculated by comparing results with 133 controls.
(B) Normalized read counts for *ATP6V0C* for all 134 fibroblast datasets confirm individual III-3 from family 1 to be the most significant outlier, with a relative fold change of 1.83. Similar RNA-seq results for individual 2 are presented in Figures S18 and S19.
(C and D) RNA-seq data from the 100kGP shows individual 7 to be an outlier for (C) *ATP6V0C* expression (4.95× above the mean) and (D) *AMDHD2* expression (4.48× above the mean), following normalization to *GAPDH*, when compared to 5,545 other RNA-seq datasets. Both genes lie within or partially within the triplicated segment that is shared across all 9 families. Data normalized to a second housekeeping gene are shown in Figure S20.

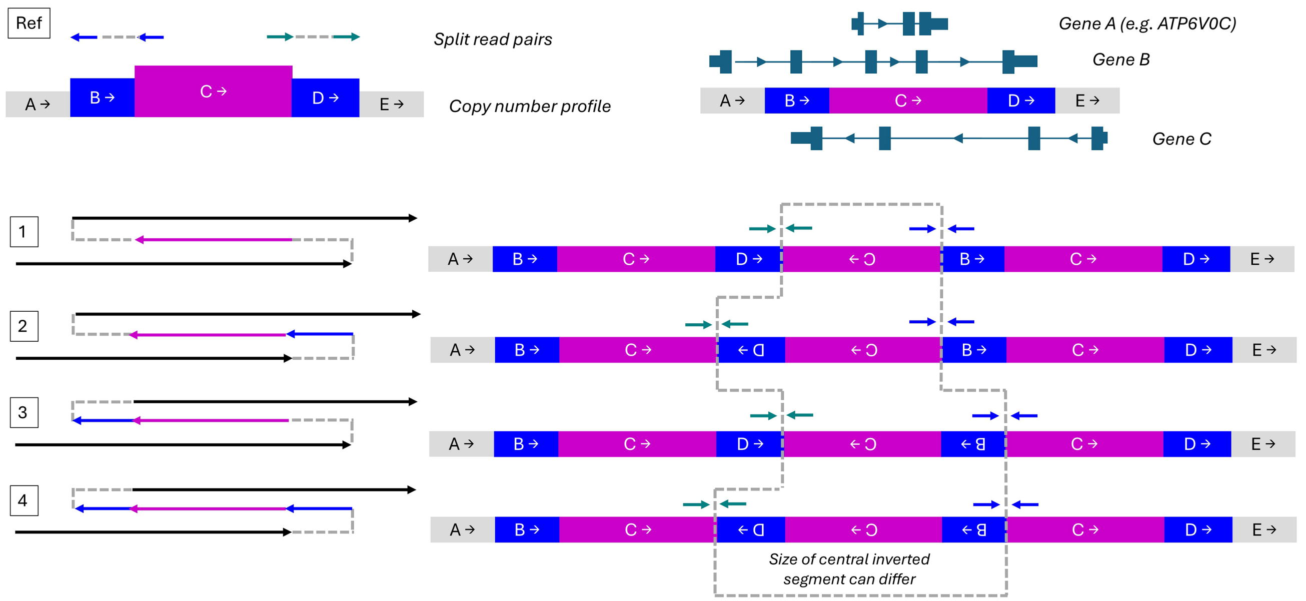

Figure 4. Schematic diagram showing the split-read pairs and copy number profile changes that are associated with inverted duplication-triplications

Although these types of rearrangements appear complex and can be called as 2 inversions, 2 duplications, and a central triplication, they involve just two breakpoints. The subway diagram highlights that there are typically 4 possible configurations that can all explain the short-read data. The size of the central inverted segment depends on which of these structures is true, and this can impact the interpretation. For instance, in this hypothetical example, the rearranged chromosome contains 3 copies of gene A (e.g., *ATP6V0C* in the present series of structural variants), irrespective of which precise structure is involved. In contrast, gene B is disrupted in solutions 2 and 4, while gene C would be disrupted in solutions 3 and 4. Ultra-long-read sequencing data or optical genome-mapping technologies where reads/labeled molecules span both breakpoint junctions can help distinguish which of the 4 possible solutions is right.

Using 100kGP data, we previously identified 3 individuals with intellectual disability and genetic haploinsufficiency of serine/arginine repetitive matrix 2 (*SRRM2*; MIM: 606032) resulting from complex deletion-inversions, with distal breakpoints clustered within the same 16p13.3 palindrome.[30] An additional *SRRM2* deletion-inversion identified from the GMS further supports the recurrent nature of complex SVs at this locus (Figure S23). Heterozygous loss-of-function variants in *SRRM2* (HGNC: 16639), a gene encoding a splicing factor, had previously been shown to result in autosomal-dominant intellectual developmental disorder type 72 (MIM: 620439).[31,32] The findings described here support our hypothesis that the symmetry of this 144 kb palindromic repeat would make the formation of complex SVs equally likely in both directions. In contrast to the proximal deletion-inversion events, where the molecular mechanism is *SRRM2* haploinsufficiency, RNA-seq has demonstrated a robust increase in expression for several genes within these more distal inverted duplication-triplications. Interpretation is limited by the fact that RNA data were available for only 3 of 11 individuals, from three different laboratories, and using non-disease-relevant tissues. Future studies assessing RNA and protein levels in disease-relevant cell types derived from postmortem tissues or differentiated induced pluripotent stem cells (iPSCs) would be valuable. It remains to be seen whether further instances of 16p13.3 triplication could be uncovered in genetically unsolved individuals with ataxia using an RNA-seq-first approach.

The identification of critical dosage-sensitive genes within disease-associated CNVs is challenging. Although several mechanistic questions remain, the most compelling candidate gene within the 16p13.3 SV interval is *ATP6V0C*. Deleterious variants in this gene have previously been linked to neurodevelopmental abnormalities, with epilepsy reported in several cases.[33,34] Microdeletions including this gene have also been implicated in neurodevelopmental disorders, with some sharing proximal breakpoints near the palindromic repeat.[35] *ATP6V0C* encodes the C subunit of the membrane-bound integral domain of a vacuolar proton pump enzyme complex, which acidifies organelles and establishes a proton gradient critical for several cellular processes. Pathogenic variants found in individuals with neurodevelopmental abnormalities were shown to impair V-ATPase function.[36] In family 1 and individual 2, RNA-seq indicated that the most significant gene expression changes occurred in *ATP6V0C*. We therefore speculate that the triplications described here may disturb the correct stoichiometry of subunits required for efficient assembly of the vacuolar H^+-ATPase. While a peripheral domain is involved with ATP hydrolysis, the integral membrane V0 domain mediates proton pumping via a rotary mechanism. This V0 domain contains a c-ring structure that rotates and is made up of 9 subunits encoded by *ATP6V0C* and just one encoded by *ATP6V0B*.[36] Future studies assessing how enzyme assembly is altered in cell lines from affected individuals and whether this leads to abnormal proton

gradients being established would provide valuable pathomechanistic insights.

Genes encoding other subunits of this large enzyme complex have also been linked to neurological disorders.[36] Notably, *de novo* variants in *ATP6V1A*, which encodes the A subunit, cause developmental encephalopathy with epilepsy.[37] More recently, variants in *ATP6V0A1* (which encodes the brain-enriched isoform of the a subunit in the V0 domain) have been shown to cause progressive myoclonus epilepsy and developmental and epileptic encephalopathy. Of relevance to the present study, 8 of 13 patients with *ATP6V0A1* variants presented with ataxia, and 25% had cerebellar atrophy.[38,39]

Another gene that lies within the region of triplication in all but one case is *CEMP1,* a single-exon gene embedded within the final exon of *AMDHD2*. This gene regulates the deposition of cementum that, together with the periodontal ligament, helps anchor teeth to the jawbone[40] and thus is not a strong functional candidate. Another candidate gene that lies proximal to *ATP6V0C*/*CEMP1* on 16p13.3 and is likely impacted by this SV is *PDPK1* (MIM: 605213). Based on an analysis of large rare CNVs in almost 1 M individuals,[41] *PDPK1* is predicted to be triplosensitive (pTriplo = 0.99). This gene encodes 3-phosphoinositide-dependent protein kinase 1 and is crucial for mammalian brain development. Mice with conditional knockout of the orthologous gene exhibit decreased cerebellar size and ataxia-like behavior, suggesting that *pdpk1* may be critical for motor balance and coordination.[42] GTEx data indicate that *PDPK1* has high relative expression in the cerebellum (a median TPM of 33.73). Although the gene does not fall within the minimal region of overlap, it must be noted that proximal breakpoints were imprecise due to the 144 kb palindromic repeat, and so the regions of triplication could potentially extend to include this gene in some cases. Even with the ~20 kb PacBio reads available for individual 7, proximal breakpoints of the rearrangement were unclear, and we hypothesized that they are situated between two *cis*-morphisms. Imprecise SV characterization is also pertinent to individuals 1 and 3 due to the low resolution of aCGH data, the low coverage of nanopore data, and the inability to obtain fresh samples, and the presence of a triplication was inferred from exome sequencing data.

While a simple dosage threshold model is an attractive option, we cannot rule out the occurrence of aberrant topologically associating domains (TADs) leading to ectopic gene expression. For other genomic disorders linked to specific SVs, 3D chromatin conformation using Hi-C and related methods has demonstrated how the altered distribution of TADs can impact gene expression.[43–45] One example is split-hand/foot malformation type 3 (MIM: 246560), where ~0.5 Mb duplications on 10q24 show a high degree of clustering of both start and endpoints.[46] This similarity is due to the precise enhancer repositioning, which mouse studies show is required to lead to the ectopic expression of *Lbx1* and *Btrc*.[43] The heterogeneity of sizes for the 16p13.3 SVs described here argues against a similar mechanism. However, future experimental studies using Hi-C should be complemented with deep learning computational methods[47] to confirm whether these complex genomic rearrangements affect chromatin structure and contribute to pathology.

We note that a complex rearrangement comprising 5 copies of *ATP6V0C* was recently described in a single individual presenting with ataxia.[48] The age of onset in this patient appears to have been earlier than in the 11 cases described in the present study (Table 1), with frequent falls and poor motor skills already noted by the age of 4. Additional features included amblyopia, hyperopia, dysarthric speech, coughing with liquid intake, excessive drooling, a progressive decrease in IQ, and cerebellar atrophy, which was first noted at 11 years (K. Hamanaka, personal communication). Future studies showing a correlation between higher CN states and disease severity would be of benefit for conclusively elucidating the pathomechanistic basis and clinical spectrum of this neurodegenerative disorder.

Together, our studies define a childhood-onset complex neurological disorder caused by overlapping 16p13.3 duplication-triplications and characterized by neurodegenerative features, including progressive ataxia, cognitive decline, and neuropathy, and a distinctive neuroradiological phenotype. Proximal breakpoints clustered within the 16p13.3 palindrome, making this the second complex genomic disorder linked to this mutational hotspot. Refinement of the spectrum of complex SVs that can lead to this disorder, combined with analyses of the consequences of increased *ATP6V0C* dosage on vacuolar H^+-ATPase formation and function, will be crucial to determining the underlying cause of disease and thus potential therapeutic interventions for this devastating disorder.

Data and code availability

Data for individuals 1–4 are available via the DECIPHER database (www.deciphergenomics.org). For individuals 4–8, GS data (including structural VCFs and gene-level SV reports) and transcriptomic data (for individual 7 only) are available in the NGRL v.5.1, Genomics England. Information about the NGRL can be found at https://doi.org/10.6084/m9.figshare.4530893.v7, and details of how researchers can apply for access can be found at www.genomicsengland.co.uk/join-us. For family 1, genome and RNA-seq data are stored in a repository at the Clinical Genetics Department of the Erasmus MC. The long-read GS data for individuals 3 and 7 and the exome sequencing data for individuals 1 and 3 are stored by the Exeter Genomics Laboratory. The consent agreements for these datasets only permit sharing anonymized data for affected individuals, and therefore, we are unable to share BAM or VCF files. The code to query SVs in the NGRL is available in the Genomics England Research Environment User Guide, found at https://re-docs.genomicsengland.co.uk/structural_variant. The other code used in this study has already been published.

Acknowledgments

First and foremost, we are grateful to the families in this study. We thank Jing Yu for sharing SVRare reports and Mark Nellist for useful discussions. This research was funded by the NHS Rare and Inherited Disease Genomic Network of Excellence and the National Institute for Health and Care Research (NIHR) Exeter Biomedical Research Centre (BRC). The views expressed are those of the authors and not necessarily those of the NIHR or the Department of Health and Social Care. This research was made possible through access to data in the NGRL, which is managed by Genomics England Limited (a wholly owned company of the Department of Health and Social Care). The NGRL is funded by the NIHR and NHS England. The Wellcome Trust, Cancer Research UK, and the Medical Research Council funded research infrastructure. This study makes use of data generated by the DECIPHER community. A full list of centers that contributed to the generation of the data is available from https://deciphergenomics.org/about/stats. DECIPHER is hosted by EMBL-EBI. DECIPHER project funding was provided by the Wellcome Trust (WT223718/Z/21/Z). This research was conducted using the UKB Resource under application number 103356 and used data provided by patients collected by the NHS as part of their care and support. UKB protocols were approved by the National Research Ethics Service Committee. The Barakat lab was supported by the Netherlands Organisation for Scientific Research (ZonMw Vidi, grant 09150172110002). The Rare Disease Flagship acknowledges financial support from the Royal Children's Hospital Foundation.

Author contributions

E.L.B., J.R., and A.H.C. conceived and supervised the study. L.M. analyzed the radiological images. E.L.B., A.H.C., R.S., T.S.B., S.M.W., and S.L.H. coordinated the collaborations. A.H.N., A.K., A.S., A.W., B.B.-P., C.R., E.L.B., E.T., F.H.S., G.K.T., J.F., J.R., J.t.W.N., K.J., K.V., M.G.d.S., M.H., M.S., M.W., P.G., and S.M.W. recruited the patients and/or assessed the clinical information. K.M.B., L.J.M., and T.S.H. provided bioinformatic support. D.I.F. and E.E.J. analyzed the array data. A.P. and A.R.J. supervised the GS analysis. R.A.B. and N.U. performed the genetic studies. A.T.P., F.S., J.F., J.L., J.S.L., J.v.G., M.N.W., R.S., S.W., and T.J.v.H. analyzed the genomic and/or transcriptomic datasets. A.T.P. and J.F. wrote the initial draft of the manuscript.

Declaration of interests

S.W. is an employee at Genomics England.

Declaration of generative AI and AI-assisted technologies in the writing process

During the preparation of this manuscript, the authors used ChatGPT (OpenAI) to assist with improving the grammar and clarity of some sentences. The authors subsequently reviewed and edited all content to ensure accuracy and take full responsibility for the final manuscript.

Supplemental information

Supplemental information can be found online at https://doi.org/10.1016/j.ajhg.2025.11.011.

Web resources

DECIPHER database, www.deciphergenomics.org
Gencode, https://www.gencodegenes.org
GeneMatcher, https://genematcher.org
GTEx, https://gtexportal.org
hifiasm, https://github.com/chhylp123/hifiasm
HiFi-human-WGS-WDL pipeline, https://github.com/PacificBiosciences/HiFi-human-WGS-WDL
HGNC, https://www.genenames.org
NCBI BLAST tool, https://blast.ncbi.nlm.nih.gov
OMIM, https://www.omim.org

Received: July 28, 2025
Accepted: November 12, 2025
Published: December 4, 2025

References

1. Wilson, D.M., 3rd, Cookson, M.R., Van Den Bosch, L., Zetterberg, H., Holtzman, D.M., and Dewachter, I. (2023). Hallmarks of neurodegenerative diseases. Cell *186*, 693–714. https://doi.org/10.1016/j.cell.2022.12.032.
2. Schubert Baldo, M., and Vilarinho, L. (2020). Molecular basis of Leigh syndrome: a current look. Orphanet J. Rare Dis. *15*, 31. https://doi.org/10.1186/s13023-020-1297-9.
3. Rothblum-Oviatt, C., Wright, J., Lefton-Greif, M.A., McGrath-Morrow, S.A., Crawford, T.O., and Lederman, H.M. (2016). Ataxia telangiectasia: a review. Orphanet J. Rare Dis. *11*, 159. https://doi.org/10.1186/s13023-016-0543-7.
4. Kurian, M.A., McNeill, A., Lin, J.P., and Maher, E.R. (2011). Childhood disorders of neurodegeneration with brain iron accumulation (NBIA). Dev. Med. Child Neurol. *53*, 394–404. https://doi.org/10.1111/j.1469-8749.2011.03955.x.
5. Wolf, N.I., Sistermans, E.A., Cundall, M., Hobson, G.M., Davis-Williams, A.P., Palmer, R., Stubbs, P., Davies, S., Endziniene, M., Wu, Y., et al. (2005). Three or more copies of the proteolipid protein gene PLP1 cause severe Pelizaeus-Merzbacher disease. Brain *128*, 743–751. https://doi.org/10.1093/brain/awh409.
6. Liu, P., Gelowani, V., Zhang, F., Drory, V.E., Ben-Shachar, S., Roney, E., Medeiros, A.C., Moore, R.J., DiVincenzo, C., Burnette, W.B., et al. (2014). Mechanism, prevalence, and more severe neuropathy phenotype of the Charcot-Marie-Tooth type 1A triplication. Am. J. Hum. Genet. *94*, 462–469. https://doi.org/10.1016/j.ajhg.2014.01.017.
7. Veitia, R.A., Zschocke, J., and Birchler, J.A. (2025). Gene Dosage Sensitivity and Human Genetic Diseases. J. Inherit. Metab. Dis. *48*, e70058. https://doi.org/10.1002/jimd.70058.
8. Carvalho, C.M.B., Ramocki, M.B., Pehlivan, D., Franco, L.M., Gonzaga-Jauregui, C., Fang, P., McCall, A., Pivnick, E.K., Hines-Dowell, S., Seaver, L.H., et al. (2011). Inverted genomic segments and complex triplication rearrangements are mediated by inverted repeats in the human genome. Nat. Genet. *43*, 1074–1081. https://doi.org/10.1038/ng.944.
9. Sobreira, N., Schiettecatte, F., Valle, D., and Hamosh, A. (2015). GeneMatcher: a matching tool for connecting investigators with an interest in the same gene. Hum. Mutat. *36*, 928–930. https://doi.org/10.1002/humu.22844.
10. Chen, X., Schulz-Trieglaff, O., Shaw, R., Barnes, B., Schlesinger, F., Källberg, M., Cox, A.J., Kruglyak, S., and Saunders, C.T. (2016). Manta: rapid detection of structural variants and

indels for germline and cancer sequencing applications. Bioinformatics *32*, 1220–1222. https://doi.org/10.1093/bioinformatics/btv710.
11. Roller, E., Ivakhno, S., Lee, S., Royce, T., and Tanner, S. (2016). Canvas: versatile and scalable detection of copy number variants. Bioinformatics *32*, 2375–2377. https://doi.org/10.1093/bioinformatics/btw163.
12. Yu, J., Szabo, A., Pagnamenta, A.T., Shalaby, A., Giacopuzzi, E., Taylor, J., Shears, D., Pontikos, N., Wright, G., Michaelides, M., et al. (2022). SVRare: discovering disease-causing structural variants in the 100K Genomes Project. medRxiv *2021*, 2010.2015–21265069. https://doi.org/10.1101/2021.10.15.21265069.
13. Holt, J.M., Saunders, C.T., Rowell, W.J., Kronenberg, Z., Wenger, A.M., and Eberle, M. (2024). HiPhase: jointly phasing small, structural, and tandem repeat variants from HiFi sequencing. Bioinformatics *40*, btae042. https://doi.org/10.1093/bioinformatics/btae042.
14. Cheng, H., Concepcion, G.T., Feng, X., Zhang, H., and Li, H. (2021). Haplotype-resolved de novo assembly using phased assembly graphs with hifiasm. Nat. Methods *18*, 170–175. https://doi.org/10.1038/s41592-020-01056-5.
15. Dekker, J., Schot, R., Bongaerts, M., de Valk, W.G., van Veghel-Plandsoen, M.M., Monfils, K., Douben, H., Elfferich, P., Kasteleijn, E., van Unen, L.M.A., et al. (2023). Web-accessible application for identifying pathogenic transcripts with RNA-seq: Increased sensitivity in diagnosis of neurodevelopmental disorders. Am. J. Hum. Genet. *110*, 251–272. https://doi.org/10.1016/j.ajhg.2022.12.015.
16. Pagnamenta, A.T., Yu, J., Walker, S., Noble, A.J., Lord, J., Dutta, P., Hashim, M., Camps, C., Green, H., Devaiah, S., et al. (2024). The impact of inversions across 33,924 families with rare disease from a national genome sequencing project. Am. J. Hum. Genet. *111*, 1140–1164. https://doi.org/10.1016/j.ajhg.2024.04.018.
17. UK Biobank Whole-Genome Sequencing Consortium (2025). Whole-genome sequencing of 490,640 UK Biobank participants. Nature *645*, 692–701. https://doi.org/10.1038/s41586-025-09272-9.
18. Delatycki, M.B., and Corben, L.A. (2012). Clinical features of Friedreich ataxia. J. Child Neurol. *27*, 1133–1137. https://doi.org/10.1177/0883073812448230.
19. Sullivan, R., Kaiyrzhanov, R., and Houlden, H. (2021). Cerebellar ataxia, neuropathy, vestibular areflexia syndrome: genetic and clinical insights. Curr. Opin. Neurol. *34*, 556–564. https://doi.org/10.1097/WCO.0000000000000961.
20. Szmulewicz, D.J., Roberts, L., McLean, C.A., MacDougall, H.G., Halmagyi, G.M., and Storey, E. (2016). Proposed diagnostic criteria for cerebellar ataxia with neuropathy and vestibular areflexia syndrome (CANVAS). Neurol. Clin. Pract. *6*, 61–68. https://doi.org/10.1212/CPJ.0000000000000215.
21. Meli, A., Montano, V., Palermo, G., Fogli, A., Rocchi, A., Gerfo, A.L., Maltomini, R., Cori, L., Siniscalchi, A., Bernardini, C., et al. (2025). Diagnosis of hereditary ataxias: a real-world single center experience. J. Neurol. *272*, 111. https://doi.org/10.1007/s00415-024-12772-9.
22. Bahrambeigi, V., Song, X., Sperle, K., Beck, C.R., Hijazi, H., Grochowski, C.M., Gu, S., Seeman, P., Woodward, K.J., Carvalho, C.M.B., et al. (2019). Distinct patterns of complex rearrangements and a mutational signature of microhomeology are frequently observed in PLP1 copy number gain structural variants. Genome Med. *11*, 80. https://doi.org/10.1186/s13073-019-0676-0.
23. de Bruijn, S.E., Fiorentino, A., Ottaviani, D., Fanucchi, S., Melo, U.S., Corral-Serrano, J.C., Mulders, T., Georgiou, M., Rivolta, C., Pontikos, N., et al. (2020). Structural Variants Create New Topological-Associated Domains and Ectopic Retinal Enhancer-Gene Contact in Dominant Retinitis Pigmentosa. Am. J. Hum. Genet. *107*, 802–814. https://doi.org/10.1016/j.ajhg.2020.09.002.
24. Grangeon, L., Cassinari, K., Rousseau, S., Croisile, B., Formaglio, M., Moreaud, O., Boutonnat, J., Le Meur, N., Miné, M., Coste, T., et al. (2021). Early-Onset Cerebral Amyloid Angiopathy and Alzheimer Disease Related to an APP Locus Triplication. Neurol. Genet. *7*, e609. https://doi.org/10.1212/NXG.0000000000000609.
25. Boschann, F., Moreno, D.A., Mensah, M.A., Sczakiel, H.L., Skipalova, K., Holtgrewe, M., Mundlos, S., and Fischer-Zirnsak, B. (2022). Xq27.1 palindrome mediated interchromosomal insertion likely causes familial congenital bilateral laryngeal abductor paralysis (Plott syndrome). J. Hum. Genet. *67*, 405–410. https://doi.org/10.1038/s10038-022-01018-z.
26. Bowl, M.R., Nesbit, M.A., Harding, B., Levy, E., Jefferson, A., Volpi, E., Rizzoti, K., Lovell-Badge, R., Schlessinger, D., Whyte, M.P., and Thakker, R.V. (2005). An interstitial deletion-insertion involving chromosomes 2p25.3 and Xq27.1, near SOX3, causes X-linked recessive hypoparathyroidism. J. Clin. Investig. *115*, 2822–2831. https://doi.org/10.1172/JCI24156.
27. Gardner, J.C., Jovanovic, K., Ottaviani, D., Melo, U.S., Jackson, J., Guarascio, R., Ziaka, K., Hau, K.L., Lane, A., Taylor, R.L., et al. (2025). Inter-chromosomal insertions at Xq27.1 associated with retinal dystrophy induce dysregulation of LINC00632 and CDR1as/ciRS-7. Am. J. Hum. Genet. *112*, 523–536. https://doi.org/10.1016/j.ajhg.2025.01.007.
28. Rahikkala, E., Komulainen-Ebrahim, J., Tolonen, J.P., Vorimo, S., Suo-Palosaari, M., Vieira, P., Piispala, J., Uusimaa, J., Pylkäs, K., and Mantere, T. (2024). Optical Genome Mapping Identifies a Second Xq27.1 Rearrangement Associated With Charcot-Marie-Tooth Neuropathy CMTX3. Mol. Genet. Genomic Med. *12*, e70014. https://doi.org/10.1002/mgg3.70014.
29. Ganapathiraju, M.K., Subramanian, S., Chaparala, S., and Karunakaran, K.B. (2020). A reference catalog of DNA palindromes in the human genome and their variations in 1000 Genomes. Hum. Genome Var. *7*, 40. https://doi.org/10.1038/s41439-020-00127-5.
30. Pagnamenta, A.T., Yu, J., Willis, T.A., Hashim, M., Seaby, E.G., Walker, S., Xian, J., Cheng, E.W.Y., Tavares, A.L.T., Forzano, F., et al. (2023). A Palindrome-Like Structure on 16p13.3 Is Associated with the Formation of Complex Structural Variations and SRRM2 Haploinsufficiency. Hum. Mutat. *2023*, 6633248.
31. Cuinat, S., Nizon, M., Isidor, B., Stegmann, A., van Jaarsveld, R.H., van Gassen, K.L., van der Smagt, J.J., Volker-Touw, C.M.L., Holwerda, S.J.B., Terhal, P.A., et al. (2022). Loss-of-function variants in SRRM2 cause a neurodevelopmental disorder. Genet. Med. *24*, 1774–1780. https://doi.org/10.1016/j.gim.2022.04.011.
32. Kaplanis, J., Samocha, K.E., Wiel, L., Zhang, Z., Arvai, K.J., Eberhardt, R.Y., Gallone, G., Lelieveld, S.H., Martin, H.C., McRae, J.F., et al. (2020). Evidence for 28 genetic disorders discovered by combining healthcare and research data. Nature *586*, 757–762. https://doi.org/10.1038/s41586-020-2832-5.

33. Rong, M., Marques, P.T., Ali, Q.Z., Morcos, R., Chandran, I., Qaiser, F., Møller, R.S., Bayat, A., Rubboli, G., Gardella, E., et al. (2025). Variants in ATP6V0C are associated with Dravet-like developmental and epileptic encephalopathy. Epilepsia *66*, 2046–2052. https://doi.org/10.1111/epi.18346.
34. Tinker, R.J., Burghel, G.J., Garg, S., Steggall, M., Cuvertino, S., and Banka, S. (2021). Haploinsufficiency of ATP6V0C possibly underlies 16p13.3 deletions that cause microcephaly, seizures, and neurodevelopmental disorder. Am. J. Med. Genet. *185*, 196–202. https://doi.org/10.1002/ajmg.a.61905.
35. Mucha, B.E., Banka, S., Ajeawung, N.F., Molidperee, S., Chen, G.G., Koenig, M.K., Adejumo, R.B., Till, M., Harbord, M., Perrier, R., et al. (2019). A new microdeletion syndrome involving TBC1D24, ATP6V0C, and PDPK1 causes epilepsy, microcephaly, and developmental delay. Genet. Med. *21*, 1058–1064. https://doi.org/10.1038/s41436-018-0290-3.
36. Mattison, K.A., Tossing, G., Mulroe, F., Simmons, C., Butler, K.M., Schreiber, A., Alsadah, A., Neilson, D.E., Naess, K., Wedell, A., et al. (2023). ATP6V0C variants impair V-ATPase function causing a neurodevelopmental disorder often associated with epilepsy. Brain *146*, 1357–1372. https://doi.org/10.1093/brain/awac330.
37. Fassio, A., Esposito, A., Kato, M., Saitsu, H., Mei, D., Marini, C., Conti, V., Nakashima, M., Okamoto, N., Olmez Turker, A., et al. (2018). De novo mutations of the ATP6V1A gene cause developmental encephalopathy with epilepsy. Brain *141*, 1703–1718. https://doi.org/10.1093/brain/awy092.
38. Aoto, K., Kato, M., Akita, T., Nakashima, M., Mutoh, H., Akasaka, N., Tohyama, J., Nomura, Y., Hoshino, K., Ago, Y., et al. (2021). ATP6V0A1 encoding the a1-subunit of the V0 domain of vacuolar H(+)-ATPases is essential for brain development in humans and mice. Nat. Commun. *12*, 2107. https://doi.org/10.1038/s41467-021-22389-5.
39. Bott, L.C., Forouhan, M., Lieto, M., Sala, A.J., Ellerington, R., Johnson, J.O., Speciale, A.A., Criscuolo, C., Filla, A., Chitayat, D., et al. (2021). Variants in ATP6V0A1 cause progressive myoclonus epilepsy and developmental and epileptic encephalopathy. Brain Commun. *3*, fcab245. https://doi.org/10.1093/braincomms/fcab245.
40. Campos, M.N., Giraldo, E.L., Del Rio Portilla, F., Fernández-Velasco, D.A., Arzate, H., and Romo-Arévalo, E. (2023). Solution NMR structure of cementum protein 1 derived peptide (CEMP1-p1) and its role in the mineralization process. J. Pept. Sci. *29*, e3494. https://doi.org/10.1002/psc.3494.
41. Collins, R.L., Glessner, J.T., Porcu, E., Lepamets, M., Brandon, R., Lauricella, C., Han, L., Morley, T., Niestroj, L.-M., Ulirsch, J., et al. (2022). A cross-disorder dosage sensitivity map of the human genome. Cell *185*, 3041–3055.e25. https://doi.org/10.1016/j.cell.2022.06.036.
42. Liu, R., Xu, M., Zhang, X.Y., Zhou, M.J., Zhou, B.Y., Qi, C., Song, B., Fan, Q., You, W.Y., Zhu, J.N., et al. (2020). PDK1 Regulates the Maintenance of Cell Body and the Development of Dendrites of Purkinje Cells by pS6 and PKCγ. J. Neurosci. *40*, 5531–5548. https://doi.org/10.1523/JNEUROSCI.2496-19.2020.
43. Cova, G., Glaser, J., Schöpflin, R., Prada-Medina, C.A., Ali, S., Franke, M., Falcone, R., Federer, M., Ponzi, E., Ficarella, R., et al. (2023). Combinatorial effects on gene expression at the Lbx1/Fgf8 locus resolve split-hand/foot malformation type 3. Nat. Commun. *14*, 1475. https://doi.org/10.1038/s41467-023-37057-z.
44. Dimartino, P., Zadorozhna, M., Yumiceba, V., Basile, A., Cani, I., Melo, U.S., Henck, J., Breur, M., Tonon, C., Lodi, R., et al. (2024). Structural Variants at the LMNB1 Locus: Deciphering Pathomechanisms in Autosomal Dominant Adult-Onset Demyelinating Leukodystrophy. Ann. Neurol. *96*, 855–870. https://doi.org/10.1002/ana.27038.
45. Nmezi, B., Rodriguez Bey, G., Oranburg, T.D., Dudnyk, K., Lardo, S.M., Herdman, N., Jacko, A., Rubio, S., Loeza-Alcocer, E., Kofler, J., et al. (2025). An oligodendrocyte silencer element underlies the pathogenic impact of lamin B1 structural variants. Nat. Commun. *16*, 1373. https://doi.org/10.1038/s41467-025-56378-9.
46. Holder-Espinasse, M., Jamsheer, A., Escande, F., Andrieux, J., Petit, F., Sowinska-Seidler, A., Socha, M., Jakubiuk-Tomaszuk, A., Gerard, M., Mathieu-Dramard, M., et al. (2019). Duplication of 10q24 locus: broadening the clinical and radiological spectrum. Eur. J. Hum. Genet. *27*, 525–534. https://doi.org/10.1038/s41431-018-0326-9.
47. Maroofian, R., Pagnamenta, A.T., Navabazam, A., Schwessinger, R., Roberts, H.E., Lopopolo, M., Dehghani, M., Vahidi Mehrjardi, M.Y., Haerian, A., Soltanianzadeh, M., et al. (2024). Familial severe skeletal Class II malocclusion with gingival hyperplasia caused by a complex structural rearrangement at the KCNJ2-KCNJ16 locus. HGG Adv. *5*, 100352. https://doi.org/10.1016/j.xhgg.2024.100352.
48. Hamanaka, K., Fujita, A., Miyatake, S., Misawa, K., Koshimizu, E., Uchiyama, Y., Tsuchida, N., Seyama, R., Sakamoto, M., Iwama, K., et al. (2025). Genome sequencing provides high diagnostic yield and new etiological insights for intellectual disability and developmental delay. NPJ Genom. Med. *10*, 60. https://doi.org/10.1038/s41525-025-00521-4.

The American Journal of Human Genetics, Volume *113*

Supplemental information

Palindrome-mediated 16p13.3 triplications cause a recognizable neurodegenerative disorder with ataxia

James Fasham, Julia Rankin, Rachel Schot, Susan M. White, Katrina M. Bell, Matthew N. Wakeling, Lucy J. Mallin, Alex Shah, Michelle G. de Silva, David I. Francis, Maie Walsh, Emily E. Jones, Kayal Vijayakumar, Katie Johnson, Francis H. Sansbury, Johann te Water Naudé, Paola Giunti, Marios Hadjivassiliou, Andrea H. Nemeth, George K. Tofaris, Carlo Rinaldi, Benito Banos-Pinero, Marianna Selikhva, Nishanka Ubeyratna, Anneke Kievit, Frank Sleutels, Joey van Giessen, Tahsin Stefan Barakat, Timothy S. Hall, Alan Whone, Eleanor Thomas, Joseph S. Leslie, Rosemary A. Bamford, Aaron R. Jeffries, Jenny Lord, Susan Walker, Tjakko J. van Ham, Sue L. Hill, Lucy McGavin, Andrew Parrish, Andrew H. Crosby, Emma L. Baple, and Alistair T. Pagnamenta

Note S1: Clinical case reports for affected individuals.

Individual 1

Individual 1 was the first child of unrelated parents. She had two healthy younger siblings and there was no family history of note. She was born at 34 weeks gestation and there were no reported complications during pregnancy or in the neonatal period. Early development was unremarkable, but her parents described her as a clumsy child. She attended a mainstream school with additional educational support in some areas (and a statement of special educational needs) but could read well. Slurred speech and walking difficulty were noted at age 10 and these progressed slowly over a number of years. Aged 14 she was able to walk half a mile but was unsteady.

Assessment by a pediatric neurologist at age 14 revealed truncal and limb ataxia, dysarthria, bilateral *pes cavus* and absent lower limb reflexes with equivocal plantar responses. Joint position and vibration sense were normal. Nystagmus and scoliosis were absent and there was no muscle weakness. Height was on the 75th centile.

Spinal MRI aged 13 was normal but brain MRI aged 16 showed marked cerebellar atrophy. At age 14 nerve conduction studies showed evidence of an axonal sensory neuropathy and at age 15 she underwent ambulatory EEG to investigate two possible seizures - daytime EEG was normal and nocturnal EEG showed possible predisposition to seizures but no seizure activity. Sodium Valproate treatment was started at this time and continued into adulthood, but no similar episodes were reported. The ataxia progressed and she started using a wheelchair in her 20s. Progressive dementia developed from her late 20s – at age 28 she could still read a book and play card games but when assessed at age 37 she had very limited understanding, aggressive behavior and was doubly incontinent without awareness of this. Her carers reported frequent 'blank' episodes lasting 10-15 minutes during which she was awake but would not respond in her usual manner. These were not investigated further.

On examination aged 37 she had coarse facial hair on her chin (present for at least 9 years), she was not dysmorphic, she was non-ambulant and in a wheelchair. Dysarthria and echolalia were noted, she had a full range of eye movements with no nystagmus but upgaze could not be elicited. Reflexes were absent and she had bilateral *pes cavus* with clawed toes. There was no evidence of muscle weakness, but cooperation with the examination was limited. Her mobility became progressively more severely impaired; she developed swallowing difficulties and died aged 42 years. MRI brain aged 35 showed marked parietal and cerebellar volume loss with caudate nuclei and putamina that were small with high T2 signal. Genetic testing for Friedreich Ataxia, Ataxia Telangiectasia, vitamin E deficiency and abetaliproteinaemia, SCA1 (*ATXN1*), SCA2 (*ATXN2*), SCA3 (*ATXN3*), SCA6 (*CACNA1A*), SCA7 (*ATXN7*), SCA17 (*TBP*) and Huntington's (*HTT*) were unremarkable. Gene agnostic trio exome analysis (Agilent SureSelect Human All Exon Kit v6 and sequencing on Illumina NextSeq) revealed no likely genetic diagnosis (a *de novo POLR2A* variant of uncertain significance was noted but thought unlikely to be deleterious and unlikely to explain her phenotype). Chromosome analysis revealed a normal female karyotype and plasma amino acids were normal apart from mildly elevated alanine which was not thought to be significant. Array CGH revealed a *de novo* 448kb duplication of chromosome 16p13.3.

Individual 2

Individual 2 is a 28 year old woman. She was born after an uneventful pregnancy to parents of Australia-European genetic ancestry. She has three older healthy siblings. Early developmental

milestones were normal but she was noted to have learning difficulties around age five years and was diagnosed with a mild intellectual disability. She completed her education with learning support. She developed a thoracolumbar scoliosis and was noted to have coordination difficulties, which were progressive.

An MRI of the brain aged 18 years showed cerebellar atrophy in cerebellar hemispheres and superior vermis. Volume loss was noted in the caudate nuclei and putamen bilaterally with associated T2/FLAIR hyperintensity. These findings were stable on repeat imaging at age 23. Growth at 19 years of age showed height on the 29th centile and weight on the 28th centile. No dysmorphic features were noted. She had an ataxic gait, with normal tone and power. Reflexes were difficult to elicit.

On reassessment at age 25 years, her ataxia had progressed. She was unsteady on her feet and had difficulties with fine motor tasks such as writing. Cognitively she continued to gain new skills, and her parents reported improved working memory. She did have fatigue in the afternoons requiring additional rest. She had some difficulty swallowing liquids. She was working as a volunteer and attending art classes. On examination, she had dysarthria, dysmetria and dysdiadochokinesis. She had an action tremor.

Individual 3

The female proband was the second child of three born to non-consanguineous parents of Sri Lankan genetic ancestry. The pregnancy was unremarkable, and she was born at term in good condition by *caesarean* section due to breech presentation with a birth weight of 2.4 kg. There were no neonatal concerns other than mild jaundice and there was normal attainment of early developmental milestones.

She started mainstream school in the UK at the age of 5. It was noted that she had some mild difficulties with learning and memory at this stage. At the age of 9 years, she started to experience recurrent trips and falls in addition to problems with fine motor skills at school. After physiotherapy input in the *community,* she was referred for further assessment. MRI brain showed cerebellar atrophy and volume loss. Electrophysiology studies showed chronic partial denervation in the tibialis anterior muscles. There was evidence of cerebellar ataxia (dysarthria and intention tremor). She also had bilateral *cavovarus* foot deformity and progressive scoliosis and absent reflexes.

Over a period of six years there was gradual deterioration in mobility resulting in use of a wheelchair. At the age of 16 years there was a significant deterioration in cognition and regression with severe dysarthria, dysphagia to solid food, weight loss and incontinence.

Individual 4

The patient is the second child of unrelated, healthy parents of British genetic ancestry. The reported family history is notable for neurodevelopmental conditions: her older sister has a diagnosis of ADHD (attention deficit hyperactivity disorder); her maternal cousin (daughter of her mother's sister) has polymicrogyria and learning difficulties; her maternal cousin (son of the same sister) has ADHD; and three of her paternal aunt's four sons all have learning difficulties.

She was born at term following an uncomplicated pregnancy and delivery, with a birth weight of 4.42 kg. She walked at 18 months and had no speech until the age of 2, after which she received speech and language therapy. At age 6-7 years, parental concerns prompted a referral to community paediatrics. At age 10, she exhibited poor attention span, clumsiness, and significant coordination difficulties. She required prompting to dress and assistance with showering and had no awareness of danger. Despite these challenges, she interacted well with peers and formed friendships. She

received diagnoses of ADHD and learning difficulties, both considered mild-to-moderate in severity. At age 13, she transitioned from mainstream education to a special needs school following a formal SEN (special educational needs) statement.

Although parents reported clumsiness, clinical assessments at ages 11 and 14 found no evidence of ataxia or cerebellar signs. By age 15, however, neurological symptoms had emerged, including frequent falls, mild oropharyngeal dysphagia, positive Romberg's sign, absent reflexes, and gaze-evoked nystagmus. Speech remained fluent. Examination at age 14 showed left-sided scoliosis (31°). There was no clinical evidence of peripheral neuropathy. Additional findings included bilateral fifth finger clinodactyly, mild constipation, and gastro-oesophageal reflux disease. Examination at age 20 showed mild heel-toe ataxia, mild bilateral nystagmus at extremes of gaze, mild bilateral intention tremor and mild slurred speech.

Growth parameters showed height consistently between the 50th and 75th centiles, and weight around the 75th centile. Occipitofrontal circumference (OFC) was on the 96th and 99.8th centiles on consecutive measurements. MRI scan aged 20 showed significant progression of cerebellar atrophy compared to the scan aged 20, as well as cerebellar volume loss.

Array CGH identified a *de novo* apparent duplication of 16p13. Trio genome sequencing, performed as part of the 100,000 genomes project (100kGP), confirmed this to be a *de novo* duplication-triplication of 16p13.3. The 100kGP also identified compound heterozygote *DHCR7* variants (P and VUS; NM_001360.3:c.452G>A, p.Trp151Ter and c.349T>A, p.Phe117Ile), but the patient does not have the associated phenotype of Smith-Lemli-Opitz syndrome. 7-DHC (7-dehydrocholesterol) levels were normal.

Individual 5

This 40-year-old woman, of Pakistani heritage, is the daughter of consanguineous parents and has three clinically unaffected brothers. She presents with cognitive impairment and progressive ataxia accompanied by dysarthria, dysphagia, dysdiadochokinesis, apraxia, and impaired distal proprioception. Eye movement examination revealed slow and hypometric saccades. There was mild sensory neuropathy and progressive scoliosis. Additional features include dystonia, recurrent cystitis, cataracts, cortical visual impairment, thyroid cancer, lymphadenopathy, and iron deficiency anemia. Trio genome sequencing, undertaken as part of the 100kGP, identified a duplication-triplication involving chromosome 16p13.3. This rearrangement was inherited from her clinically unaffected father, who is mosaic for the same variant.

Individual 6

This 65-year-old man is the son of non-consanguineous parents of British genetic ancestry and has a clinically unaffected sister. He first presented at age 39 with ataxia. Clinical history suggested that gait ataxia had been present for at least 7 years prior to presentation. He had normal developmental milestones and was still working as a gardener. Neurological examination revealed dysarthria and gaze evoked horizontal nystagmus. He had pale optic discs but normal visual acuity. Tone and power were normal but he was areflexic. He had bilateral *pes cavus* and blunting of vibration sensation. Neurophysiology showed a pure sensory neuropathy. Imaging showed cerebellar atrophy. Over time he developed significant cognitive and behavioral problems and his vision deteriorated. At the most recent assessment he was unable to mobilize outside of bed and exhibited features of advanced dementia. Trio genome sequencing, conducted through the 100kGP, identified a duplication-triplication involving chromosome 16p13.3.

Individual 7

This 36 year old woman was reported to be very clumsy as a child, but came to medical attention at age 20 for deteriorating balance which deteriorated over the next few years. She was noted at age 23 to have ataxia (unable to walk heel toe), dystonia affecting her neck but also more generalized on the left, and behavioral problems. There was no fundal pallor at that time. She had symptoms of autonomic disturbance with 'fainting' episodes, sweating and constipation. She had a postural drop of 30mmHg. She had numerous investigations, including a muscle biopsy which was suggestive of denervation. By age 29 she was fully dependent on others for care, and could only walk a few steps with support. Numerous genetic tests were negative at that time. Currently she is wheelchair bound due to ataxia, continues to have severe dystonia and has worsening behavioral and cognitive problems. Trio genome sequencing, undertaken as part of the 100kGP, identified a *de novo* duplication-triplication involving chromosome 16p13.

Individual 8

This 40-year-old man is the son of non-consanguineous parents of British genetic ancestry, with no relevant family history. Early development and cognition were normal. He completed formal education with good results, obtaining nine GCSEs. Scoliosis was noted during childhood. At age 12, he developed progressive ataxia that led to recurrent falls. This was accompanied by dysarthria, abnormal saccadic eye movements, absent reflexes on neurological examination and EMG -findings of subclinical sensory axonal neuropathy. The disease progressed slowly over time. By age 36, he required full-time use of a wheelchair and was no longer able to walk unassisted, even with walking aids. Following school, he experienced cognitive decline, including loss of numeracy skills, memory impairment, and an inability to write. His current mode of communication is limited to slurred, hypophonic single-word responses. Additional features include increased somnolence, hypersalivation, reduced dexterity, hypokinesia, and bradykinesia.

Neurological examination revealed intentional tremor on finger–nose testing, head drop, ptosis, impaired postural reflexes, and dystonic finger posturing. Laterocollis, hypometric saccades, broken pursuits, areflexia, *pes cavus*, and mild sensory neuropathy were also present. A left plantar extensor response was noted. Muscle tone remained normal, and muscle strength was preserved. There was no clinical evidence of optic atrophy. A dopamine transporter (DAT) scan performed in 2016 was positive. A trial of Madopar led to worsening paranoia and hallucinations, and he has since been maintained on neuroleptics for chronic psychosis. Extensive investigations, including copper studies, mitochondrial cytopathy screening, respiratory chain enzyme analysis, and genetic testing of the genes *POLG*, *MELAS*, *MERRF* and for common forms of spastic ataxia and spinocerebellar ataxia (SCA1, 2, 3, 6, 7, and 17), were uninformative. No mitochondrial DNA rearrangements or *FXN* GAA expansions were detected. Trio genome sequencing with a virtual panel for adult-onset neurodegenerative disorders also identified no pathogenic variants. Muscle biopsy showed non-specific muscle fibre shrinkage. Reanalysis of trio genome sequencing data through the NHS Genomic Medicine Service subsequently identified a duplication-triplication involving chromosome 16p13.3.

Family 1

II-2

This woman is the mother of two affected children (III-3 and III-4). Her own mother was deceased at the time of diagnosis, and her father has a normal neurological examination and does not carry the 16p13.3 duplication. Her clinically unaffected sisters have not undergone genetic testing for the

16p13.3 copy number variant. Early developmental milestones (walking and speech) were reported to be normal. She attended mainstream school and completed lower vocational education without obtaining a diploma. Neurological symptoms began at age 9, with ataxia, dysarthria, saccadic eye movements, absent reflexes, and intention tremor. At diagnosis (age 46), additional findings included axonal sensory neuropathy, prominent kyphosis, and optic atrophy. Over time, she developed joint contractures, progressive visual loss due to optic atrophy, and eventually unable to leave her bed became unresponsive prior to her death at age 56. An EEG at age 14 showed diffuse encephalopathy. Extensive investigations, including an ataxia gene panel (NGS/WES), metabolic screening, muscle biopsy, mitochondrial analysis, and *FMR1* repeat testing, were all uninformative. SNP array identified a duplication of 16p13.3, later clarified as a duplication-triplication using genome sequencing. This rearrangement was not paternally inherited. No maternal sample was available.

III-3

This is the affected son of II-2 and full brother to III-4. His parents are non-consanguineous; his father has mild intellectual disability. At birth, he required one day in an incubator due to aspiration of amniotic fluid. No other antenatal or perinatal concerns were noted. Motor milestones were delayed, with walking achieved at 26 months. He began in mainstream education but transferred to special education at age 9. Neurological symptoms began around age 12, including ataxia with dysarthria, saccadic eye movements, absent reflexes, and axonal sensory neuropathy. These signs progressed slowly. As of age 36, he is a full-time wheelchair user. A maternally inherited 16p13.3 duplication was identified on SNP array, and subsequent testing in other family members confirmed this to be a duplication-triplication.

III-4

This is the daughter of II-2 and full sister to III-3. Her parents are non-consanguineous; her father has mild intellectual disability. There were no concerns during pregnancy or birth. Development was delayed, and she began education in a specialist setting from age 5. She was later diagnosed with ADHD. At age 11, her total IQ was 65; by age 19, it had declined to 55. Her age at first walking and speech acquisition is unknown. At age 8, she developed ataxia with dysarthria, saccadic eye movements, absent reflexes, and intention tremor. Axonal sensory neuropathy was noted by age 9. These symptoms have progressed slowly, and she now predominantly requires a wheelchair to mobilize at age 27. Her height is 180 cm. An EEG at age 7 was normal. Metabolic and mitochondrial investigations were uninformative. SNP array identified a maternally inherited 16p13.3 duplication, later shown to be a duplication-triplication by genome sequencing. A healthy full brother of III-3 and III-4 has a normal neurological examination and does not carry the 16p13.3 rearrangement.

Note S2: Identification of individuals present across multiple datasets.

Individual 6 was identified in both the 100kGP and NHS GMS datasets, whilst Individual 4 was in DECIPHER and in 100kGP. Although the detection of the same ultra-rare variant in a different genomic database associated with the same phenotype can help validate disease associations, care has to be taken to avoid double-counting individuals who may have been recruited at different ages and thus with slightly different sets of clinical terms. A recent example of this was two sisters with biallelic inactivation of *FILIP1* who were tested in the 100kGP[1] and also in an independent exome sequencing study.[2] As the clinical information was collected at different time points, it was not immediately apparent that these could be the same individuals. In the present scenario, the unusual nature of the duplication-triplication made the similarity highly suspicious, as an identical

duplication-triplication was considered unlikely to have occurred independently. Both sample overlaps were confirmed by the recruiting clinicians.

Note S3: Estimation of mosaicism using read count information.

For Individual 5, the SV appeared to be present in the unaffected father in a mosaic state due to the presence of far fewer split reads visible in IGV (**Figure S4**). The stepped increase in read coverage was also not as distinct. To help support the presence of genetic mosaicism and estimate the fraction of cells affected, read-count information was generated using samtools (v1.16.1) to estimate the relative copy number ratios and thus the precise level of mosaicism. We used the 166 kb distally duplicated and the central 64 kb triplicated segments and normalized read counts to a non-overlapping 10 Mb region on 16p12-13 (GRCh38, 16:10,000,000-20,000,000). Compared to 303 other genomes sequenced as part of the same delivery date batch, read counts in the duplicated/triplicated regions in the proband were increased by a factor of 1.56/1.93. In the father, the increase was lower at 1.21/1.32. These data supported the evidence from split-reads indicating the unaffected father to be mosaic and the SV was estimated to be present in 34-37% of nucleated blood cells.

Note S4: Systematic analysis of CN3 and CN4 gains in the 100k Genomes Project.

To follow up the initial genetic findings (i.e. overlapping *de novo* gains) from Individuals 1-3, we analyzed Genome Sequencing data from the 100kGP and performed a systematic analysis of SVs that intersected *ATP6V0C*. This was done using SVRare and 554,060,126 aggregated SVs that had been called by Manta and Canvas, across 71,408 participants. These individuals were from the rare-disease arm of the project. SV aggregation was using an 80% overlap threshold and rare variants were defined as those where the aggregated SV was present in 1% of individuals or less, as described previously.[3]

Overall, we identified 39 SVs, of which 13 were gains, 15 were deletions and the remaining 11 were inversions. The 13 gains were observed in a total of 23 100kGP participants. Of these 23 individuals, 18 harbored gains where the copy number had been estimated to be 3 (CN3). Only one of these individuals was reported to have ataxia and had been recruited to the 100kGP with a clinical diagnosis of Charcot-Marie-Tooth disease. Although the phenotype appeared less severe, this individual had several features reminiscent of the condition described here (ataxia, sensorimotor neuropathy, *pes cavus*). The individual had been recruited to the 100kGP in their late 60s as singleton and so the inheritance of the gain was uncertain. Viewing read alignments with IGV (v2.18.2) confirmed the gain to be a 356 kb tandem duplication (chr16:2,316,840-2,672,796; GRCh38, **Figure S24**) with no sign of any internal 4 copy (CN4) segment.

For the remaining 5/23 individuals, the Canvas algorithm had assigned an estimated copy number of 4. Review of read alignment data in IGV suggested that one of these CN4 calls, 87kb in size (chr16:2,476,315-2,563,519; GRCh38), was a false positive as it was in a sample with extremely wavey coverage (**Figure S24**) and a genome-wide total of 4,875 Canvas calls. Of the 337 rare disease genome datasets in the same sequencing batch, this was the highest number of Canvas calls and represents a 4.6x increase compared to the mean number of Canvas calls across all genome datasets in that batch. The remaining 4 individuals with CN4 calls all had a strikingly similar combination of features which included progressive ataxia, cerebellar hypoplasia and cognitive decline. Manual review of read alignments (**Figure 2B, Figures S3-5**) identified stepped increases in read coverage and split read-pairs that supported these four Canvas calls. The four 100kGP participants harboring these SVs correspond to Individuals 4-7 reported here (**Note S1** and **Table 1**).

Given the uneven distribution of ataxia-related phenotypes associated with CN3 genotypes (1/18, relatively mild) versus the CN4 genotypes (4/4, all severe), this enrichment is consistent with data from the UK Biobank where no individuals harbored a CN4 event and only 1/20 of the CN3 carriers have ataxia. Taken together, this suggests that the severe form of the condition is specific to the CN4 rearrangements. Given the lack of parental samples to test inheritance, further studies would be required to confirm whether the CN3 change predisposes to a milder form of the condition and whether copy number states higher than CN4 can result in an earlier onset condition.

Note S5: Oxford Nanopore sequencing methods for Individual 3.

Library preparation was performed according to the SQK-LSK110 Oxford Nanopore Technologies protocol. Briefly, one microgram of DNA underwent repair and dA tailing (Cat No. E7180, New England Biolabs) followed by AmpureXP bead cleanup (Cat No. A63881, Beckmann Coultier). A ligation reaction was then performed to attach the adaptor, AmpureXP bead cleanup performed followed by QC measurements on a genomic tape (Cat No. 5067-5365, Agilent) and Qubit fluorometry (Cat No. Q33230, Fisher Scientific). 50 fmols of library was then loaded onto a MinION R9.4.1 flowcell and ran for 72h, with basecalling using the high-accuracy model via Guppy 5.0.16. Mapping to hg38 was with minimap2 version 2.17.

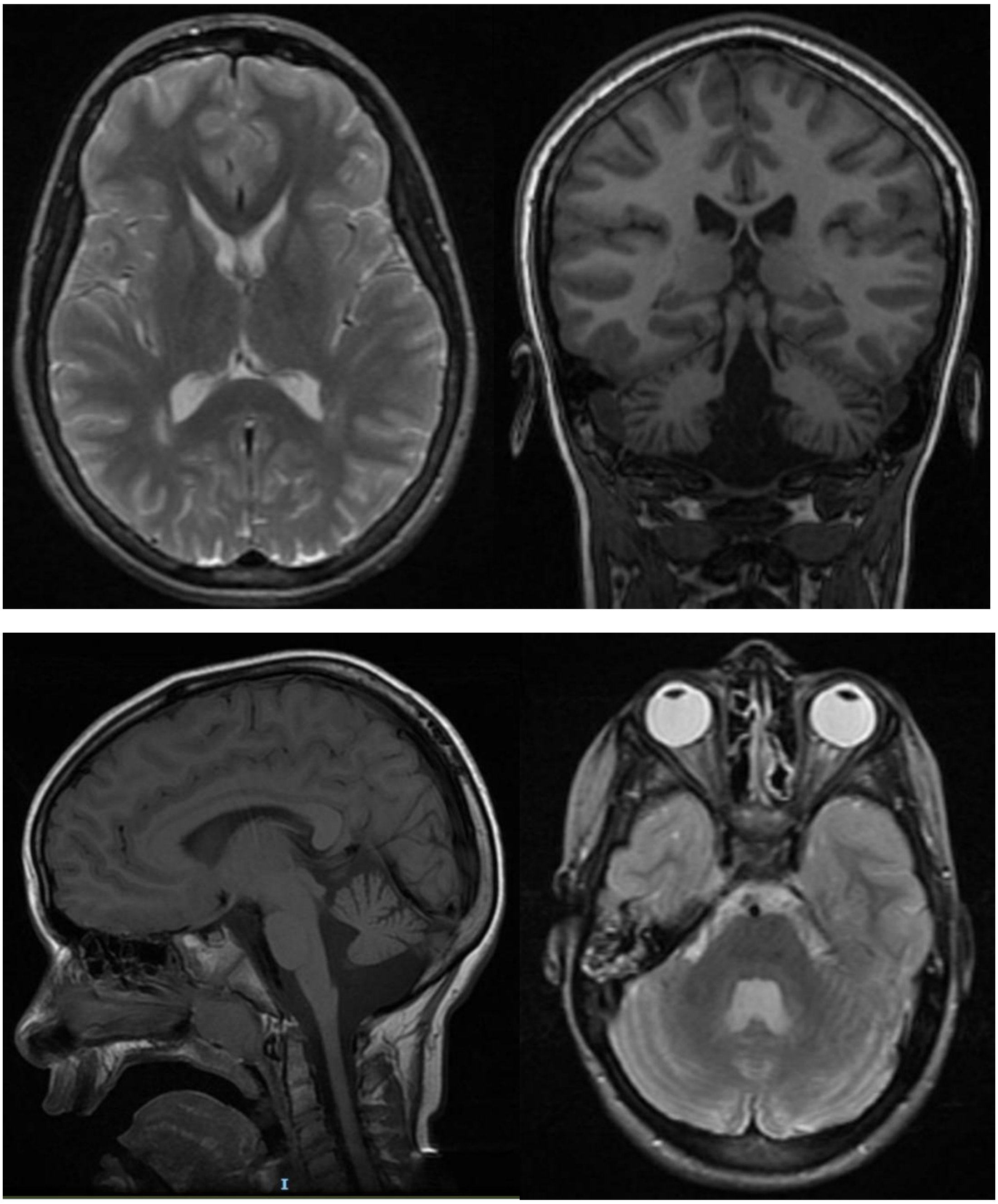

Figure S1: MRI scans from Family 1, Individual III-3.
Top row: Axial T2 weighted and Coronal 3D FSPGR (fast spoiled gradient echo) images demonstrate caudate atrophy and high T2 signal in the basal ganglia. Images acquired aged 12 years. Bottom row: Sagittal T1 and Axial T2 images show cerebellar hemisphere and vermian atrophy. Images acquired aged 9 years.

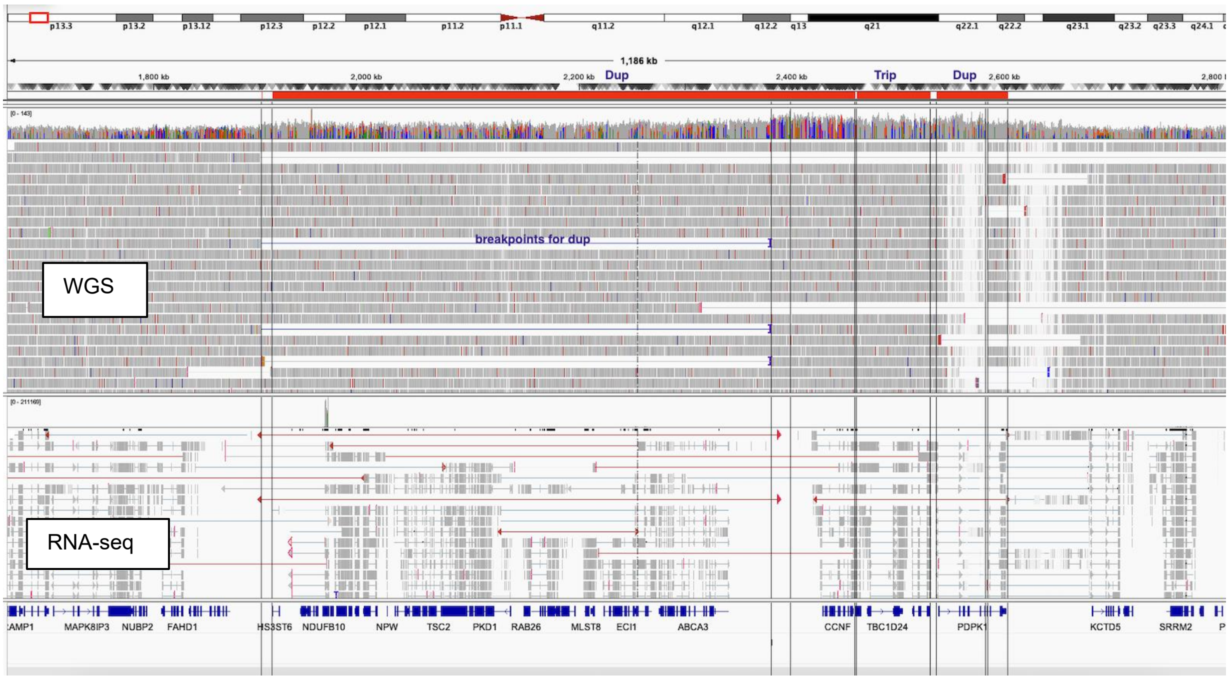

Figure S2: Read alignments from genome sequencing and RNA-seq data supporting a complex SV in Individual 2.

The distal end of the SV is shown by split read-pairs both mapping to the negative strand which are highlighted in blue. These breakpoints coincide with a stepped increase of read coverage from 2 to 3, then from 3 to 4 copies. Two similar read-pairs in the RNAseq data for this individual (lower track) also support the distal breakpoints. Red bars above the genome sequencing (GS) coverage track in IGV indicate the regions identified by microarray as dup/trip/dup. The GS data coverage therefore suggests that the distal duplicated segment is shorter and the triplicated segment larger than was documented by the microarray (**Figure S10**). Proximal breakpoints are likely to lie in the repeat regions where reads with low quality mapping are shown in white.

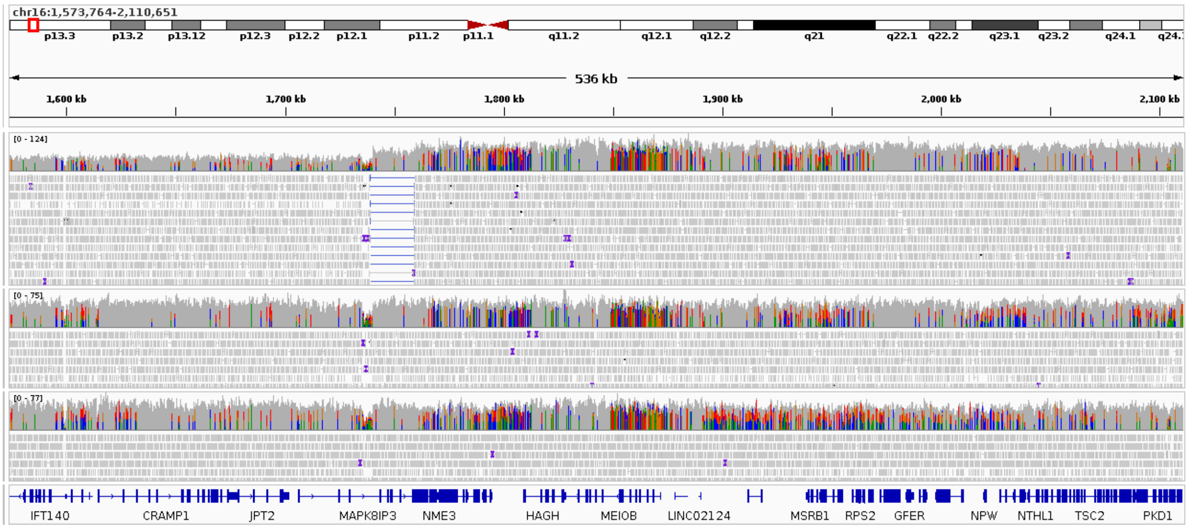

Figure S3: Read alignments from genome sequencing data supporting SV in Individual 4.

The distal end of the SV is shown for the proband (upper), where split read-pairs both mapping to the negative strand are highlighted in blue. These breakpoints coincide with a stepped increase of read coverage from 2 to 3, then from 3 to 4 copies. 150 bp reads are viewed in IGV using “collapsed” and

“view as pairs” settings. Parental data, shown in the bottom two tracks, indicate this SV to have arisen *de novo*.

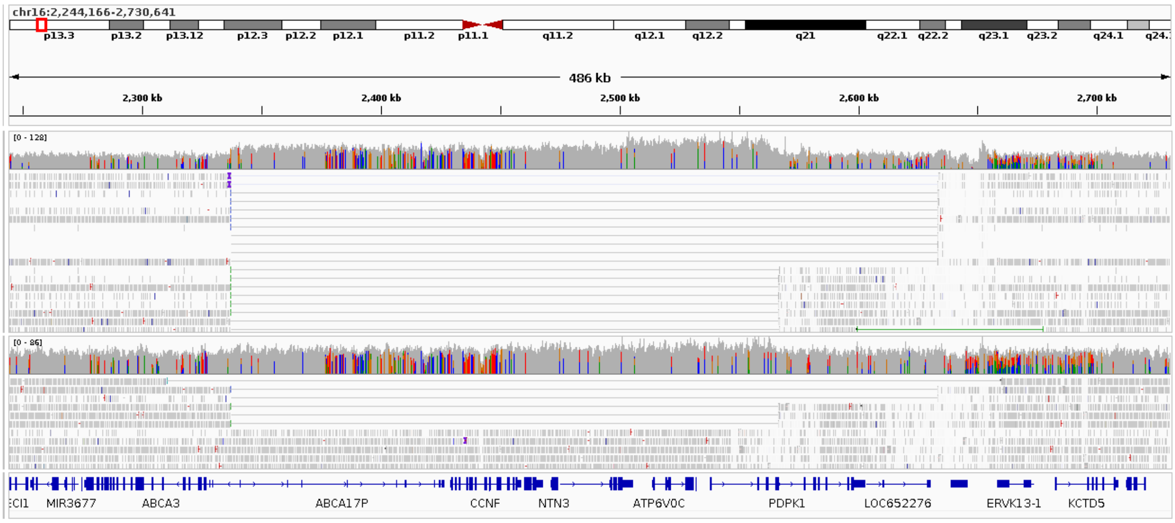

Figure S4: Read alignments from genome sequencing data supporting SV in Individual 5.

The full SV is shown for the proband (upper), where split read-pairs mapping from the start of the duplication to the proximal ends of the SV. The distal breakpoints coincide with a stepped increase of read coverage from 2 to 3, then decreased from 4 to 3 copies and from 3 to 2 copies. In contrast to the other SVs with genome sequencing data, the SV does not show clear negative to negative strand split read-pairs defining the distal end and so likely is not consistent with the Carvalho type structure. Nevertheless as the structure could not be resolved, the possibility of an inversion remains. 150 bp reads are viewed in IGV using “collapsed” and “view as pairs” settings. Paternal data (bottom track), shows a lower degree of increase in coverage and the similar split read-pairs are in a fewer percentage of reads, suggesting this individual to be mosaic for the variant.

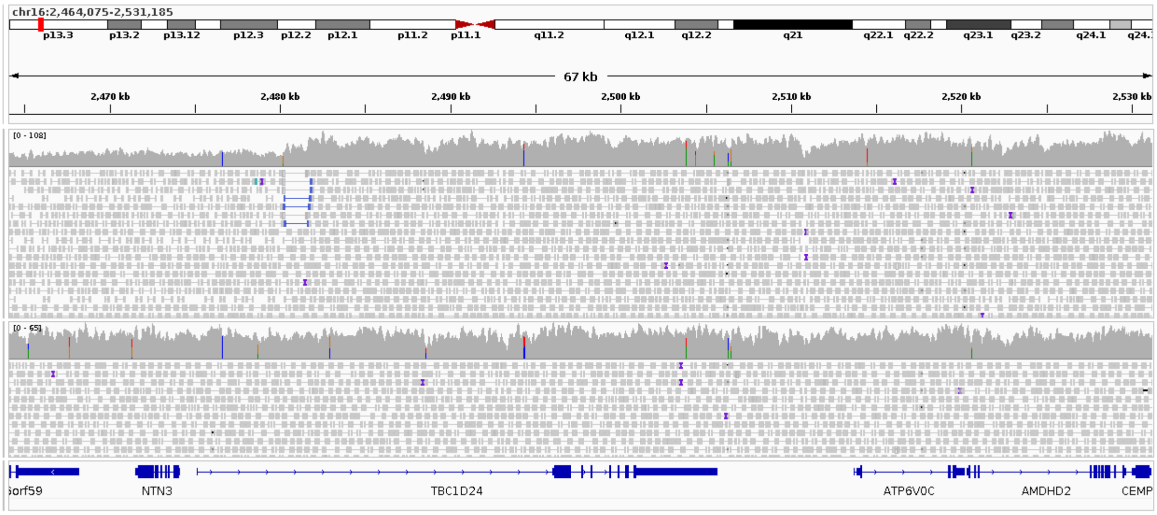

Figure S5: Read alignments supporting SV in Individual 6.

The distal end of the SV is shown for the proband (upper), where split read-pairs both mapping to the negative strand are highlighted in blue. These breakpoints coincide with a stepped increase of read coverage from 2 to 3, then from 3 to 4 copies and lie in the large first intron of *TBC1D24*. 150 bp

reads are viewed in IGV using “collapsed” and “view as pairs” settings. Genome sequencing data for the unaffected sister is shown in the bottom track indicate this individual has not inherited the variant. Although this individual was also sequenced as part of the NHS Genomic Medicine Service (GMS), which confirmed the SV, the data shown above is from the 100k Genomes Project.

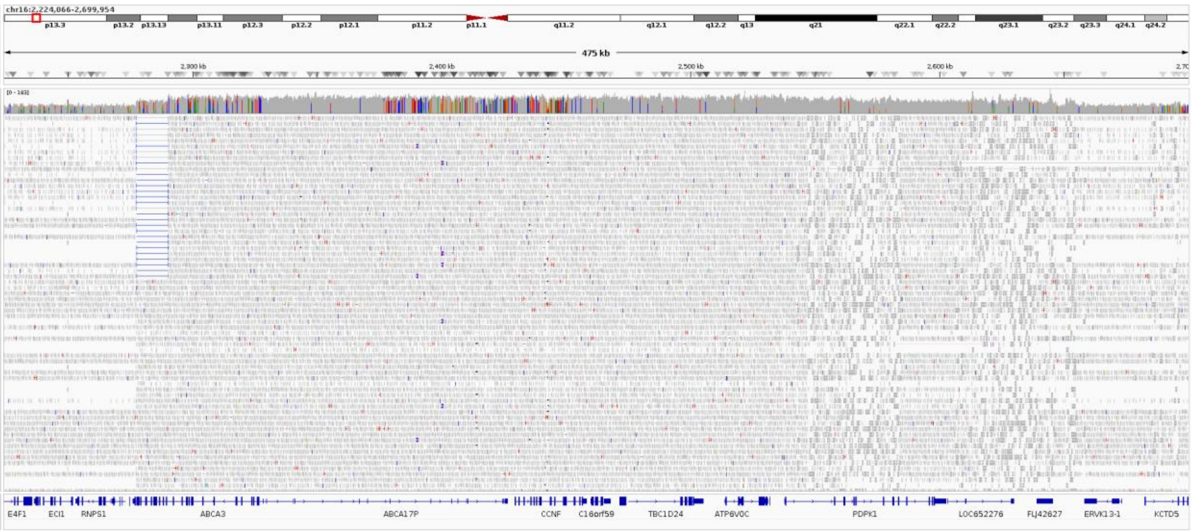

Figure S6: Read alignments from genome sequencing data supporting the SV in Individual 8.

In contrast to the other UK families which were sequenced as part of the 100kGP, this individual was sequenced as part of the NHS GMS. The distal ends of the SV are where split read-pairs both mapping to the negative strand are highlighted in blue. These breakpoints coincide with a stepped increase of read coverage from 2 to 3, then from 3 to 4 copies and lie in *ABCA3*. 150 bp reads are viewed in IGV using “collapsed” and “view as pairs” settings.

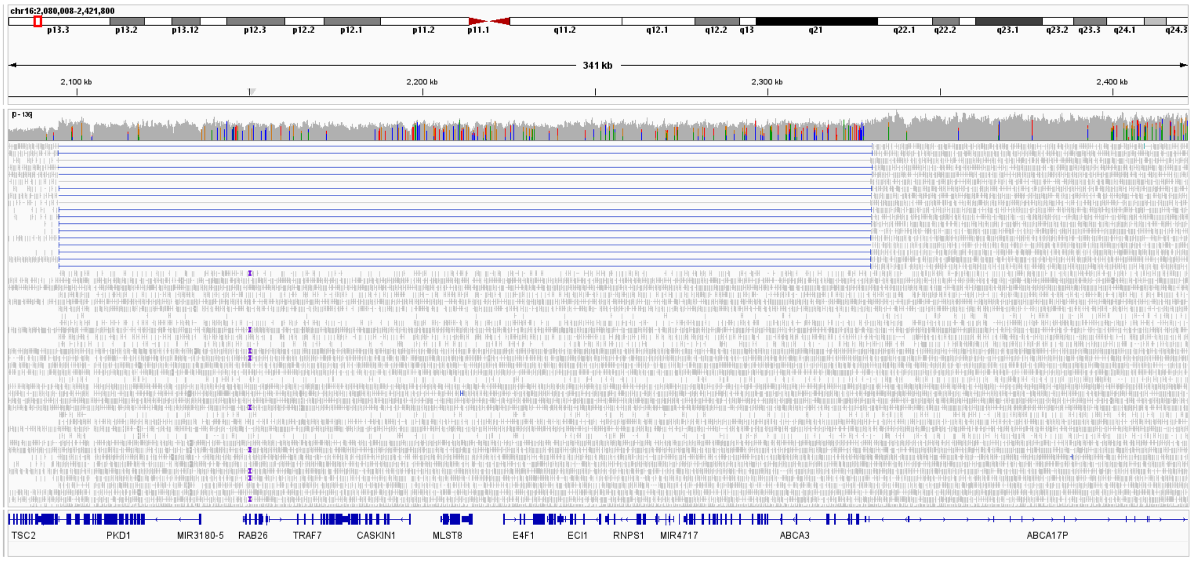

Figure S7: Read alignments from genome sequencing data supporting the SV in the proband (III-3) from Family 1.

The distal end of the SV is shown in IGV, where split read-pairs both mapping to the negative strand are highlighted in blue. These breakpoints coincide with a stepped increase of read coverage from 2 to 3, then from 3 to 4 copies. 150 bp reads are viewed using “collapsed” and “view as pairs” settings. This distal breakpoint was further supported by the presence of a fusion transcript detected between *PKD1* and *ABCA3* (**Figure S16-17**).

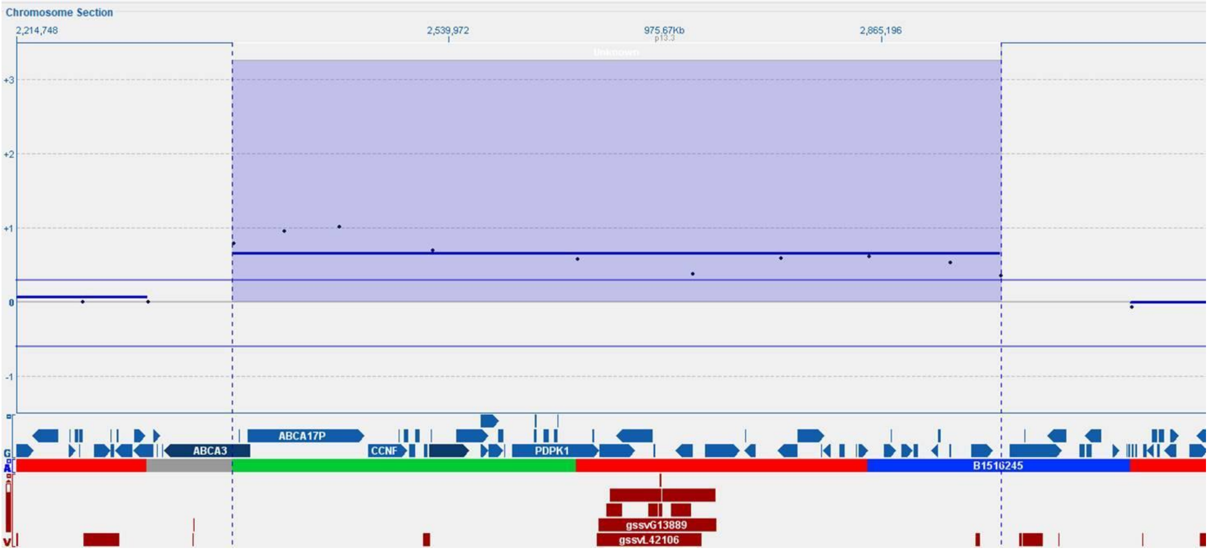

Figure S8: Low resolution array-based comparative genomic hybridization (aCGH) data for Individual 3.

Probe intensity data is plotted against GRCh37/hg19 chromosome position where results are consistent with a copy number increase for this locus on 16p13.3. There are 10 probes inside the SV and at this resolution it is hard to distinguish whether there is a triplicated segment within the duplication. The algorithmic duplication call is shaded in blue and represents the minimal region. Experiments were performed using the 8x60K constitutional v3.0 array. Hybridizations were carried out using pooled, sex matched DNA as a control and data analysis was with CytoSure Interpret v4.9 (Oxford Gene Technology).

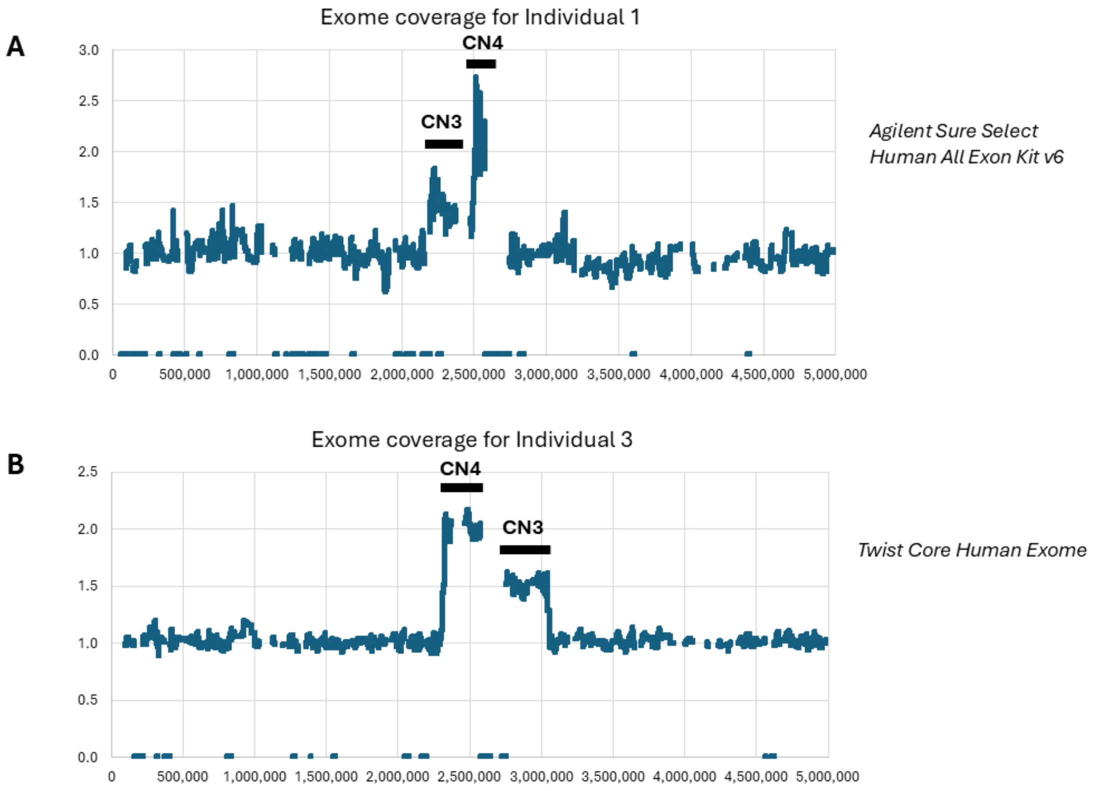

Figure S9: Read coverage analysis of exome data identifies triplicated segments.
Coverage in the proband is normalized to the mean coverage across both unaffected parents who were sequenced using the same methodology. Relative coverage was then plotted for overlapping windows corresponding to 3kb of the target region. Data shown is mapped to GRCh37/hg19. Data for Individuals 1 and 3 is shown in panels A and B, respectively. Although different capture kits were used, in both cases sequencing was performed on the Illumina NextSeq machine.

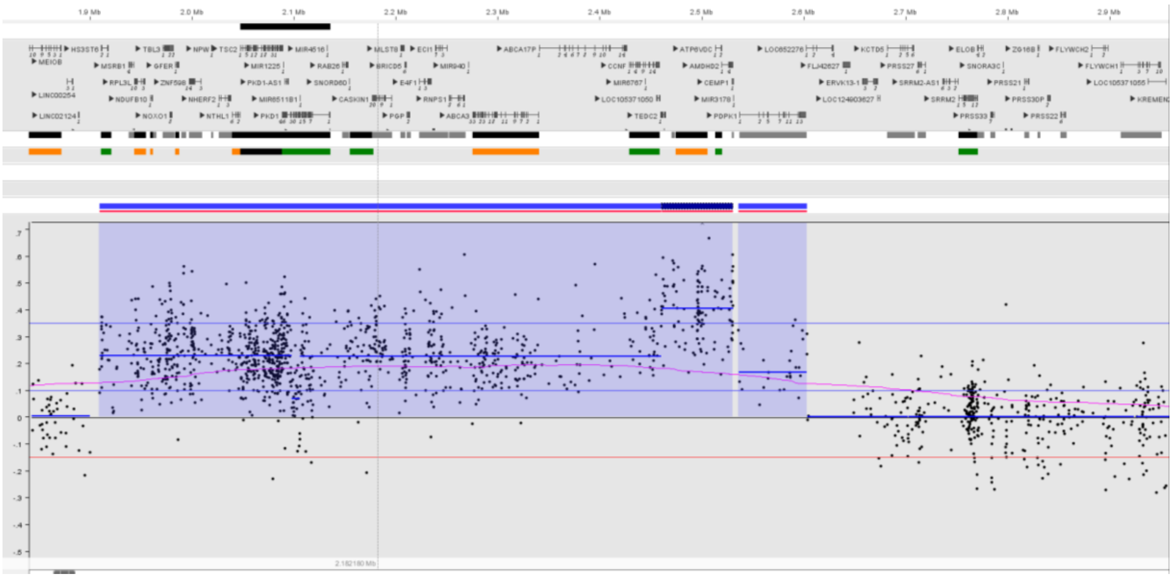

Figure S10: Infinium Global Diversity Array-Cyto data for Individual 2.

The ~1.8M probes on this array results in a much higher resolution than had been obtained with earlier array testing. Probe intensity data is plotted against chromosome position where results are consistent with a small triplication of 16p13.3 embedded within a duplication. Region shown is chr16:1,842,971-2,964,603 (GRCh38). Confident software calls are shown in blue and purple shading and OMIM morbid genes are highlighted above in green/orange. A pink line shows a moving average of the probe intensity data. The genes *ATP6V0C* and *AMDHD2* are localised within the triplicated segment.

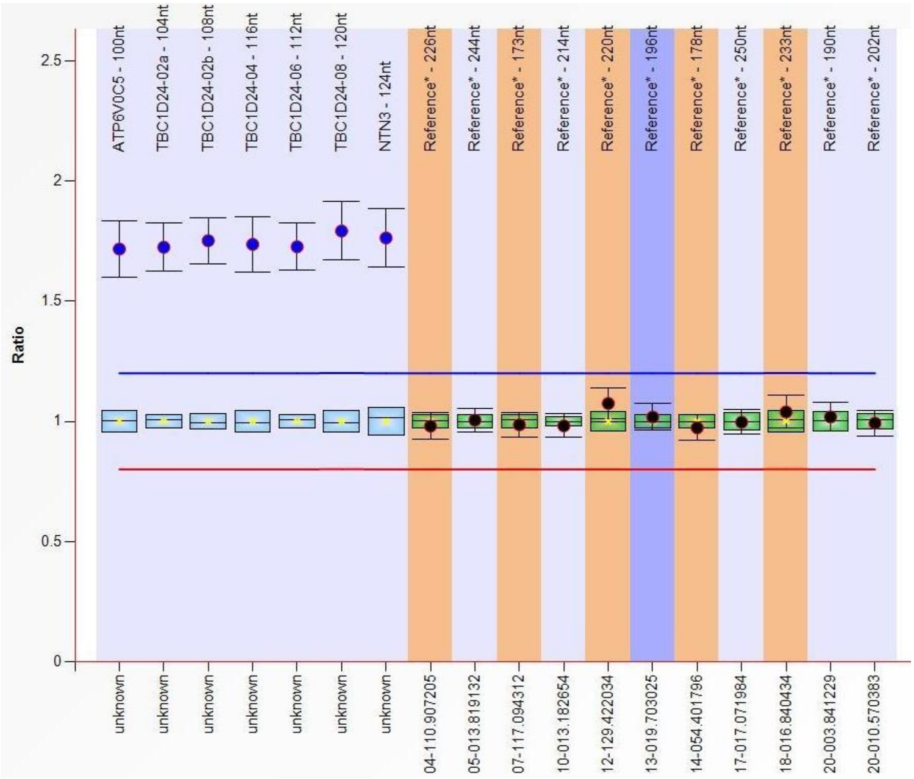

Figure S11: Data from MLPA testing for Individual 7 confirmed the variant to be *de novo*.

MLPA kit P200-B1 (MRC Holland), which contains reference probes and control fragments was customized to include 7 additional probes corresponding to *ATP6V0C*, *TBC1D24* and *NTN3*. Although initially reported as a heterozygous duplication, retrospective analysis shows all probes within the structural variant (SV) have an increased signal ratio of between 1.5 and 2 which equates to an estimated copy number of between 3 and 4. A retrospective comparison of MLPA probe sequences with genome sequencing defined SV coordinates indicated that all 7 probes lay within the triplicated region (https://genome.ucsc.edu/s/AlistairP/16p13.3_F7_MLPA). Previous testing by array-CGH using an Agilent ISCA 60K oligoarray had also failed to distinguish between duplication and triplicated segments.

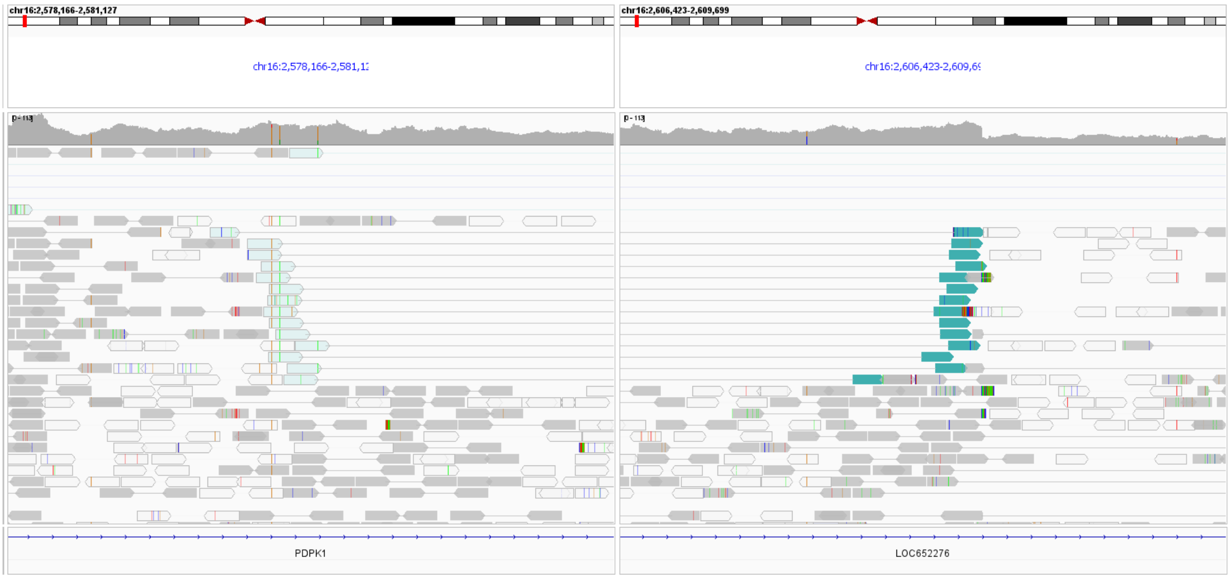

Figure S12: Read alignments from genome sequencing data supporting SV in the proband (III-3) from Family 1.

The proximal ends of the SV are shown in IGV using split view, where split read-pairs both mapping to the positive strand are highlighted in teal. The most proximal breakpoint coincides with a clear decrease in read coverage, corresponding to a drop from 3 to 2 copies. 150 bp reads are viewed using “view as pairs” settings. Due to the presence of the large palindromic repeat, many reads have a low mapping quality and these are shaded in white.

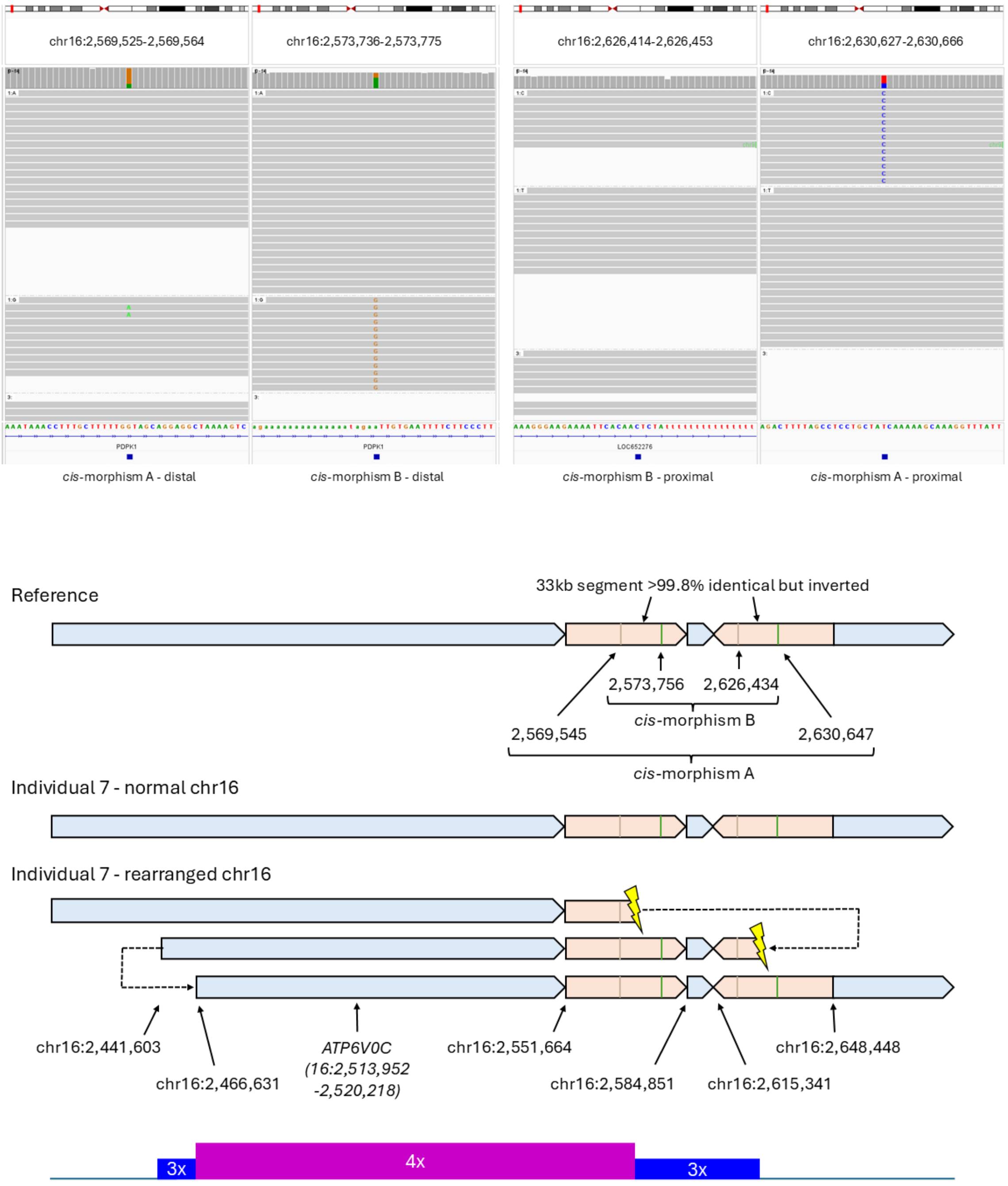

Figure S13: Split-screen IGV screenshot showing HiFi PacBio read alignments at *cis*-morphic sites in Individual 7.

The GRCh38 genomic coordinates of these 4 *cis*-morphic sites are: 16:2569545, 16:2573756, 16:2626434 and 16:2630647 and all are within the palindromic repeat, close to the suspected proximal end of the SV. Reads are grouped by base at position 16:2573756 and 16:2630647. Several reads span between *cis*-morphisms A and B. Several reads harbor one but not the other *cis*-morphism and this suggests that the proximal SV breakpoints may lie in the 4.2 kb interval between these positions. Positions of *cis*-morphic sites are available in an interactive UCSC session http://genome.ucsc.edu/s/AlistairP/16q13.3_cismorphismsV2. The schematic diagram includes a

subway plot that explains how the proximal breakpoint of the rearrangement could explain both the anomalous PacBio reads and the changes in copy number state.

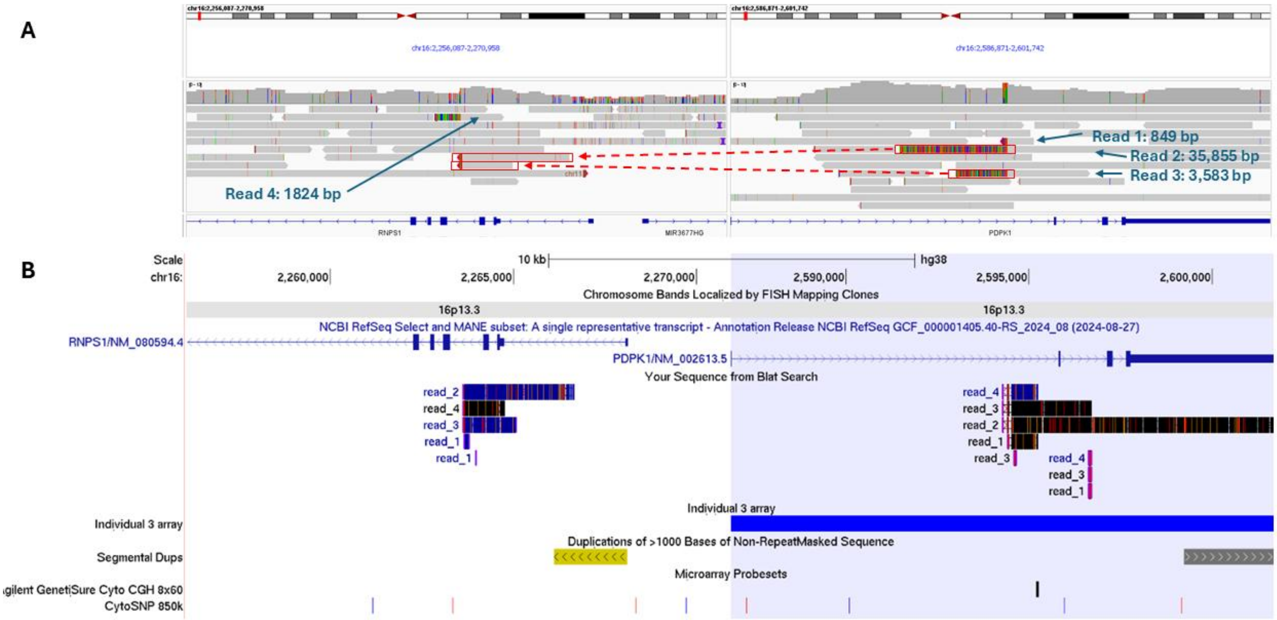

Figure S14: Low coverage Oxford Nanopore sequencing resolves breakpoint in Individual 3.

A) Read alignments shown in IGV. Regions shown are chr16:2,256,087-2,270,958 and chr16:2,586,871-2,601,742 (GRCh38). B) UCSC multi-region browser graphic showing BLAT search results for the 4 reads (as labelled in panel A) which span the distal breakpoint. This session is available interactively at https://genome.ucsc.edu/s/AlistairP/16p3.3_I3_nanopore. The duplication start point was underestimated by around 50 kb from the array testing. The structure for this SV is most similar to that seen in Individual 5 (**Figure S4**). The black and dark blue shading for the BLAT sequence track denotes whether the sequence maps to the positive or negative strand and so the results indicate some form of inversion.

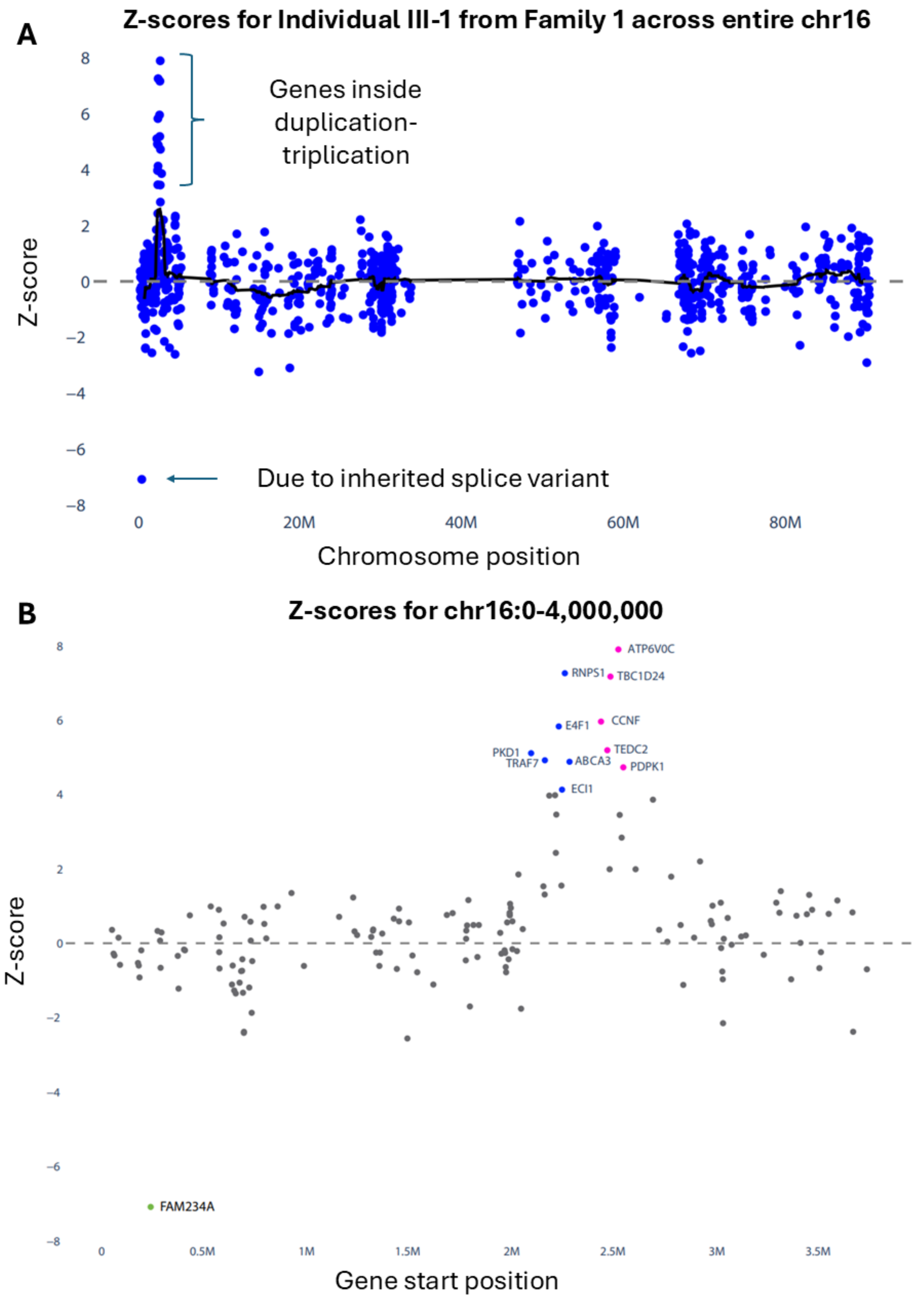

Figure S15: RNA-seq expression Z-scores for Individual III-3 from Family 1 plotted against chromosome position.

A) Whole chromosome view shows clear outlier at the end of the short arm (16p13.3) which coincides with the position of the duplication-triplication. Black line represents a moving-window average where the window size is dynamically set to 5% of the length of the chromosome. B) As

above, but zoomed in to chr16:0-4,000,000 and with gene symbols labelled. Although several genes within the 16p13.3 SV show higher than expected expression, the most significant result was for *ATP6V0C*, with a Z-score of 7.9. Reduced expression of *FAM234A* (Z-score of -7.1) was likely due to a heterozygous NM_032039.4:c.-140+2T>G variant. Significantly upregulated genes that lie within the 16p13.3 duplication or triplication segments are highlighted in blue and purple, respectively. Chromosome 16 positions are based on the GRCh38 reference.

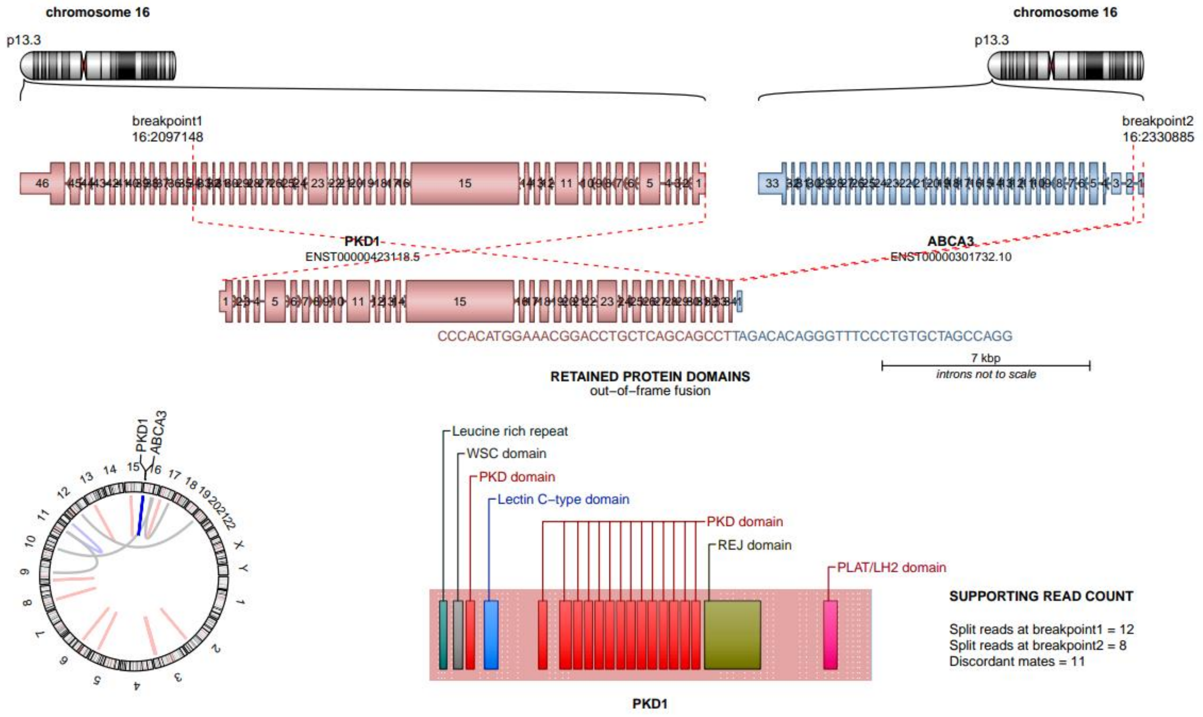

Figure S16: RNA-seq data identified **a** *PKD1-ABCA3* fusion transcript in Individual III-3 from Family 1.

The *PKD1-ABCA3* fusion transcript is highlighted in the circus plot in blue and the types and numbers of supporting reads are summarized. The fusion was ranked as the top hit by Arriba (https://github.com/oicr-gsi/arriba). The fusion junction is between the end of *PKD1* exon 34 (ENST00000423118.5) in the coding direction and the first intron of *ABCA3* (ENST00000301732.10) in the non-coding direction. The sequences corresponding to the breakpoint are shown in https://genome.ucsc.edu/s/AlistairP/16p13.3_F1_fusion in the BLAT search track. Exons 1-34 of *PKD1* are in an inverted orientation.

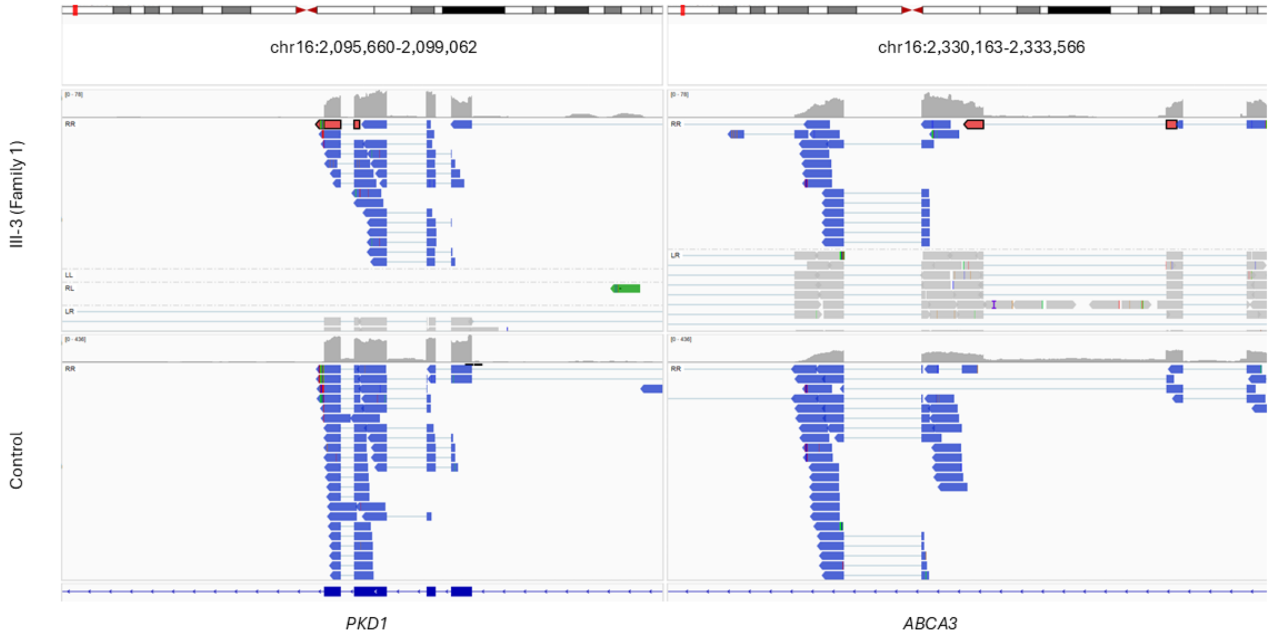

Figure S17: RNA-seq data for Individual III-3 of Family 1 supports presence of a fusion transcript.

Split-screen IGV screenshot showing RNA-seq read-alignments supporting the presence of a *PKD1-ABCA3* fusion transcript. The two reads highlighted in red are pairs that span the junction of the gene-fusion and correspond closely to the distal SV breakpoint. Both map to the negative strand of GRCh38. Data is for Individual III-3 from Family 1 (above) and a control RNA-seq sample (below).

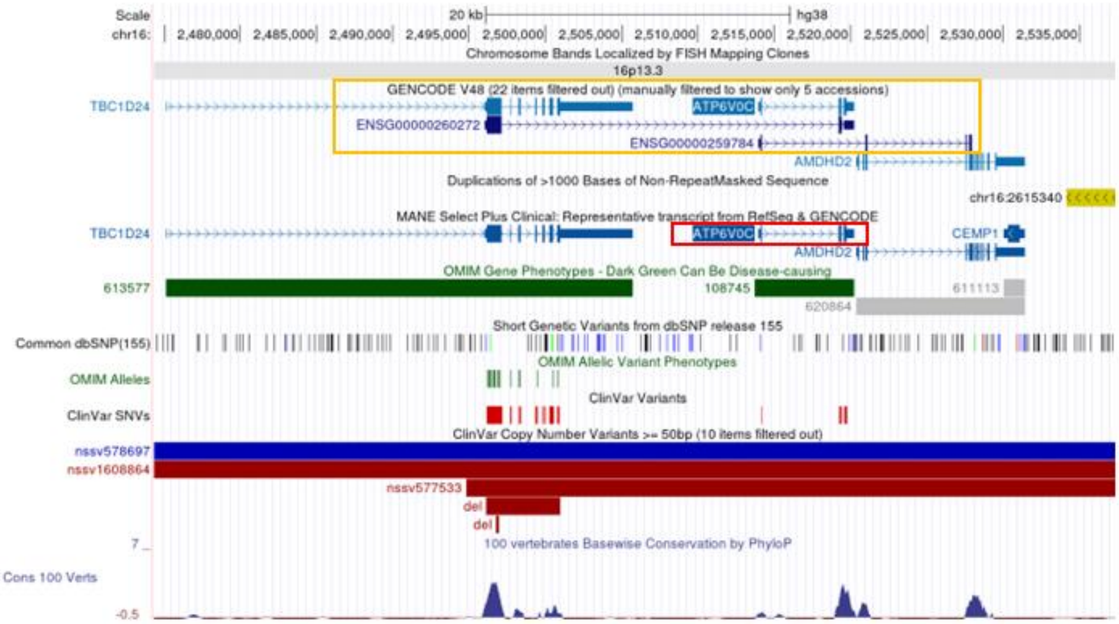
Scale
20 kb
hg38
chr16:
2,480,000
2,485,000
2,490,000
2,495,000
2,500,000
2,505,000
2,510,000
2,515,000
2,520,000
2,525,000
2,530,000
2,535,000
Chromosome Bands Localized by FISH Mapping Clones
16p13.3
GENCODE V48 (22 items filtered out) (manually filtered to show only 5 accessions)
TBC1D24
ATP6V0C
ENSG00000260272
ENSG00000259784
AMDHD2
Duplications of >1000 Bases of Non-RepeatMasked Sequence
chr16:2615340
MANE Select Plus Clinical: Representative transcript from RefSeq & GENCODE
TBC1D24
ATP6V0C
CEMP1
AMDHD2
OMIM Gene Phenotypes - Dark Green Can Be Disease-causing
613577
108745
611113
620864
Short Genetic Variants from dbSNP release 155
Common dbSNP(155)
OMIM Allelic Variant Phenotypes
OMIM Alleles
ClinVar Variants
ClinVar SNVs
ClinVar Copy Number Variants >= 50bp (10 items filtered out)
nssv578697
nssv1608864
nssv577533
del
del
7
100 vertebrates Basewise Conservation by PhyloP
Cons 100 Verts
-0.5

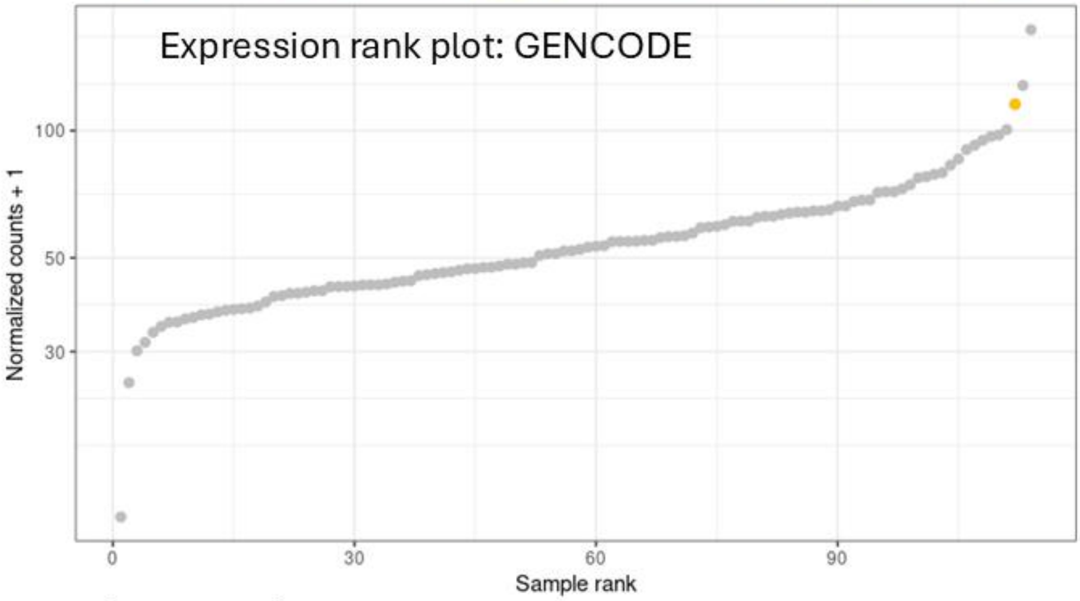
Expression rank plot: GENCODE
Normalized counts + 1
100
50
30
0
30
60
90
Sample rank

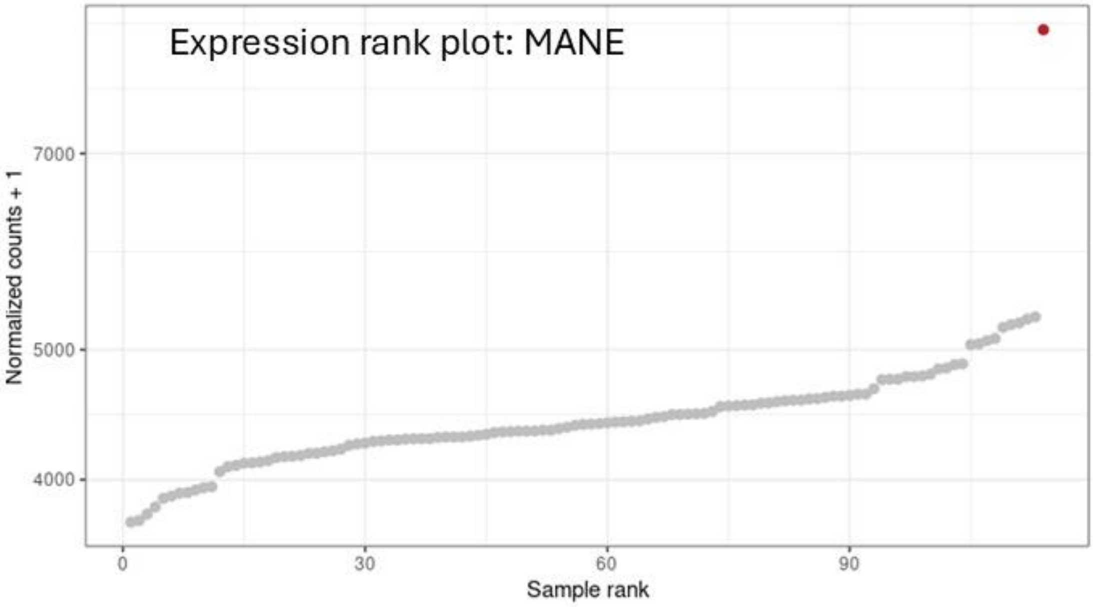
Expression rank plot: MANE
Normalized counts + 1
7000
5000
4000
0
30
60
90
Sample rank

Figure S18: RNA-seq data for Individual 2 highlights the importance of using appropriate transcript annotations.

UCSC genome browser session showing Genecode V48 (orange box) and MANE Select gene annotations (red box) for *ATP6V0C* (ENSG00000185883.12), which are identical. However, for the Genecode annotations, there are two additional fusion transcripts, for *TBCD1D24-ATP6V0C* (ENSG00000260272.1) and *ATP6V0C-AMDHD2* (ENSG00000259784.1). An interactive view is available at https://genome.ucsc.edu/s/AlistairP/ATP6V0C_RNAseq_artifact. Lower panels show RNA-seq results from lymphoblast-derived RNA for Individual 2 which was part of a cohort (N=114) analyzed with OUTRIDER. With the Genecode annotations, normalized read counts for *ATP6V0C* are typically low (30-100) and Individual 2 was ranked 3rd in terms of relative expression (Z-score = 2.34). Close scrutiny suggested that these results were being confounded by Gencode annotations for fusion genes that overlap the MANE *ATP6V0C* transcript. After repeating the OUTRIDER analysis using just the "MANE select" annotations, normalized *ATP6V0C* read counts across the cohort were much higher (4000-5000) and Individual 2 was a clear outlier, ranked as having the highest relative expression for this gene (FC=1.95, Z-score = 6.86).

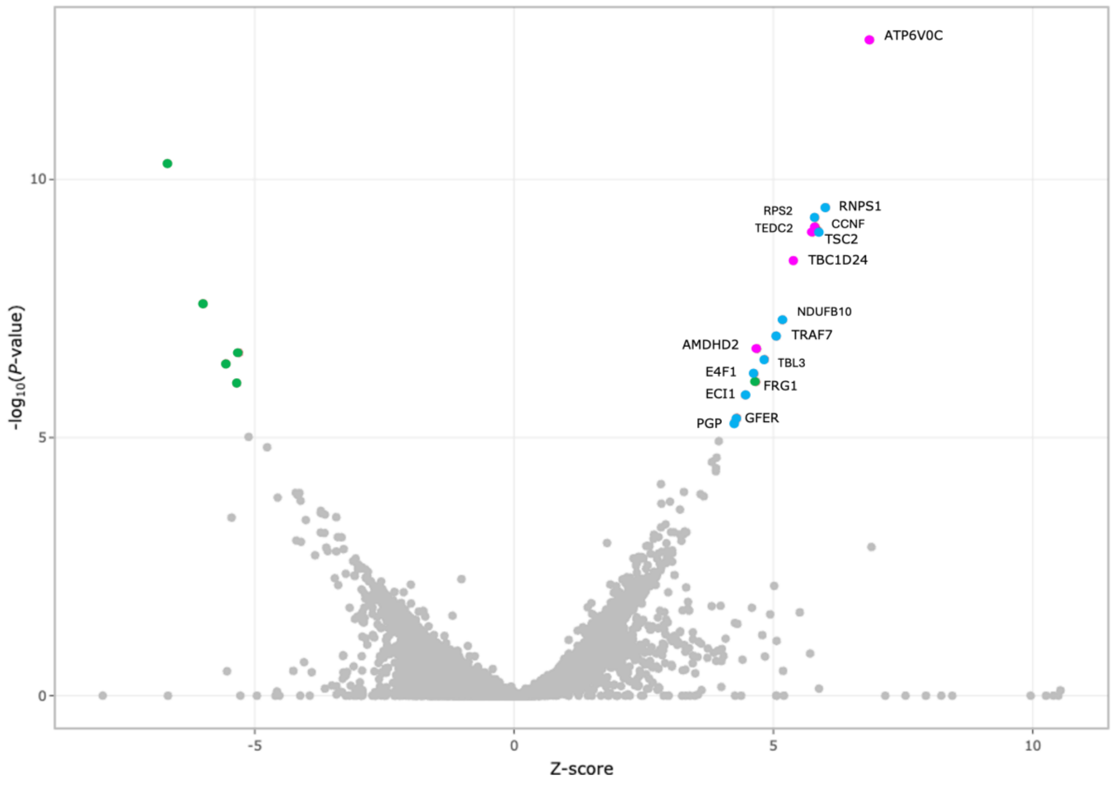

Figure S19: Volcano plot of RNA-seq data for Individual 2.

RNA-seq data for lymphoblast-derived material from an individual with rare rearrangement on 16p13.3 confirms aberrant gene expression. Genome-wide volcano plot showing genes that had significantly aberrant expression (*P*-value < 1×10^{-5}) in Individual 2. Significantly upregulated genes that lie inside the 16p13.3 duplication/triplication are highlighted in blue/purple, respectively. Genes outside the SV are labelled in green. Similar to the results for the proband in Family 1 (**Figure 3A)**, the most significant result was *ATP6V0C,* which lies inside the triplicated segment and has a Z-

score of 6.86. Of the 16 most significantly upregulated genes, 15 lay within the 16p13.3 rearrangement (**Table S3**).

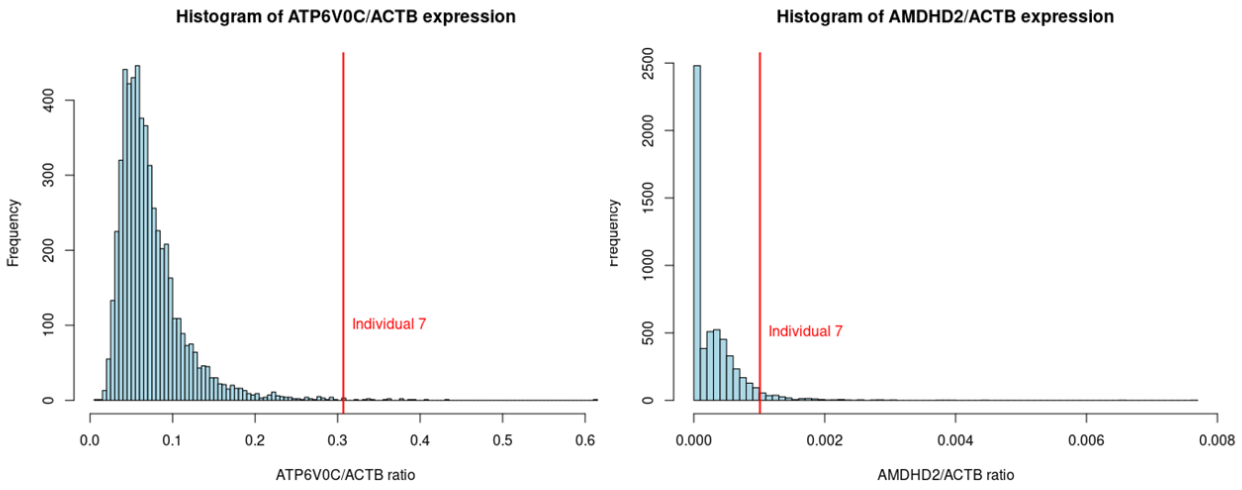

Figure S20: Relative RNA expression of *ATP6V0C* and *AMDHD2* is elevated in Individual 7.

When normalized to *ACTB*, a second housekeeping gene, expression of *ATP6V0C* was 4.16x above the mean and *AMDHD2* was 3.48x above the mean. Results for all 5,546 datasets are shown. Data normalized to *GAPDH* are shown in **Figure 3C-D**.

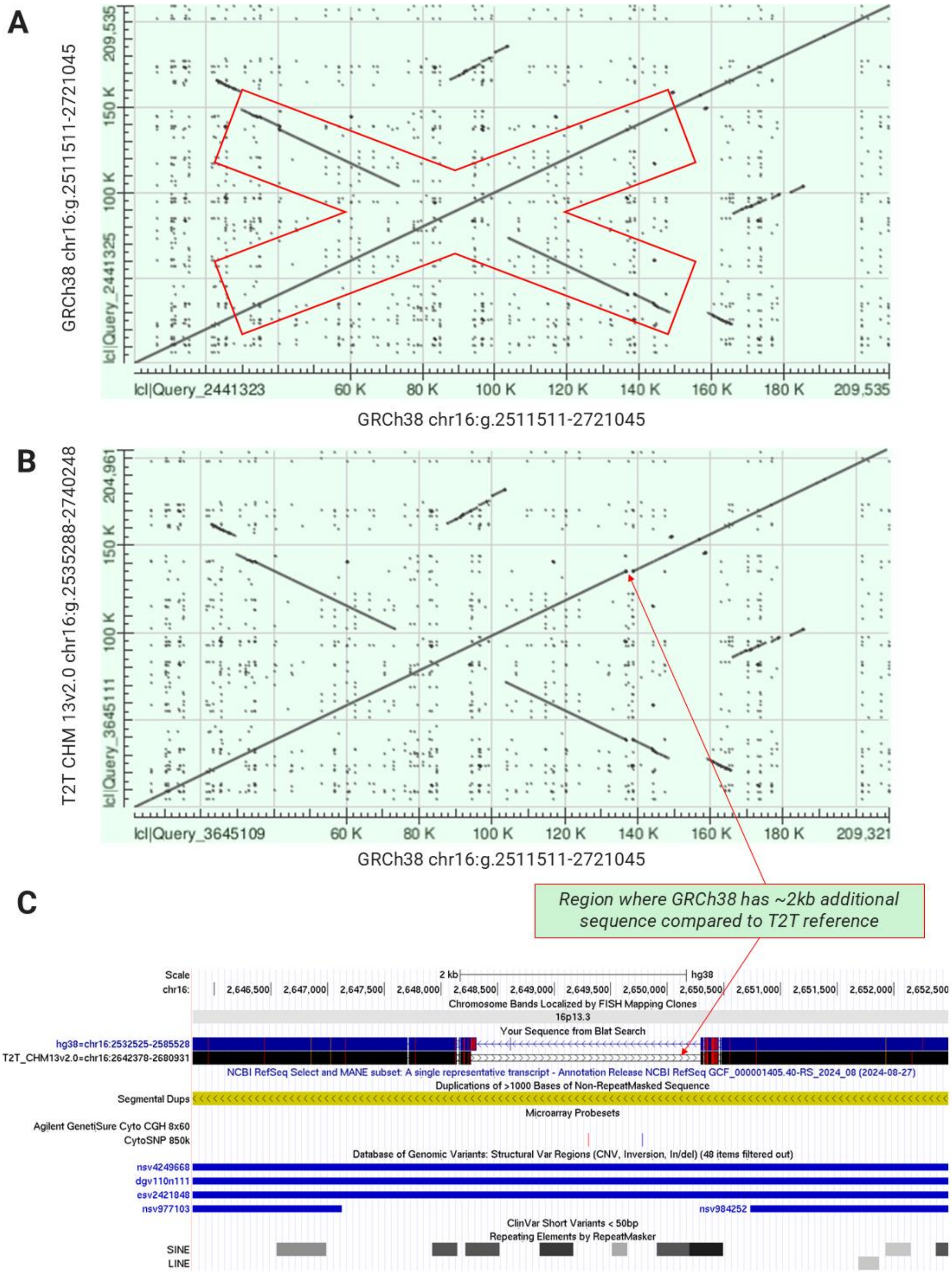

Figure S21: Comparison of the palindromic repeat sequence on 16p13.3 within and between different reference builds.

A) Dotplot creased using 209.5 kb of sequence from 16p13.3 showing the presence of a large palindrome-like repeat (red cross) present in GRCh38. B) A similar comparison of the same region between GRCh38 and the latest available reference from the Telomere-to-Telomere consortium

(CHM 13v2.0) highlights a site that differs between the two builds. Dotplots were created using the NCBI BLAST tool and default settings, with the “highly similar sequences (megablast)” option. C) Genome browser graphic for the discordant region showing that GRCh38 contains an additional ~2kb of sequence compared with both the CHD 13v2.0 genome and with the paralogous repeat nearby in GRCh38. An interactive UCSC session for this region (chr16:2,645,808-2,652,528) is available at https://genome.ucsc.edu/s/AlistairP/16p13.3_hg38vsT2T. The black and dark blue shading for the BLAT sequence track denotes whether the sequence maps to the positive or negative strand. In addition to the change highlighted above, we note that GRCh38 also contains an alternate contig (KQ090026v1) that corresponds to alternative haplotype for a nearby region.

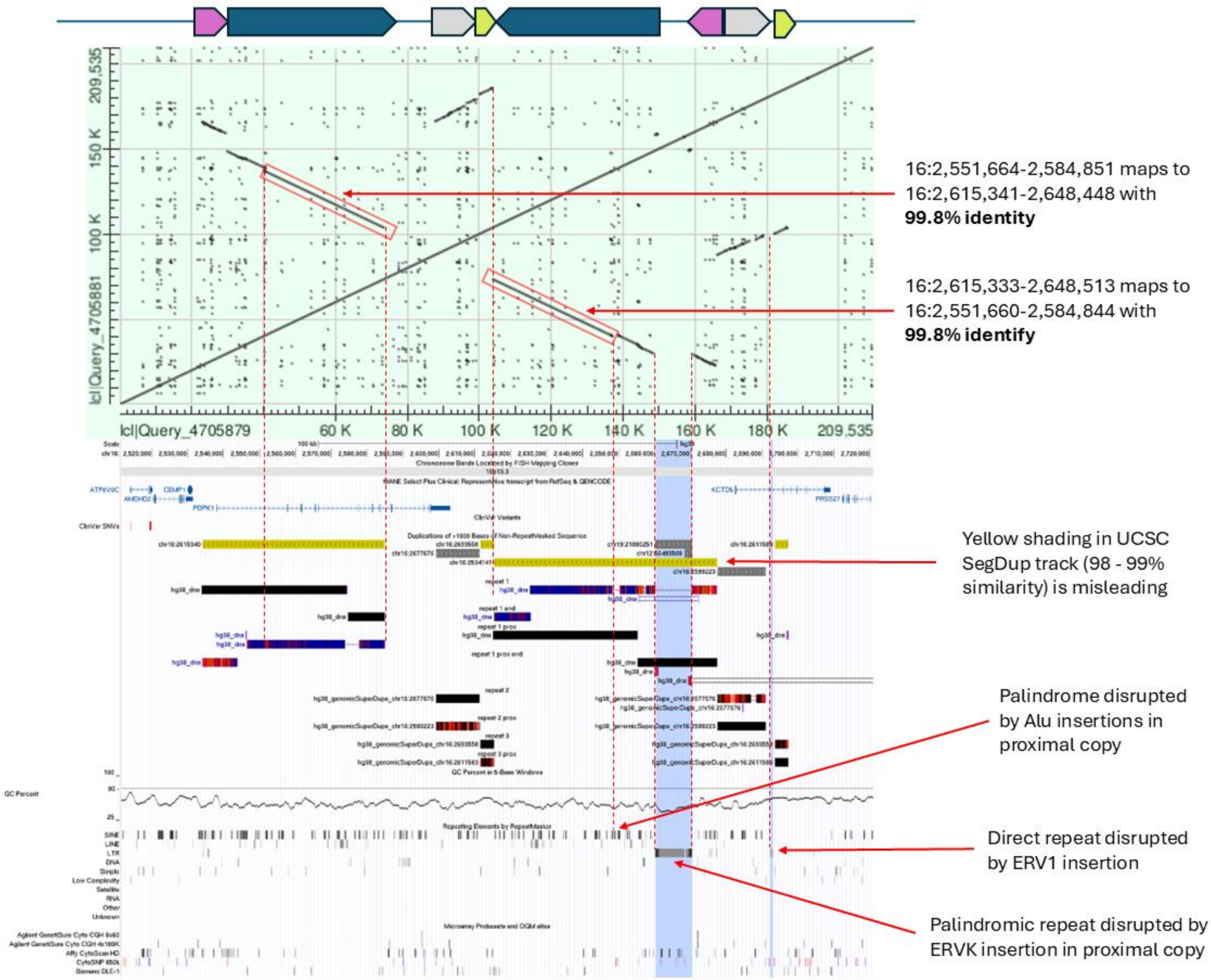

Figure S22: Further review of GRCh38 sequence for the palindromic repeat region (chr16:2,511,511-2,721,045).

The dotplot generated by the NCBI the Blat2seq tool highlights that within the annotated segmental duplication there is a segment of 33kb with a particularly high level of sequence identity at 99.8%. This repeat is present in a plus/minus orientation and thus represents a palindrome-like structure. Long terminal repeat (LTR) element insertions which disrupt the proximal repeat units are highlighted in light blue. Dotted red lines connect the ends of the repeat segments identified in the dotplot to the equivalent positions on the BLAT sequence tracks on the UCSC browser graphic to help uncover the genomic features that interrupt the repeat units. BLAT tracks can only show 40kb

of sequence so the main repeat is split into two BLAT tracks. The schematic diagram above the dotplot represents a simplified representation of the repeat structure. GC content across the region is unremarkable. An interactive version of UCSC graphic is available at https://genome.ucsc.edu/s/AlistairP/16p13_palindrome_v3.

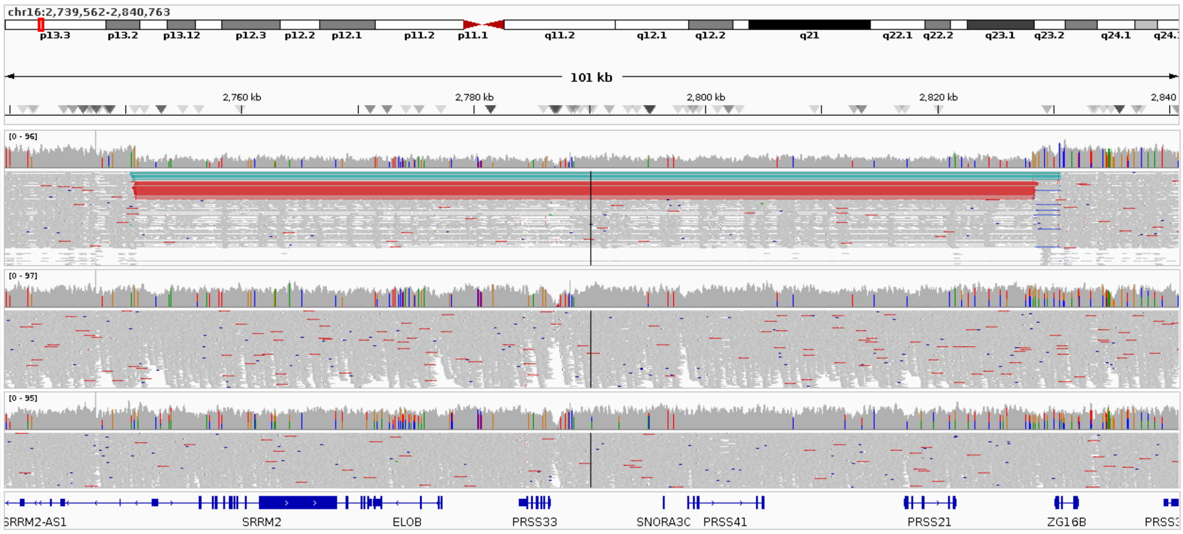

Figure S23: Read alignments supporting a complex deletion-inversion variant on 16p13.3 in an individual from the NHS Genomic Medicine Service.

The SV is shown in IGV, where split read-pairs both mapping to the positive strand are highlighted in teal and pairs mapping to the negative strand in blue. Read-pairs highlighted in red are those that span right across the 166 bp inserted/inverted segment. Although reported clinically as chr16:g.2750923_2828204del (GRCh38), this ignores the presence of the internal inversion. The 150 bp reads are viewed using the “squished” and “view as pairs” settings. Parental data, shown in the bottom two tracks, indicate this SV to have arisen *de novo*. As *SRRM2* lies in the middle of this heterozygous deletion, this finding is consistent with a diagnosis of autosomal dominant intellectual developmental disorder, type 72 (MIM: 620439). The individual’s clinical features, focal-onset epilepsy, learning disability requiring specialist schooling, ADHD, and behavioral difficulties, are concordant with the phenotype previously described for this condition. In addition, primary hypothyroidism with constitutional short stature (0.4th centile) was noted, a feature not previously associated with *SRRM2*-related neurodevelopmental disorder, and potentially representing phenotypic expansion. This observation supports our prior report of complex SVs at this locus.[4]

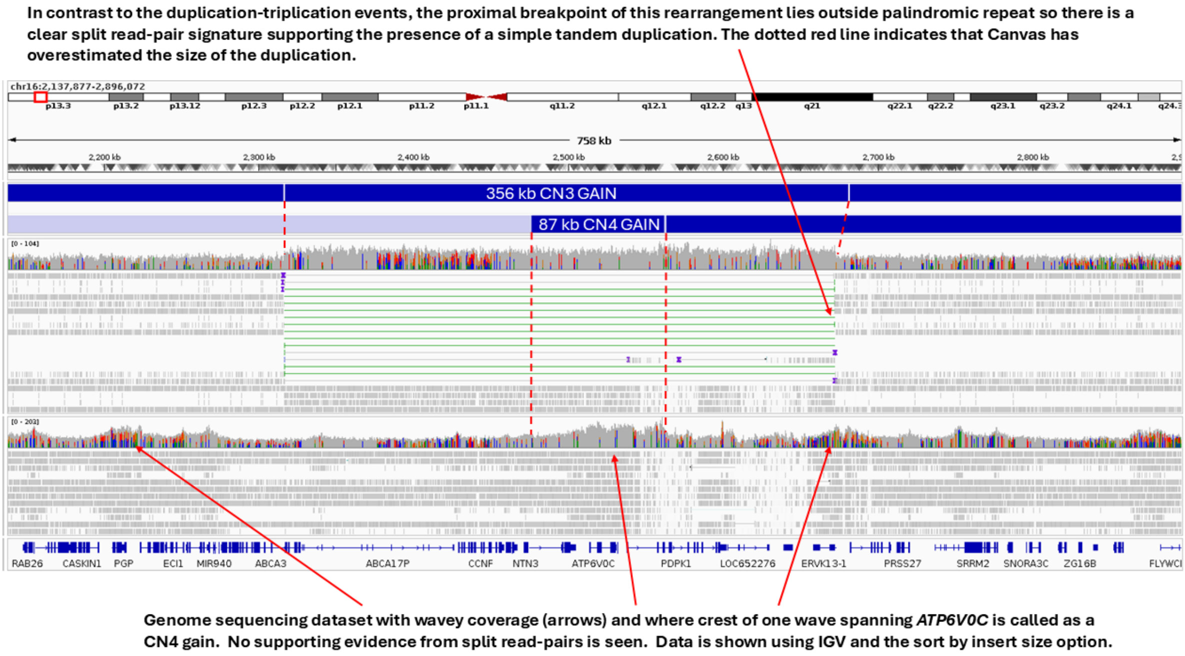

Figure S24: Read alignments showing a tandem-duplication and a false positive triplication call from the 100k Genomes Project.

IGV screenshot showing read alignments confirming the presence of a 356 kb tandem duplication (middle track), where 150 bp split read-pairs coincide with an increase in read depth. The proximal end of the duplication was overestimated by Canvas. In the bottom track, a wavey pattern is seen (see highlighted red arrows) that suggests poor sample quality. There are also no discrete steps in read coverage and no split read-pairs to support the 87 kb SV call.

	Individual 1	Individual 2	Individual 3	Individual 4	Individual 5	Individual 6	Individual 7	Individual 8	Family 1
**CNV detection by**	Array + ES	Array + GS	Array, ES, LR	Array + GS	GS	GS	GS + LR	GS	Array + GS
**Duplication coordinates** (chr16:GRCh38)<br><br>**Duplication size**	2,137,455-2,585,920†<br><br>448 kb	1,901,911-2,606,201‡<br><br>704 kb	2,263,630-2,903,967†§<br><br>640 kb	1,739,210-2,657,000<br><br>918 kb	2,337,167-2,566,956<br><br>230 kb	2,480,209-2,637,000<br><br>157 kb	2,441,603-2,628,540[1]<br><br>187 kb	2,277,344-2,655,000<br><br>378 kb	2,094,750-2,608,383<br><br>514 kb
**Triplication coordinates** (chr16:GRCh38)<br><br>**Triplication size**	2,499,243-2,529,370<br><br>30 kb	2,382,641-2,561,336<br><br>179 kb	2,277,490-2,594,500<br><br>317 kb	1,758,994-2,570,189<br><br>811 kb	2,503,226-2,566,956<br><br>64 kb	2,481,567-2,566,956<br><br>85 kb	2,466,631-2,571,650<br><br>105 kb	2,290,102-2,586,432<br><br>296 kb	2,330,064-2,579,700<br><br>250 kb
**Inheritance**	*de novo*	*de novo*	*de novo*	*de novo*	Father mosaic	NK	*de novo*	NK	Maternal/NK[2]

Table S1: Details of overlapping duplication/triplications involving 16p13.3 in the 8 individuals and one multiplex family in this study.

ES, Exome sequencing; GS, Genome Sequencing; LR, Long-read genome sequencing; NK, not known. Genomic coordinates are based on NC_000016.10 (GRCh38). There remains some uncertainty about the precise coordinates, particularly at the proximal end and for Individuals 1 and 3 where only low resolution (†) array and exome data was available. ‡, For Individual 2, although high resolution array data was available, coordinates are taken from genome sequencing data. §, Individual 3 where low resolution array and exome was initially performed but long-read sequencing was also undertaken. Although array/exome coordinates are reported, Oxford Nanopore data suggests a distal breakpoint at 16:2,263,633:2,594,508 (**Figure S14**). [1]For Individual 7, the proximal breakpoints are taken as the midpoint between the two *cis*-morphisms. [2]Presumed *de novo*; not paternally inherited, no DNA available from deceased mother.

Table S2: Information for genes on 16p13.3 within or near the shared region of triplication.

Missense constraint and pLI scores are from gnomAD 4.1.0. FC, fold-change. aa, amino-acids. AR, autosomal recessive. AD, autosomal dominant. †Also linked to Deafness, autosomal dominant 65 (MIM: 616044) and Deafness, autosomal recessive 86 (MIM: 614617). ‡, partially triplicated in 8/9 but significant uncertainty. Mouse data was from www.informatics.jax.org and www.mousephenotype.org.

Gene symbol	*TBC1D24*	*ATP6V0C*	*AMDHD2*	*CEMP1*	*PDPK1*
Full gene name	TBC1 domain family member 24	ATPase H+ transporting V0 subunit c	amidohydrolase domain containing 2	cementum protein 1	3-phosphoinositide dependent protein kinase 1
HGNC ID	29203	855	24262	32553	8816
MANE ID (Refseq)	NM_001199107.2	NM_001694.4	NM_001330449.2	NM_001048212.3	NM_002613.5
Exon number	8	3	11	1	14
Protein size (aa)	559	155	409	247	556
Genomic position (GRCh38)	16:2,475,051-2,509,560	16:2,513,952-2,520,218	16:2,520,357-2,531,422	16:2,530,035-2,531,417	16:2,537,979-2,603,188
MIM gene number	613577	108745	620864	611113	605213
MIM condition	Familial infantile myoclonic epilepsy (605021, AR)[5], developmental and epileptic encephalopathy 16 (615338, AR)[6], DOORS syndrome (220500, AR)[7], Epilepsy, rolandic, with paroxysmal exercise-induce dystonia and writer's cramp (608105, AR)[8]†	Epilepsy, early-onset, 3, with or without developmental delay (620465, AD)[9]	NA	NA	NA
PanelApp panels (assessed 29th September 2025)	Fetal anomalies (v6.86), DDG2P (v6.5), Monogenic hearing loss (v5.27), Early onset or syndromic epilepsy (v8.33), Intellectual disability (v9.99)	Severe microcephaly (v8.12), DDG2P (v6.5), Early onset or syndromic epilepsy (v8.33), Intellectual disability (v9.99)	NA	NA	NA
GTEx expression in cerebellum (median TPM)	28.27	643.3	24.48	NA	33.73

Number of families full gene is triplicated in current study	6/9	9/9	8/9	8/9	0/9‡
RNAseq data for Family 1 (FC and rank), Table S3	1.95 (3)	1.83 (1)	-	-	1.47 (15)
RNAseq data for Individual 2 (FC and rank), Table S4	1.59 (8)	1.95 (1)	1.83 (12)	-	-
Triplosensitivity score[10] (pTriplo, from Decipher)	0.23	0.48	0.56	0.09	0.99
pLI score	0	0.74	0	NA	1.00
Missense constraint (Z-score)	1.10	2.91	0.39	-1.11	2.13
Mouse data - from Mouse Genome Informatics (MGI) and International Mouse Phenotyping Consortium (IMPC)	Heterozygous knockout mice show normal growth, neurodevelopment, and hearing with normal macroscopic brain appearance. Their hippocampal and cortical neurons show abnormal growth.[11]<br><br>Homozygous knockout animals show perinatal or postnatal lethality (MGI).<br><br>Homozygous, but not heterozygous conditional knockout animals exhibited spontaneous tonic–clonic seizure and normal hearing with normal macroscopic brain appearance.[12,13]	Homozygous knockout animals show abnormal embryogenesis and embryonic lethality before implantation (MGI).	Heterozygous knockout mice show altered behavior and impaired glucose tolerance. Homozygous knockout animals show preweaning lethality (MGI / IMPC).	Nil	Mice with conditional knockout exhibit decreased cerebellar size and ataxia-like behavior, suggesting that pdpk1 may be critical for motor balance and coordination.[14]
Summary from RefSeq/GeneCards (www.genecards.org)	This gene encodes a protein with a conserved domain, referred to as the	This gene encodes a component of vacuolar ATPase (V-ATPase), a	Enables N-acetylglucosamine-6-phosphate deacetylase	Enables hydroxyapatite binding activity. Involved in several processes,	Enables 3-phosphoinositide-dependent protein kinase

	TBC domain, characteristic of proteins which interact with GTPases. TBC domain proteins may serve as GTPase-activating proteins for a particular group of GTPases, the Rab (Ras-related proteins in brain) small GTPases which are involved in the regulation of membrane trafficking. Mutations in this gene are associated with familial infantile myoclonic epilepsy. Alternative splicing results in multiple transcript variants. [provided by RefSeq, Feb 2011].	multisubunit enzyme that mediates acidification of eukaryotic intracellular organelles. V-ATPase dependent organelle acidification is necessary for such intracellular processes as protein sorting, zymogen activation, receptor-mediated endocytosis, and synaptic vesicle proton gradient generation. V-ATPase is composed of a cytosolic V1 domain and a transmembrane V0 domain. The V1 domain consists of three A and three B subunits, two G subunits plus the C, D, E, F, and H subunits. The V1 domain contains the ATP catalytic site. The V0 domain consists of five different subunits: a, c, c', c', and d. This gene encodes the V0 subunit c. Alternative splicing results in transcript variants. Pseudogenes have been identified on chromosomes 6 and 17. [provided by RefSeq, Nov 2010].	activity. Involved in negative regulation of UDP-N-acetylglucosamine biosynthetic process. Located in nucleus. [provided by Alliance of Genome Resources, Jun 2025]	including biomineral tissue development; cell population proliferation; and odontogenesis. Located in cytoplasm and nucleoplasm. [provided by Alliance of Genome Resources, Jun 2025]	activity; phospholipase activator activity; and phospholipase binding activity. Involved in several processes, including cell surface receptor signaling pathway; intracellular signaling cassette; and regulation of signal transduction. Acts upstream of or within intracellular signal transduction. Located in cell projection; cytosol; and plasma membrane. Implicated in prostate cancer. Biomarker of lung non-small cell carcinoma. [provided by Alliance of Genome Resources, Jun 2025]

Gene	GRCh38 position	Transcript	*P*-value	*P*-adjust	Z-score	FC	DUP/TRIP
ATP6V0C	chr16:2513951-2520218	NM_001694.4	8.67E-19	1.42E-13	7.90	1.827663	TRIP
RNPS1	chr16:2253119-2268126	NM_080594.4	3.74E-14	2.03E-09	7.26	1.453973	DUP
TBC1D24	chr16:2475126-2505730	NM_001199107.2	1.29E-14	1.06E-09	7.17	1.945310	TRIP
FAM234A	chr16:234820-266096	NM_032039.4	1.65E-11	4.49E-07	-7.09	0.558644	-
SNRNP48	chr6:7590197-7611967	NM_152551.4	6.91E-13	2.82E-08	6.64	1.958841	-
CCNF	chr16:2429446-2458854	NM_001761.3	6.70E-11	1.57E-06	5.96	1.802501	TRIP
E4F1	chr16:2223590-2235742	NM_004424.5	4.64E-10	7.58E-06	5.83	1.424050	DUP
TEDC2	chr16:2460108-2464963	NM_025108.3	1.89E-10	3.85E-06	5.19	2.042024	TRIP
ZNF709	chr19:12461183-12484816	NM_152601.4	3.71E-10	6.74E-06	5.14	2.770219	-
MZT2A	chr2:131483959-131492397	NM_001085365.2	9.93E-09	0.000148	5.12	2.027919	-
PKD1	chr16:2088707-2135898	NM_001009944.3	4.34E-08	0.000592	5.11	1.613284	DUP (exons 1-34)
RNASEH2A	chr19:12806583-12813640	NM_006397.3	4.25E-07	0.004958	-5.05	0.683020	-
TRAF7	chr16:2155781-2178129	NM_032271.3	2.51E-07	0.003160	4.92	1.283426	DUP
ABCA3	chr16:2275880-2340728	NM_001089.3	1.03E-11	3.38E-07	4.88	8.633826	TRIP (exon 1) and DUP (exons 2-33)
PDPK1	chr16:2538020-2603188	NM_002613.5	5.70E-07	0.006213	4.73	1.474269	TRIP (exons 1-7) and DUP (exons 8-14)
PTPRG	chr3:61561570-62297609	NM_002841.4	1.25E-05	0.106629	-4.71	0.558644	-
NAGLU	chr17:42536240-42544449	NM_000263.4	1.30E-05	0.106629	-4.60	0.586417	-
MED29	chr19:39391377-39400641	NM_017592.4	1.62E-06	0.016512	4.38	2.158456	-
PDIA6	chr2:10783390-10812785	NM_005742.4	6.55E-05	0.445690	-4.15	0.721965	-
ECI1	chr16:2239401-2251587	NM_001919.4	8.25E-06	0.074890	4.13	1.635804	DUP
ATP6V1C2	chr2:10721629-10785110	NM_001039362.2	8.04E-05	0.486908	-4.11	0.707107	-

Table S3: RNA-seq expression outliers for the proband (III-3) in Family 1 filtered for those with absolute Z-score > 4.0.

Of the 21 outliers shown, 15 genes were upregulated and of these 11 lay on chromosome 16p13.3 within the duplication/triplication. We note that *ATP6V0C2* and *ATP6V1C2* both encode subunits of the vacuolar H^+-ATPase and there exists the possibility of a regulatory feedback loop leading to low expression of *ATP6V1C2*.

Gene	GRCh38 coordinates	GeneID	*P*-value	*P*-adjust	Z-score	FC	DUP/TRIP
ATP6V0C	chr16:2513952-2520218	ENSG00000185883	1.97E-13	3.87E-08	6.86	1.945310	TRIP
NDUFB2	chr7:140696708-140706643	ENSG00000090266	4.89E-11	4.80E-06	-6.68	0.619854	
RNPS1	chr16:2253120-2268126	ENSG00000205937	3.50E-10	2.29E-05	6.01	1.366040	DUP
RPS2	chr16:1962058-1964826	ENSG00000140988	5.47E-10	2.68E-05	5.80	1.670176	DUP
TSC2	chr16:2047985-2089491	ENSG00000103197	9.93E-10	2.94E-05	5.86	1.366040	DUP
TEDC2	chr16:2460109-2464963	ENSG00000162062	1.05E-09	2.94E-05	5.76	1.681793	TRIP
CCNF	chr16:2429447-2458854	ENSG00000162063	8.20E-10	2.94E-05	5.81	1.558329	TRIP
TBC1D24	chr16:2475127-2505730	ENSG00000162065	3.74E-09	9.17E-05	5.39	1.591073	TRIP
SDHAF3	chr7:97117698-97181763	ENSG00000196636	2.54E-08	0.000555	-6.00	0.517632	
NDUFB10	chr16:1959538-1961975	ENSG00000140990	5.26E-08	0.001032	5.18	1.443929	DUP
TRAF7	chr16:2155782-2178129	ENSG00000131653	1.08E-07	0.001929	5.05	1.337928	DUP
AMDHD2	chr16:2520371-2531417	ENSG00000162066	1.90E-07	0.003108	4.68	1.827663	TRIP
RAD50	chr5:132556977-132646349	ENSG00000113522	2.28E-07	0.003438	-5.31	0.673617	
TBL3	chr16:1972053-1982929	ENSG00000183751	3.05E-07	0.004278	4.83	1.375542	DUP
SLFN13	chr17:35435096-35448766	ENSG00000154760	3.71E-07	0.004859	-5.55	0.417544	
E4F1	chr16:2223591-2235742	ENSG00000167967	5.73E-07	0.007024	4.63	1.394744	DUP
FRG1	chr4:189940872-189963192	ENSG00000109536	8.24E-07	0.009518	4.66	1.404445	
CPNE1	chr20:35626044-35664900	ENSG00000214078	8.80E-07	0.009594	-5.35	0.334482	
ECI1	chr16:2239402-2251587	ENSG00000167969	1.49E-06	0.015427	4.46	1.505247	DUP
GFER	chr16:1984193-1987749	ENSG00000127554	4.18E-06	0.041001	4.30	1.464086	DUP
PGP	chr16:2211593-2214840	ENSG00000184207	5.10E-06	0.047688	4.25	1.404445	DUP

Table S4: RNA-seq expression outliers for Individual 2 filtered for those with absolute Z-score > 4.0.

Results are those using the MANE select transcript annotations rather than the original Genecode annotations. Of the 21 dysregulated genes, 16 were upregulated and of these, 15 lay on chromosome 16p13.3 within the duplication-triplication.

References

1. Watts, L.M., Bunyan, D.J., Giacopuzzi, E., Walker, S., Gazdagh, G., Thomas, N.S., Straub, V., Childs, A.M., Forsyth, J., Vogt, J., et al. (2024). FILIP1-associated neuromuscular disorder and phenotypic blending due to paternal UPD6. Brain Commun *6*, fcae330. 10.1093/braincomms/fcae330.
2. Roos, A., van der Ven, P.F.M., Alrohaif, H., Kolbel, H., Heil, L., Della Marina, A., Weis, J., Assent, M., Beck-Wodl, S., Barresi, R., et al. (2023). Bi-allelic variants of FILIP1 cause congenital myopathy, dysmorphism and neurological defects. Brain *146*, 4200-4216. 10.1093/brain/awad152.
3. Yu, J., Szabo, A., Pagnamenta, A.T., Shalaby, A., Giacopuzzi, E., Taylor, J., Shears, D., Pontikos, N., Wright, G., Michaelides, M., et al. (2022). SVRare: discovering disease-causing structural variants in the 100K Genomes Project. medRxiv, 2021.2010.2015.21265069. 10.1101/2021.10.15.21265069.
4. Pagnamenta, A.T., Yu, J., Willis, T.A., Hashim, M., Seaby, E.G., Walker, S., Xian, J., Cheng, E.W.Y., Tavares, A.L.T., Forzano, F., et al. (2023). A Palindrome-Like Structure on 16p13.3 Is Associated with the Formation of Complex Structural Variations and SRRM2 Haploinsufficiency. Human Mutation *2023*, 6633248.
5. Falace, A., Filipello, F., La Padula, V., Vanni, N., Madia, F., De Pietri Tonelli, D., de Falco, F.A., Striano, P., Dagna Bricarelli, F., Minetti, C., et al. (2010). TBC1D24, an ARF6-interacting protein, is mutated in familial infantile myoclonic epilepsy. Am J Hum Genet *87*, 365-370. 10.1016/j.ajhg.2010.07.020.
6. Guven, A., and Tolun, A. (2013). TBC1D24 truncating mutation resulting in severe neurodegeneration. J Med Genet *50*, 199-202. 10.1136/jmedgenet-2012-101313.
7. Campeau, P.M., Kasperaviciute, D., Lu, J.T., Burrage, L.C., Kim, C., Hori, M., Powell, B.R., Stewart, F., Felix, T.M., van den Ende, J., et al. (2014). The genetic basis of DOORS syndrome: an exome-sequencing study. Lancet Neurol *13*, 44-58. 10.1016/S1474-4422(13)70265-5.
8. Luthy, K., Mei, D., Fischer, B., De Fusco, M., Swerts, J., Paesmans, J., Parrini, E., Lubarr, N., Meijer, I.A., Mackenzie, K.M., et al. (2019). TBC1D24-TLDc-related epilepsy exercise-induced dystonia: rescue by antioxidants in a disease model. Brain *142*, 2319-2335. 10.1093/brain/awz175.
9. Mattison, K.A., Tossing, G., Mulroe, F., Simmons, C., Butler, K.M., Schreiber, A., Alsadah, A., Neilson, D.E., Naess, K., Wedell, A., et al. (2023). ATP6V0C variants impair V-ATPase function causing a neurodevelopmental disorder often associated with epilepsy. Brain *146*, 1357-1372. 10.1093/brain/awac330.
10. Collins, R.L., Glessner, J.T., Porcu, E., Lepamets, M., Brandon, R., Lauricella, C., Han, L., Morley, T., Niestroj, L.-M., Ulirsch, J., et al. (2022). A cross-disorder dosage sensitivity map of the human genome. Cell *185*, 3041-3055.e3025. 10.1016/j.cell.2022.06.036.
11. Finelli, M.J., Aprile, D., Castroflorio, E., Jeans, A., Moschetta, M., Chessum, L., Degiacomi, M.T., Grasegger, J., Lupien-Meilleur, A., Bassett, A., et al. (2019). The epilepsy-associated protein TBC1D24 is required for normal development, survival and vesicle trafficking in mammalian neurons. Hum Mol Genet *28*, 584-597. 10.1093/hmg/ddy370.
12. Tona, R., Chen, W., Nakano, Y., Reyes, L.D., Petralia, R.S., Wang, Y.X., Starost, M.F., Wafa, T.T., Morell, R.J., Cravedi, K.D., et al. (2019). The phenotypic landscape of a Tbc1d24 mutant mouse includes convulsive seizures resembling human early infantile epileptic encephalopathy. Hum Mol Genet *28*, 1530-1547. 10.1093/hmg/ddy445.
13. Tona, R., Lopez, I.A., Fenollar-Ferrer, C., Faridi, R., Anselmi, C., Khan, A.A., Shahzad, M., Morell, R.J., Gu, S., Hoa, M., et al. (2020). Mouse Models of Human Pathogenic Variants of TBC1D24 Associated with Non-Syndromic Deafness DFNB86 and DFNA65 and Syndromes Involving Deafness. Genes (Basel) *11*. 10.3390/genes11101122.

14. Liu, R., Xu, M., Zhang, X.Y., Zhou, M.J., Zhou, B.Y., Qi, C., Song, B., Fan, Q., You, W.Y., Zhu, J.N., et al. (2020). PDK1 Regulates the Maintenance of Cell Body and the Development of Dendrites of Purkinje Cells by pS6 and PKCgamma. J Neurosci *40*, 5531-5548. 10.1523/JNEUROSCI.2496-19.2020.
